# Effect of psychological first aid training for fellows on resident burnout and distress in the intensive care unit

**DOI:** 10.1371/journal.pone.0340456

**Published:** 2026-02-09

**Authors:** Haley Belt, Carol S. North, Traci N. Adams

**Affiliations:** 1 Division of Pulmonary and Critical Care Medicine, University of Texas Southwestern Medical Center, Dallas, Texas, United States of America; 2 Department of Psychiatry, University of Texas Southwestern Medical Center, Dallas, Texas, United States of America; Hokkaido University: Hokkaido Daigaku, JAPAN

## Abstract

**Background:**

Burnout is a common problem among medical trainees and can result in medical errors and intention to leave practice. Psychological first aid (PFA) is a form of mental health assistance provided after critical incidents with the goal of reducing distress and re-establishing functioning; the application of PFA to medical trainees has been suggested but studies are lacking.

**Methods:**

Pulmonary and critical care medicine fellows were given a 2-hour PFA training. Fellows were administered a survey immediately after training and again 3 months later. The effect of fellow PFA training on burnout and distress scores among medical residents supervised by pulmonary fellows was measured during medical intensive care unit (ICU) rotations and compared to ICU medical resident burnout and distress scores prior to PFA training.

**Results:**

Of the 11 fellows who completed the survey, 91% had a positive or extremely positive impression of the training. Six fellows noted a change of practice as a result of the training on the 3-month follow up survey. Resident burnout scores at the end of an ICU rotation were lower in the months following the PFA training compared to those at the end of an ICU rotation before the PFA training.

**Conclusions:**

This study demonstrates that bringing PFA training to trainees is both feasible and well-received and suggests that PFA training may impact trainees in settings that are high risk for burnout. The PFA training in this study may serve as a model for further attempts to educate healthcare workers in PFA.

## Introduction

The American Medical Association and American College of Graduate Medical Education have listed burnout prevention and management as organizational priorities, recognizing that *“*psychological, emotional, and physical well-being are critical in the development of the competent, caring, and resilient physician” [1,2]. Despite research efforts, burnout remains a substantial issue among medical trainees and can result in lower job satisfaction, intention to leave practice, and medical errors [3,4].

Residents in the intensive care unit (ICU) are particularly susceptible to burnout, as stressors such as codes, procedures, family meetings, and deaths abound in the ICU [5,6]. Interventions to reduce burnout that target trainees during their ICU rotations may therefore have a disproportionate impact on resident well-being.

Psychological first aid (PFA) is a form of mental health (MH) assistance provided in the immediate aftermath of disasters or other critical incidents to address acute distress and re-establish effective coping and functioning [5,7,8]. Developed through expert consensus, PFA is flexible for use in various settings, populations, and cultures [5,7,8]. Although several articles have suggested that PFA programs may be beneficial for ICU providers [5,9], data to support its implementation in the ICU or among medical residents is lacking. The purpose of this study is to assess the effect of a PFA curriculum for pulmonary/critical care fellows (PCCM) on burnout levels in medical residents during their medical ICU (MICU) rotation.

## Methods

**Fig 1:**
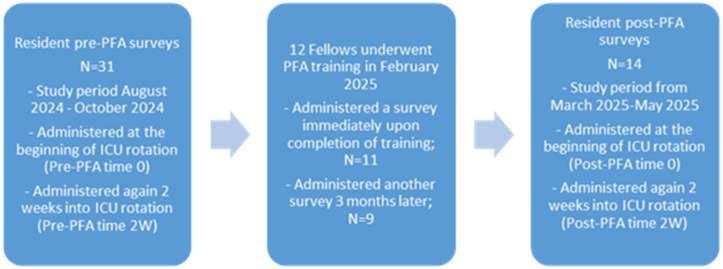
Resident burnout and visual analog distress score survey timing.

The study procedures are summarized in Fig 1. This study involved provision of PFA training for PCCM fellows and collecting surveys immediately upon completion of the PFA training and again 3 months after completion of the training. It further involved collecting burnout and stress data from medical residents working with these fellows at the beginning of their MICU rotation and again 2 weeks into the rotation. No burnout or distress data was collected from fellows who completed the PFA training because the intent was to evaluate whether educating the fellows in PFA allowed them to practice PFA or address burnout and distress with the residents on their ICU teams. The medical resident burnout and stress data were assessed during 3 months prior to fellow PFA training and again during 3 months shortly after fellow PFA training, as described in detail below.

### PFA training

On February 15 and 26 of 2025, PCCM fellows participated in a 2-hour PFA training session given by a licensed clinical social worker (LCSW) in the Office of Faculty Wellness at UTSW. Each fellow attended only one of the 2-hour sessions. The PFA training was based on a 4-hour workshop previously given by the same PFA training presenter to faculty physicians and advanced practice providers in the peer-to-peer support program at UTSW. Peer-to-peer PFA training that had been successfully provided to faculty was modified for fellows to condense the presentation from 4 hours to 2 hours to accommodate the time constraints of fellows on clinical rotations. Case examples for discussion from the 4-hour faculty presentation were revised by the study authors in the 2-hour fellows’ presentation to represent more general ICU-specific scenarios. Topics covered during the 2-hour curriculum included an overview of the evidence behind peer support programs, introduction of stressors prevalent among healthcare providers, and elements of PFA peer support based on previously published protocols from the American Red Cross [7] that cover supportive listening, and exploration of emotions. Role-playing activities for practice the elements of PFA were also included in the training. Slides for the PFA training and case-based discussion prompts are provided in S1 and S2 Appendices, respectively. Half of the clinical fellows were assigned to each session, and coverage for inpatient rotations was provided during the 2-hour training by fellows not assigned to that day’s training.

### Sampling

All 15 fellows in the clinical portion of their fellowship were invited to participate in the training via email. Only fellows in the clinical portion of their fellowship training participated in the PFA training; those in their research years were excluded as they no longer interact with medical residents regularly and have research obligations that precluded attendance at a 2-hour training. Of the 15 fellows invited to participate, 12 (80%) attended. Because the goal of the study was to assess the impact of PFA training on burnout and distress among medical residents working with fellows who had received the PFA training, residents rather than fellows were chosen for measurement of burnout and stress outcomes. By providing PFA training to fellows, this study equipped PCCM fellows to respond to distress among the residents. Further, the number of fellows involved in this intervention was too small to provide statistical power to detect meaningful changes in burnout and distress scores.

All UTSW residents starting their clinical rotations in the MICU at Parkland Health and Hospital System (PHHS) or Clements University Hospital (CUH) were recruited in person to the study in 2 cohorts: the first (N = 87 residents in the MICU in that period, of whom 31 participated in the study for a 36% participation rate) from August-October 2024, prior to the PFA training, and the second (N = 74 study-eligible residents who worked with PFA-trained fellows from a total of 102 residents rotating in the MICU during that period, 14 participated in the study for a 19% participation rate) from March-May 2025, after the PFA training.

This study was considered non-regulated human subjects research under the category of quality improvement by the Institutional Review Board at UTSW and PHHS. The IRB approval letters are included in the submission (S3 and S4 Appendices, respectively). Participation of both residents and fellows was voluntary. Participation in the survey served as consent to participate in the study because the data were analyzed anonymously and no identifiable information was obtained.

### Instruments of measure

#### Fellows’ evaluation of the PFA curriculum.

Hand-written surveys for evaluation of the PFA curriculum were completed by the fellows who participated in the PFA training and derived from Moore’s expanded outcome framework [10]. Moore level 1 (participation) was assessed through attendance records from the training. Moore levels 2 (satisfaction), 3A (declarative knowledge), 3B (procedural knowledge), and 4 (competence) were assessed immediately following the training (S5 Appendix). Moore level 5 (performance) was assessed in a 3-month follow up survey administered 3 months after the training (S6 Appendix). A 3-month interval for post-training surveys was chosen, as 3 months is a common interval in the literature for knowledge retention after medical training and this ensured completion of the surveys before the end of the academic year and fellowship graduation, after which survey participation would likely diminish.

#### Assessment of residents’ burnout and stress levels.

Resident burnout and stress were assessed through surveys including an abbreviated Maslach Burnout Inventory (aMBI) and 100-point visual analog distress scale (VADS), to evaluate Moore level 6 (focused on resident wellbeing rather than patient outcomes indicated by the original Maslach instrument) [11]. The aMBI score is divided into 3 components: personal accomplishment (PA), emotional exhaustion (EE), and depersonalization (D) [11]. Additionally, residents filled out a demographic questionnaire that was also administered along with these scales at the time of their first surveys (S7 Appendix).

### Data collection procedures

Fellows’ evaluations of the PFA intervention (see above) were assessed immediately after the presentation and 3 months later. They were not assessed for their burnout and distress levels.

The residents were administered the aMBI or VASD scales for assessment of their burnout and distress levels. These hand-written resident surveys were completed at the beginning of their MICU rotation and again 2 weeks into the rotation. A 2 week interval was chosen as some residents and fellows have only a 2 week ICU rotation. The first cohort of residents completed their surveys in August-October 2024 before any PFA training had been conducted (so that their associated fellows had not received the PFA intervention) and the second cohort of residents completed these surveys in March-May 2025 soon after their associated fellows had completed PFA training.

### Statistical analysis

Based on power calculations, a sample of 32 residents (including 16 in the cohort whose fellows had not received PFA training and 16 in in the cohort whose fellows had received PFA training) was needed to detect a 2-point change in the survey scales and subscales that was considered clinically relevant based on prior burnout inventory data from US physicians [12]. Assuming 25–30 residents per month rotating in the MICU and an expected 20–30% response rate consistent with other resident survey data [12], data were collected for 3 months from each resident cohort.

Descriptive statistics including mean, standard deviation, median, and interquartile range were used to evaluate demographic surveys. Score totals for the aMBI were calculated by adding each component score (emotional exhaustion, depersonalization, and personal accomplishment). The PA score (maximum value = 18) was inverted for the purpose of analysis because lower scores on the instrument indicate higher burnout; the emotional exhaustion and depersonalization components were already valued for higher scores representing higher levels of burnout (S2 Appendix). Normative distribution was confirmed by histogram and paired t-tests and unpaired t-tests where appropriate were used to make the following comparisons: beginning of the MICU rotation vs 2 weeks into the rotation and pre-PFA vs. post-PFA resident cohorts. Residents with missing data for VAS were excluded from VAS comparisons but were included in the aMBI comparison, and residents with missing data for aMBI were excluded from aMBI comparisons but were included in the VAS comparison.

The dataset was de-identified and stored in a password-protected Microsoft Excel spreadsheet on the UTSW network. This study was considered non-regulated human subjects research under the category of quality improvement by the Institutional Review Board at UTSW and PHHS (S5 and S6 Appendices respectively). Statistical analysis was performed using Graph Pad Prism (Boston, MA).

## Results

### PFA training evaluations by fellows

Table 1 provides results of the fellows’ immediate post-training PFA training evaluations. Of the 11 fellows who completed the survey, 91% had a positive or extremely positive impression of the training, with 91% agreeing or strongly agreeing that the course achieved its learning objectives and 100% agreeing or strongly agreeing that PFA is a valuable tool for clinicians. After completing PFA training, nearly two-thirds felt very confident or extremely confident about performing PFA with MICU residents, and virtually all agreed or strongly agreed with the statement that the course would lead to a change in their practice.

**Table 1:** Fellows’ PFA evaluation surveys immediately after PFA training (N = 11).

Survey item	n (%)
Overall impression of the training	
Extremely negative	0 (0)
Negative	0 (0)
Neutral	1 (9.1)
Positive	5 (45.5)
Extremely positive	5 (45.5)
Impression of presenter	
Extremely negative	0 (0)
Negative	0 (0)
Neutral	1 (9.1)
Positive	2 (18.2)
Extremely positive	8 (72.3)
Course achieved learning objectives	
Strongly disagree	0 (0)
Disagree	0 (0)
Neutral	1 (9.1)
Agree	5 (45.5)
Strongly agree	5 (45.5)
PFA is a valuable tool for ICU clinicians	
Strongly disagree	0 (0)
Disagree	0 (0)
Neutral	0 (0)
Agree	1 (9.1)
Strongly agree	10 (90.9)
Course will lead to a change in my practice	
Strongly disagree	0 (0)
Disagree	0 (0)
Neutral	1 (9.1)
Agree	4 (36.4)
Strongly agree	6 (54.5)
Confidence in performing PFA with residents in the ICU	
Not at all confident	0 (0)
Slightly confident	1 (9.1)
Confident	3 (27.3)
Very confident	3 (27.3)
Extremely confident	4 (36.4)

Table 2 presents results of the fellows’ 3-month PFA training evaluation surveys, which were competed by 75% of the fellows who participated in the training. Only about one-fifth of these fellows said they had provided PFA to someone prior to PFA training but more than one half indicated that they had provided PFA since the training. Two-thirds reported they had made a change in their practice as a result of the training. One-third felt effective or extremely effective in performing PFA.

**Table 2:** Fellows’ PFA evaluation survey 3 months after PFA training (N = 9).

Survey question	n (%)
Prior to completing the psychological first aid training, had you ever provided psychological first aid in the intensive care unit?	
Yes	2 (22.2)
No	7 (77.8)
If you have previously provided PFA, how often?	
One time	0 (0)
Once a month	1 (11.1)
Once a week	1 (11.1)
Daily	0 (0)
I have not provided PFA	7 (77.8)
Have you provided psychological first aid with anyone in the ICU since your training?	
Yes	5 (55.6)
No	4 (44.4)
If you have provided PFA since the PFA training, how often?	
One time	1 (11.1)
Once a month	2 (22.2)
Once a week	2 (22.2)
Daily	0 (0)
I did not provide PFA	4 (44.4)
Who have you used psychological first aid with?*	
Patients	1 (11.1)
Family members of patients	2 (22.2)
Residents	5 (55.6)
Fellows	4 (44.4)
Faculty	0 (0)
None of the above	4 (44.4)
Did the psychological first aid training change your practice?	
Yes	6 (66.7)
No	3 (33.3)
How effective are you in performing psychological first aid?	
Extremely ineffective	0 (0)
Ineffective	1 (11.1)
Neutral	4 (44.4)
Effective	3 (33.3)
Extremely effective	0 (0)
No answer	1 (11.1)
If you provided psychological first aid since your training, did you feel that it helped your colleague, patient, or patient’s family member?	
Yes	5 (55.6)
No	0 (0)
Did not provide PFA	5 (55.6)

* Fellows were asked to circle all that apply, thus totals are > 100%.

### Resident survey results for burnout and distress

A total of 45 MICU residents participated in the surveys, including 31 residents working with fellows without PFA training and 14 working with fellows shortly after they received PFA training. Demographic characteristics of these residents are presented in Table 3.

**Table 3:** Demographic characteristics of ICU residents completing surveys.

Variable	Residents without fellow PFA training (N = 31)	Residents with fellow PFA training (N = 14)
Male gender, n (%)	17 (45.9)	4 (28.5)
Age, mean (SD)	28.6 (2.0)	29.8 (3.7)
Race, N (%)		
White	13 (41.9)	6 (42.9)
Black	3 (9.7)	3 (21.4)
Asian	14 (45.2)	4 (28.5)
Other	1 (3.2)	1 (7.1)
Hispanic ethnicity, n (%)	3 (9.7)	0 (0)
Married, n (%)	14 (45.2)	5 (35.7)
Living with children, n (%)	7 (22.6)	3 (21.4)
Year started residency, n (%)		
2022	9 (29.0)	2 (14.3)
2023	17 (45.9)	7 (50.0)
2024	6 (19.4)	5 (35.7)
Planning to pursue fellowship in PCCM, n (%)		
Yes	7 (22.6)	2 (14.3)
No	24 (77.4)	12 (85.7)
Type of residency program, n (%)		
Internal medicine	26 (83.4)	14 (100.0)
Family medicine	4 (12.9)	0 (0)
Neurology	1 (3.2)	0 (0)
Dermatology	0 (0)	0 (0)
Time spent in MICU this academic year, n (%)		
< 1 month	14 (45.2)	4 (28.5)
1–3 months	16 (51.6)	10 (71.4)
> 3 months	1 (3.2)	0 (0)
First ever MICU rotation, n (%)		
Yes	8 (25.8)	4 (28.5)
No	23 (74.2)	10 (71.4)
Do you know how to access psychiatric care at UTSW?, n (%)		
Yes	21 (67.7)	11 (78.6)
No	10 (32.3)	3 (21.4)
Do you know a healthcare worker at UTSW who could not access psychiatric care in a timely manner?, n (%)		
Yes	3 (9.7)	1 (7.1)
No	28 (90.3)	13 (92.9)

Table 4 presents residents’ aMBI and VADS scores for residents who worked with fellows who had not received PFA training and residents who worked with fellows shortly after they had received PFA training. At the start of the MICU rotation, there was no significant difference in aMBI or VADS scores between the 2 groups. Residents who worked with non-PFA trained fellows (i.e., completed surveys in the 3 months prior to PFA training) demonstrated worsening aMBI scores, particularly worsening aMBI D score, during the rotation compared to the start of the rotation. However, residents working with PFA-trained fellows (i.e., completed surveys in the 3 months after PFA training) did not show worsening aMBI scores during the rotation compared to the start of the rotation. The increase in VADS score during the rotation in the residents working with fellows who did not receive PFA training fell short of statistical significance, and the 2 groups of residents did not differ in VADS scores during their rotations.

**Table 4:** Resident aMBI and VADS scores.

Scale score	Residents without fellow PFA training Mean (SD) N = 31	Residents with fellow PFA training Mean (SD) N = 14	P-value for row
aMBI total*			
Before rotation	18.1 (8.2)	19.1 (8.0)	0.71
2 weeks into rotation	22.0 (7.5)	18.1 (8.8)	0.13
*P-value for time frame*	*0.002*	*0.50*	
**aMBI PA**			
Before rotation	13.4 (2.7)	14.1 (2.9)	0.45
2 weeks into rotation	13.1 (2.9)	14.2 (3.4)	0.44
*P-value for time frame*	*0.89*	*0.82*	
**aMBI D**			
Before rotation	4.8 (4.2)	5.6 (3.8)	0.53
2 weeks into rotation	9.5 (5.2)	5.0 (3.3)	0.005
*P-value for time frame*	*<0.001*	*0.41*	
**aMBI EE**			
Before rotation	8.7 (3.5)	9.5 (3.7)	0.48
2 weeks into rotation	8.0 (4.5)	9.3 (4.9)	0.36
*P-value for time frame*	*0.30*	*0.79*	
**VADS**			
Before rotation	56.7 (15.9)	53.5 (17.1)	0.47
2 weeks into rotation	61.2 (16.7)	52.7 (20.0)	0.15
*P-value for time frame*	*0.11*	*0.88*	

Note: aMBI = abbreviated Maslach Burnout Inventory; VADS = visual analog distress scale.

* aMBI component categories include personal accomplishment (PA), depersonalization (D), and emotional exhaustion (EE). The aMBI total score is calculated by adding aMBI D, EE, and (-) PA score because the PA score is inverted with higher scores indicating lower levels of burnout with 18 as its maximum score; for aMBI D and EE scores, higher scores indicate higher levels of burnout.

## Discussion

This study evaluated a PFA curriculum for PCCM fellows using Moore’s expanded outcomes framework. The training was well-attended with high learner satisfaction scores, self-reported knowledge gains and confidence. Two-thirds of PFA training participants indicated that they had a practice change as a result of the training in a 3-month follow up survey. Resident burnout scores at the end of an ICU rotation were lower in the months following the PFA training compared to those at the end of an ICU rotation before the PFA training; residents working with fellows who did not receive PFA training had significant increases in burnout, but residents working with fellows who received PFA training did not.

The improved depersonalization score of the aMBI was the primary driver of the change in aMBI score following PFA training. Depersonalization is a form of dissociation, which was first described as a pathological process involving discontinuity in the normal integration of mental functions. Dissociative disorders outlined in the *Diagnostic and Statistical Manual, 5*^*th*^ *edition,* were previously thought to arise from traumatic experiences [13], but the term “dissociation” has also been used in general discourse to describe normative mental processes such as narrowly focused attention and losing track of time [14–17]. This nonpathologic form of dissociation, rather than the pathological dissociative disorders, may also occur in the context of trauma [14–17]. While PFA is not designed to treat dissociation or depersonalization specifically, PFA for individuals with experiences of depersonalization or dissociation endorsed on self-report questionnaires may assist in reducing distress burden by implementing calming techniques and building resilience similar to PFA’s benefits for other types of stress responses.

VADS scores did not change during the ICU rotation and were not improved after PFA training. VADS score is merely a snapshot of individual distress levels, which can vary substantially due to factors including lack of sleep, work stressors, and home stressors. PFA, which provides point of care reduction in distress levels, may not be sufficient to diminish VADS scores at 2 weeks but could potentially reduce VADS scores immediately following a stressful work-related event such as a code or patient death. Future studies could consider measurement of VADS scores following acute work stressors to more accurately capture changes in distress levels associated with implementation of PFA.

PFA is derived from expert opinion and supports the development of supportive listening skills, coping strategies, and warning signs that formal psychiatric evaluation is necessary [5,7]. The UTSW curriculum was led by an LCSW and was condensed into a 2-hour training session for PCCM clinical fellows with discussion cases tailored for the ICU. Based on the higher burnout reported by residents working with fellows who received PFA training than by residents working with fellows who did not have this training, the PFA training showed clear evidence of success. The PFA training appears to have empowered fellows to use PFA with their MICU residents, which may have contributed to less development of burnout in their residents. This study provides empirical data supporting suggestions in published editorials that PFA would be of benefit to ICU providers [5,9]. Programs to teach PFA to healthcare professionals are already available but can be time-consuming and may require travel and funding [18–20]. This study demonstrates that bringing PFA training to trainees is both feasible and well-received and suggests that PFA training may impact trainees in settings that are high risk for burnout. The PFA training in this study may serve as a model for further attempts to educate healthcare workers in PFA.

An important limitation of this study is the small sample size, particularly in the post-PFA training resident cohort. Post-PFA residents had a lower sample size due to many residents declining to participate in the surveys, which may create a research bias, and due to inclusion of only those residents who were on the teams of fellows who had participated in PFA training. However, despite this sample size limitation, significant benefits were detected in residents working with fellows who had received PFA training regarding burnout but not distress. Busy MICU residents with variable schedules are difficult to recruit for survey-based studies, and the relatively low participation rate may have led to sampling error and research bias. Further, generalizability may be limited as this is a single-center study and participants who voluntarily enrolled in the study may hold a more favorable view of the proposed intervention or depend on the institution for evaluations and letters of recommendation and, consequently, exhibit more positive attitudes when responding to the follow-up survey.

Finally, due to the small sample size, we were unable to control for factors that may have affected a change in burnout scores such as the time of year; however, similar baseline scores between groups indicate that these variables may not have substantially affected burnout scores. The study design avoided collecting data around the holidays (November-January), as holidays may exacerbate burnout. In pursuit of research efficiency by this study, the design was not a randomized controlled study assigning random subsamples of residents to intervention and non-intervention groups. It is possible that differences in time frame (such as the time of year) and pre-existing differences in the resident and fellow cohorts in this study. Future randomized controlled experimental studies are needed to confirm that the apparent benefits found by this study can be confidently attributed to the intervention and perhaps include refresher courses or a longer training to address the finding that some fellows did not feel confident or effective in performing PFA as a result of the training.

In summary, a PFA training for PCCM fellows was found to be associated with reduced evidence of burnout among residents rotating in the MICU. Future research, including randomized controlled trials of PFA, expansion to other academic medical centers and other medical subspecialties, and refinement of the intervention through testing of effectiveness of its material, and application to other groups such as faculty and staff, is needed.

## Supporting information

S1 Appendix Slides for PFA training. (PDF)

S2 Appendix PFA training case-based discussion prompts. (DOCX)

S3 Appendix Institutional Review Board approval letter for University of Texas Southwestern. (PDF)

S4 Appendix Institutional Review Board approval letter for Parkland. (PDF)

S5 Appendix Moore’s expanded outcomes framework. (DOCX)

S6 Appendix 3-month follow-up survey. (DOCX)

S7 Appendix Resident demographic form. (DOCX)

S1 File Deidentified data. (XLSX)
