# Supplementary material for: Effect of psychological first aid training for fellows on resident burnout and distress in the intensive care unit
Source: PLoS One. 2026 Feb 9;21(2):e0340456. doi: 10.1371/journal.pone.0340456 (PMC12885303; doi:10.1371/journal.pone.0340456)
Supplement: S1 Appendix — (PDF) [file pone.0340456.s001.pdf]

C.A.L.M.

Cultivating
Active
Listening in
Medicine

Peer to Peer Support Program

Presented by: Jaime Harry, MSW, LCSW

February 12th, 2025

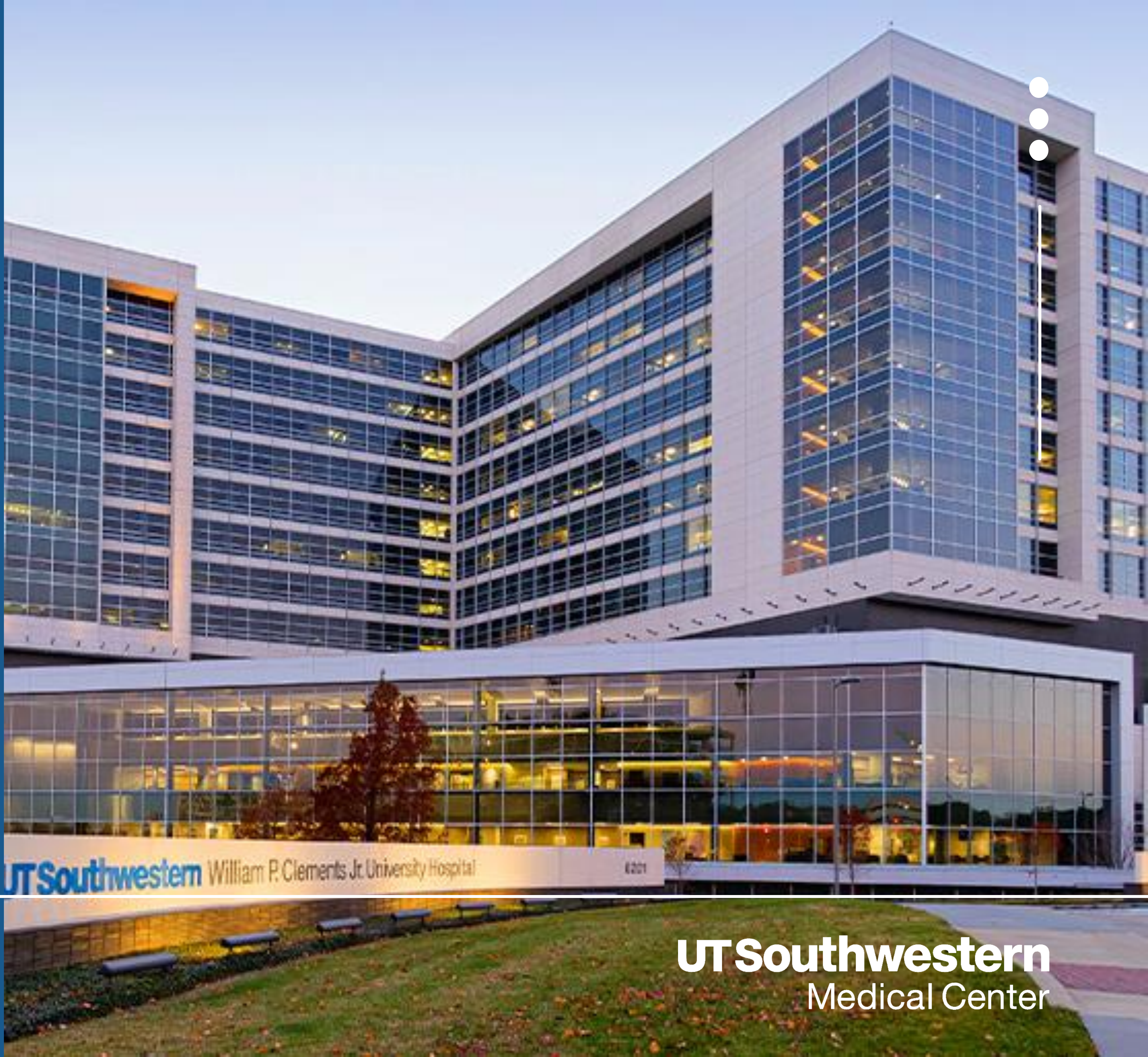

C.A.L.M. Leadership Team

Executive Sponsor and Program Champion:

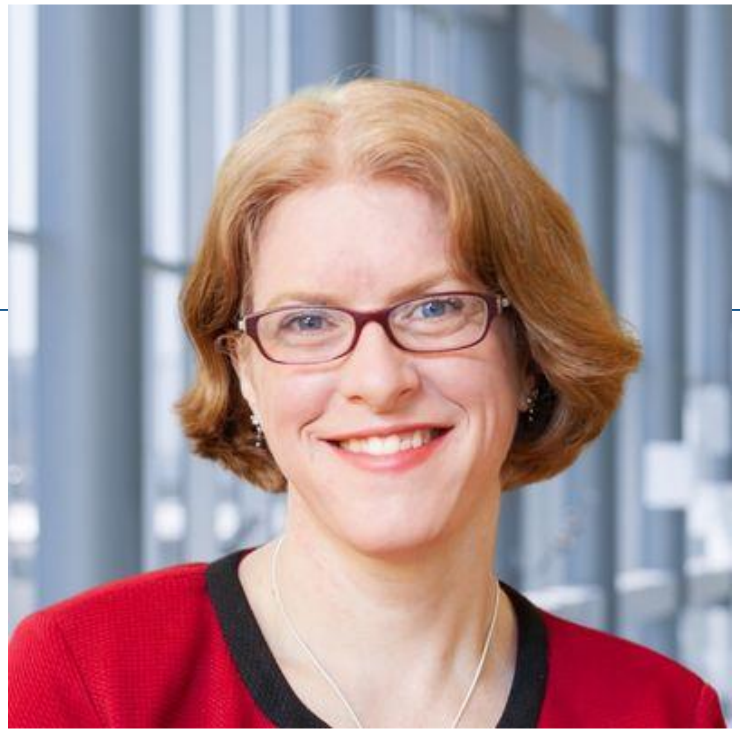

Susan Matulevicius, MD, MSCS,

Associate Dean of Faculty Wellness

Executive Advisor and Program Champion:

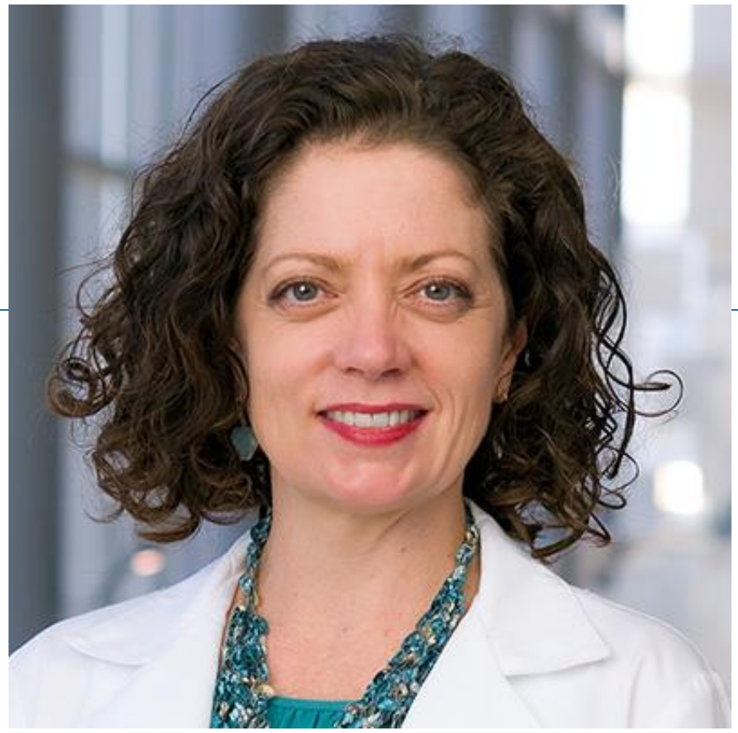

Laura Kirk, MSPAS, PA-C

Assistant Director Advance Practice Providers

CALM Program Director and Certified Trainer:

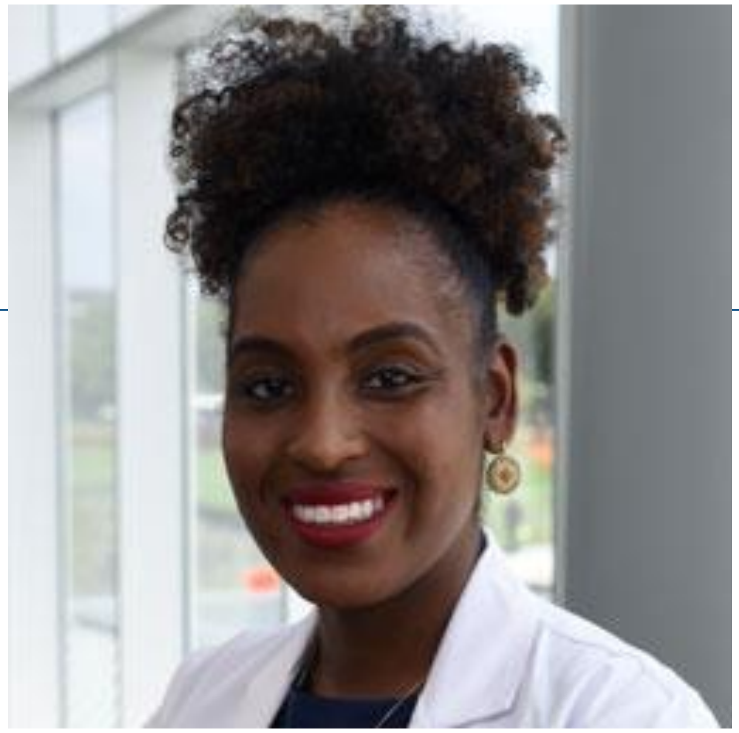

Jaime Harry, MSW, LCSW

Licensed Clinical Social Worker

UTSouthwestern

Center for Patient Safety

Caring for Our Own Train-the-Trainer Workshop

Mission: REDUCING PREVENTABLE HARM

Established in 2005,
Independent not-for-profit

Patient safety and quality resources and service hospitals, health systems, ambulance services, physician offices, nursing homes, home health & hospice.

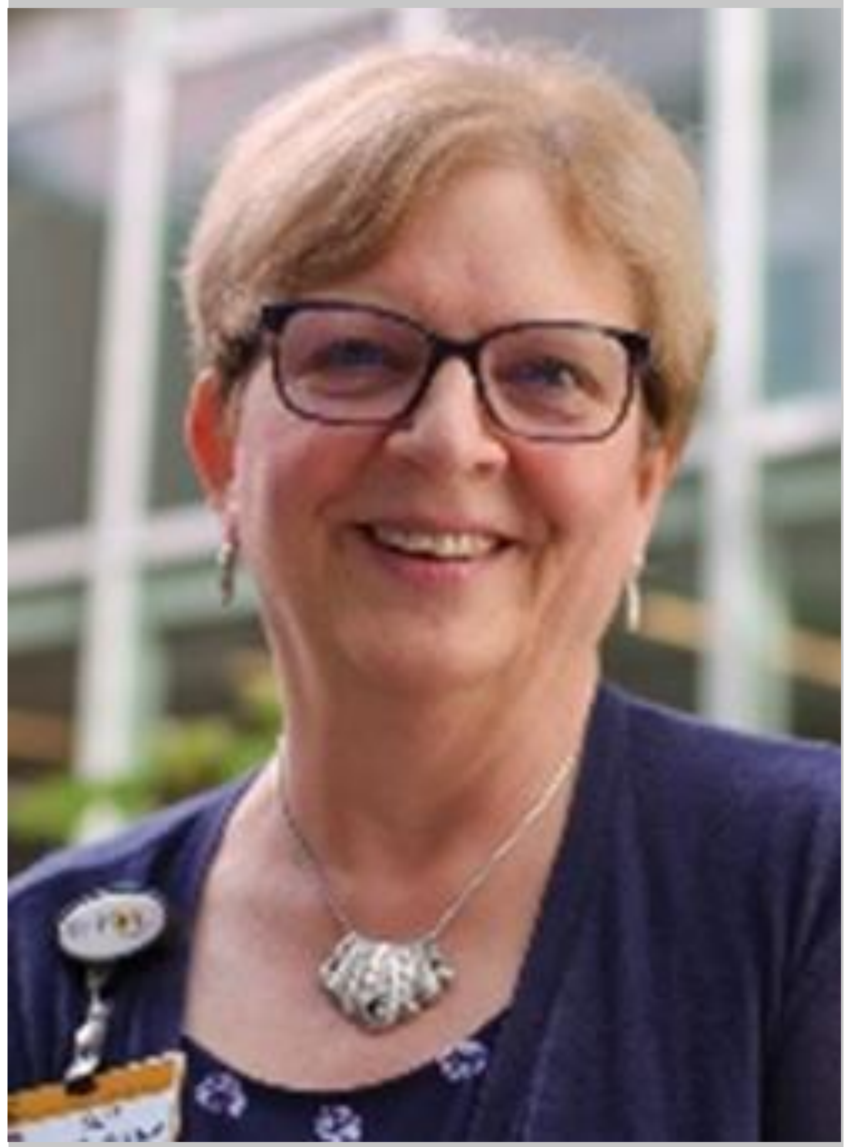

Susan D. Scott, PhD., RN, CPPS, FAAN

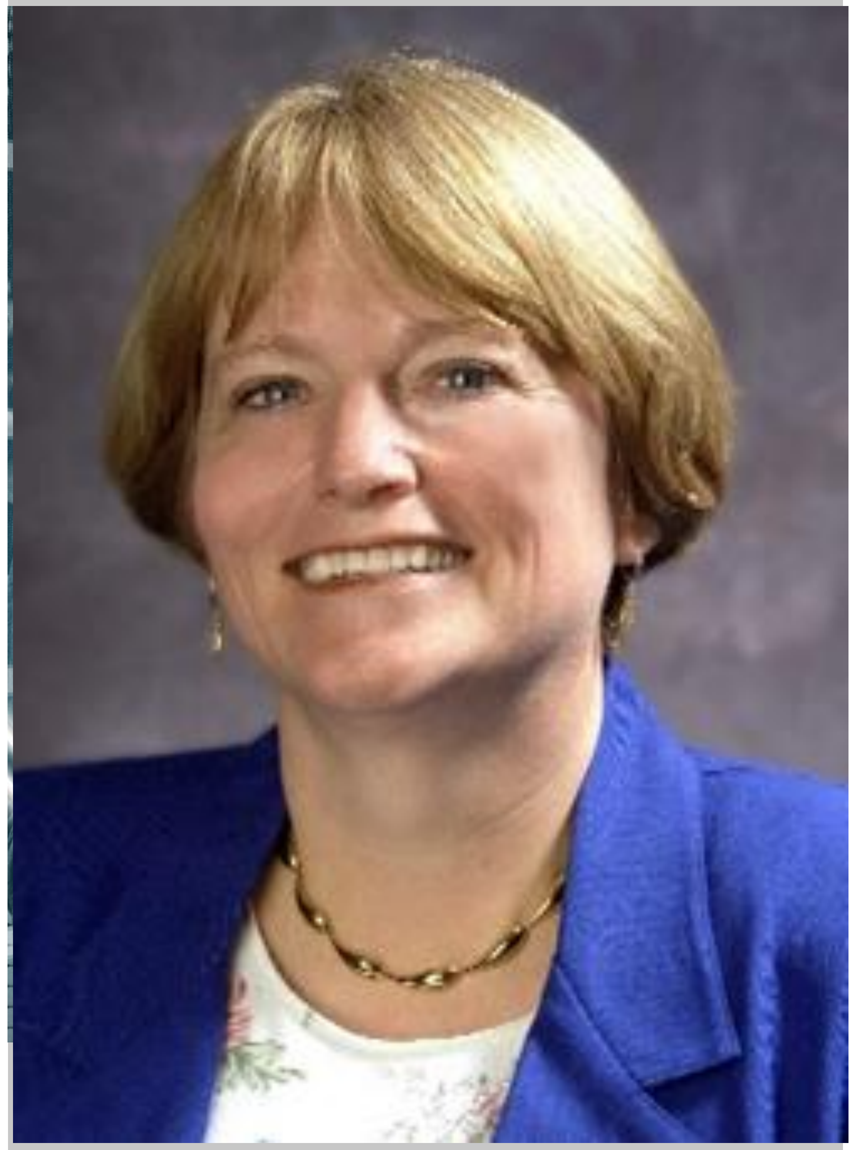

Eunice Halverson, MA, CPPS

AGENDA

I. What is Peer Support?

II. Second Victim Phenomena

III. CALM Peer Support Skills

IV. Applying Skills to Stages of Second Victim Trauma

Get ready to actively participate!

INTRODUCTIONS

ABOUT YOU:

- Name and clinical background
- What do you hope to gain from today's training?

Program Objectives

Connect

Understand

Recognize

Develop & Apply

Learn

BEFORE WE BEGIN

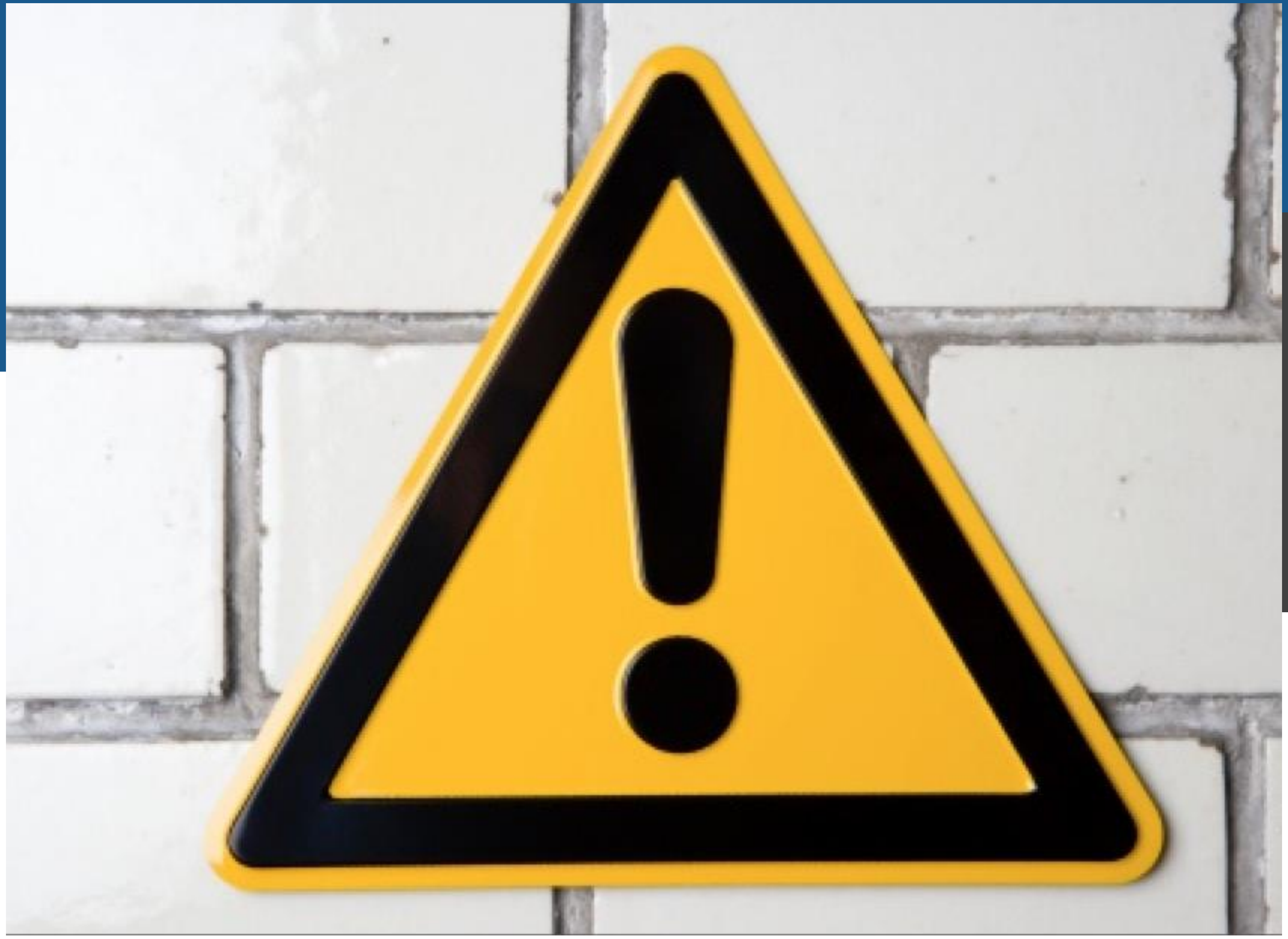

Trigger Warning

Peer Support Defined:

Peer Support =

a supportive relationship between people who have a lived experience in common in relation to either their own mental health challenge or illness or that of a loved one.

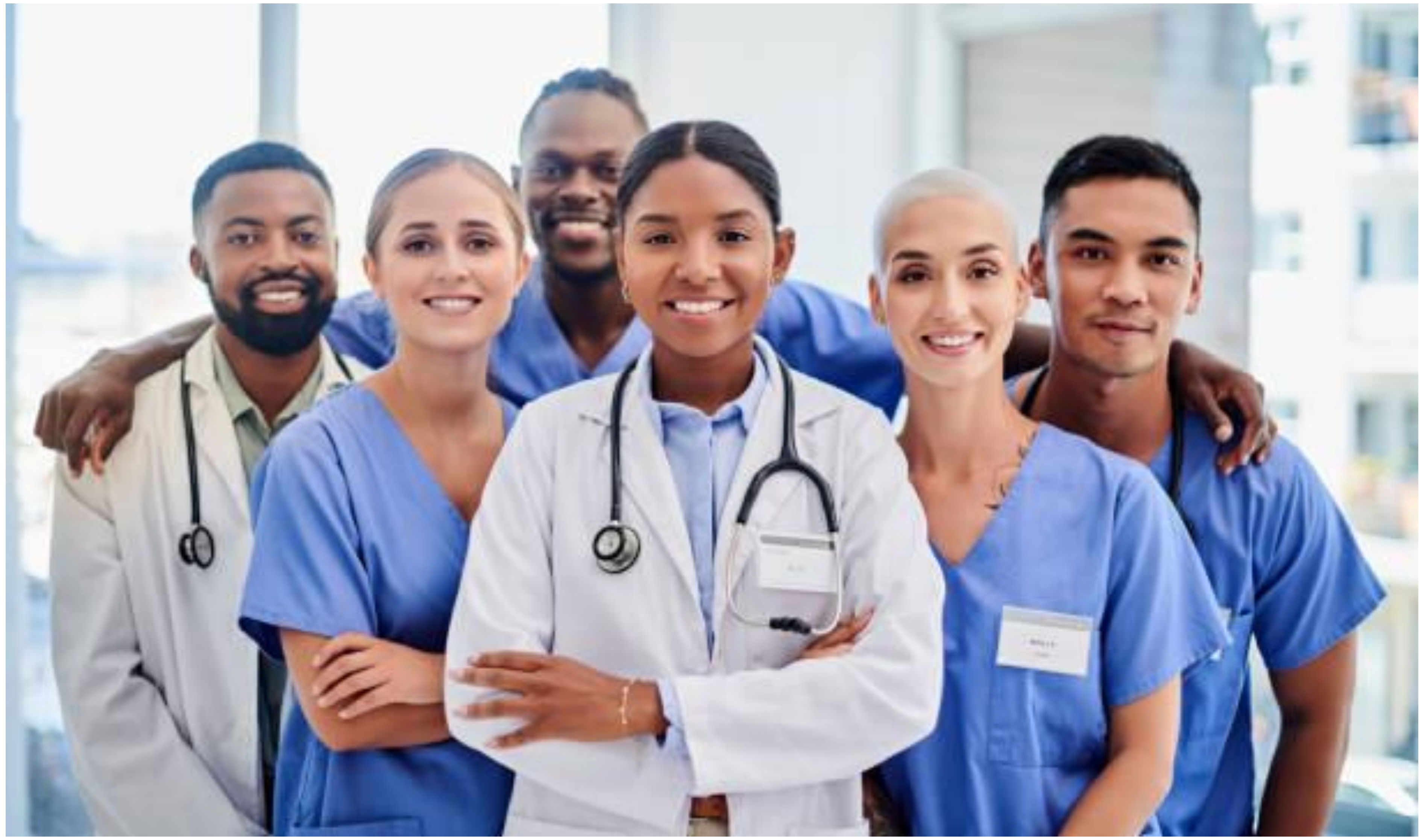

Peer Support is NOT:

- Interrogating the Peer
- Investigating the Event
- Judging
- Giving Advice
- Counseling

Peer Support IS:

- Personalized 1:1 engagement
- Timely
- Active listening by a peer
- Non-judgmental approach
- Emotionally supportive presence

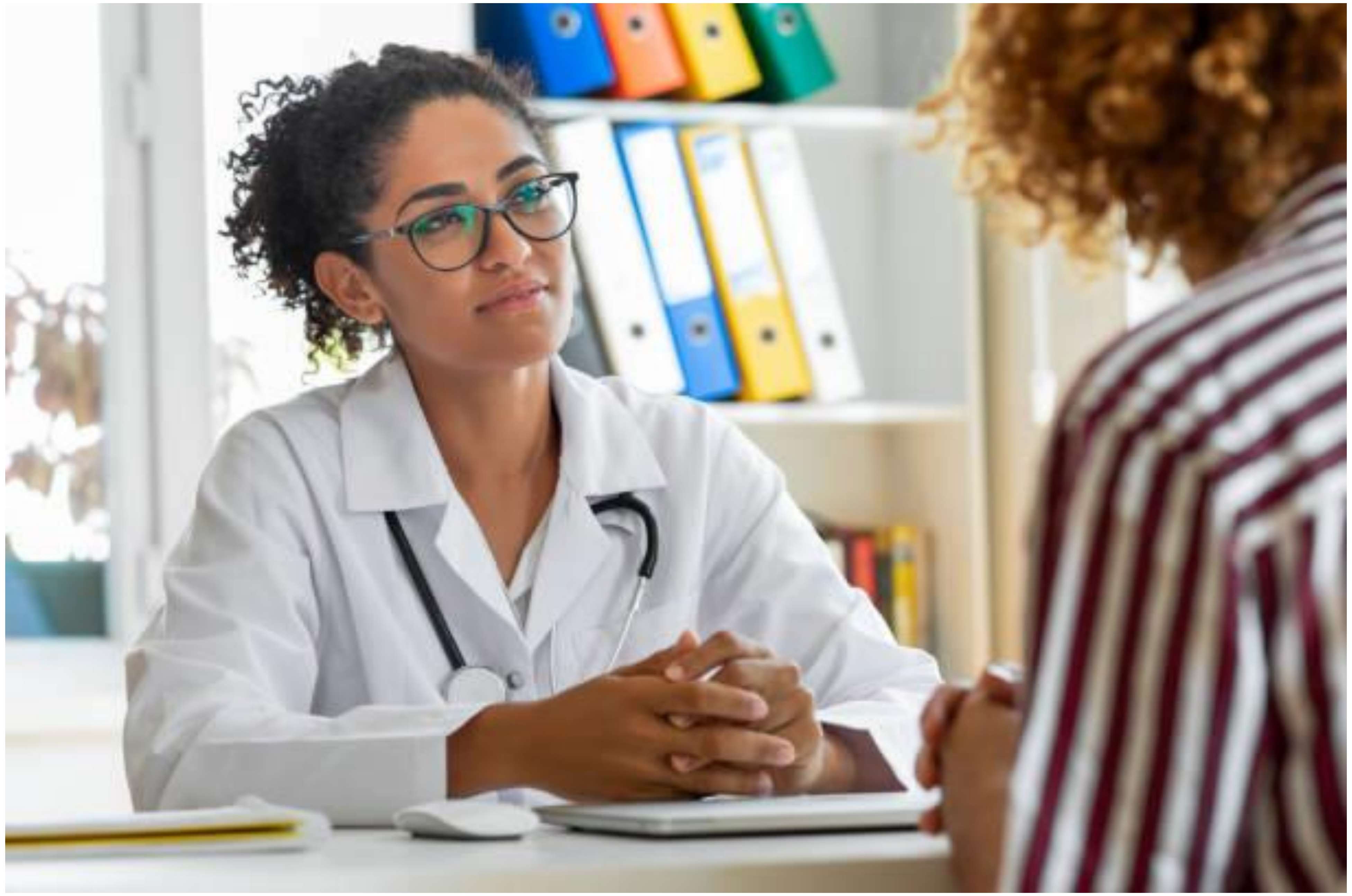

Why is Peer Support Needed

Healthcare Professionals may face emotional and psychological trauma due to their exposure to critical incidents and daily life stressors

Trained peers who are occupationally aware and may have similar lived experiences can

- Listen
- Offer non-professional support
- Refer colleagues for professional support when needed

Providing peer support services to our Healthcare Professionals and training skilled peer support providers can foster a culture of psychological safety and improve the mental health and wellbeing of our UT Southwestern workforce.

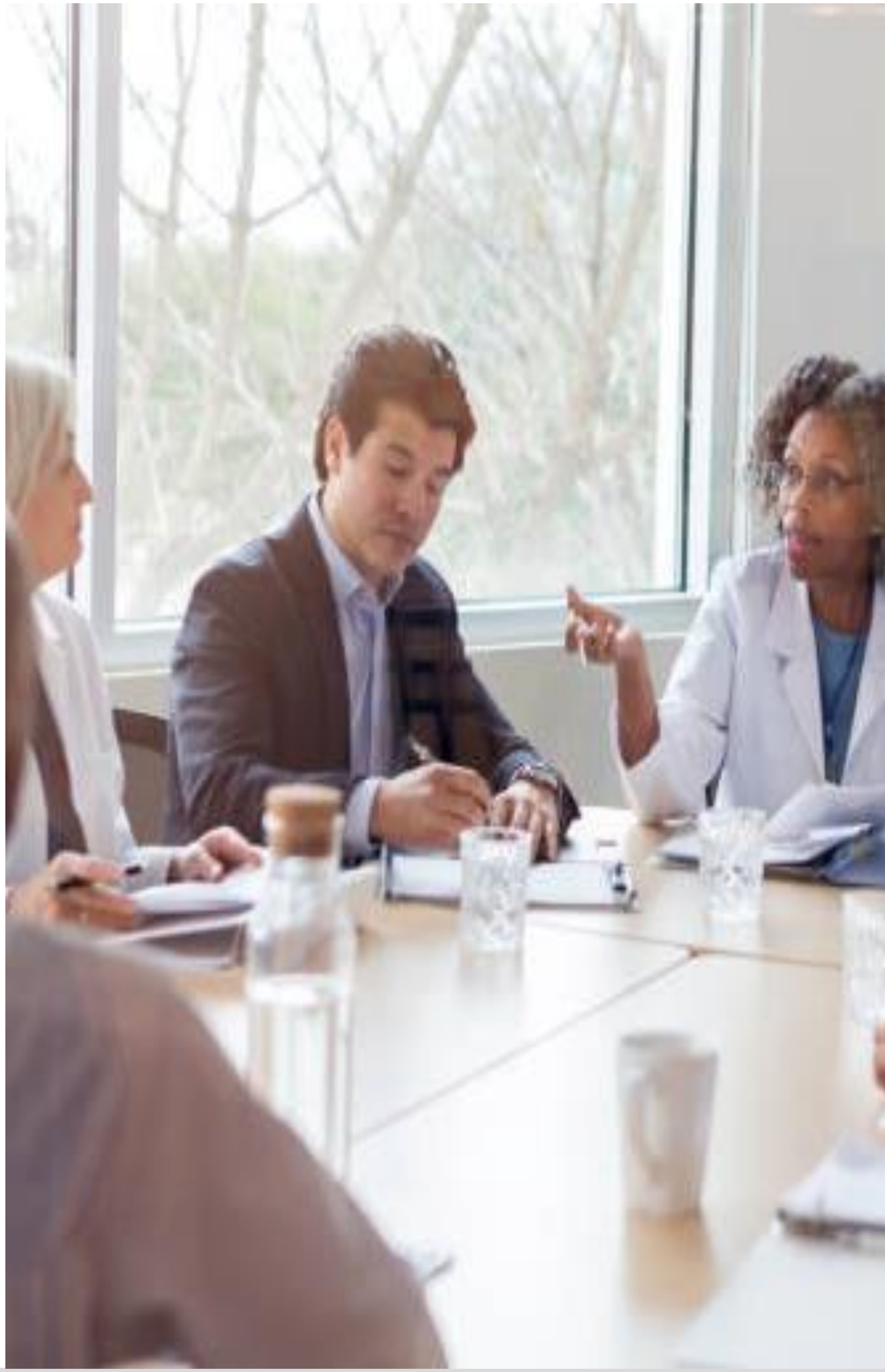

UTSW Aligns with the mission of Peer Support

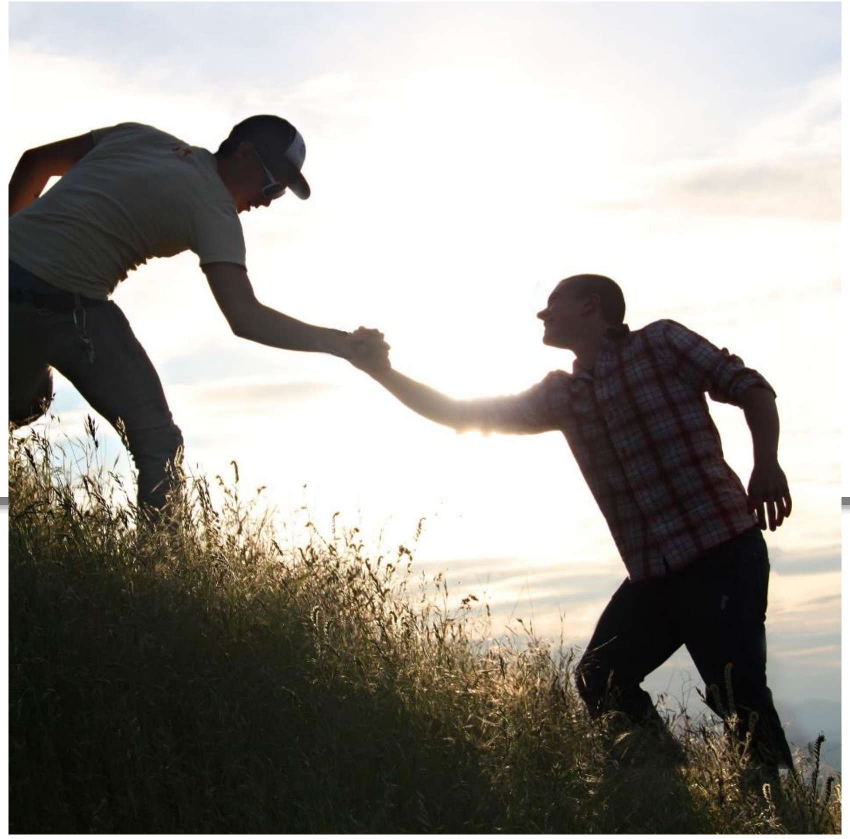

Support

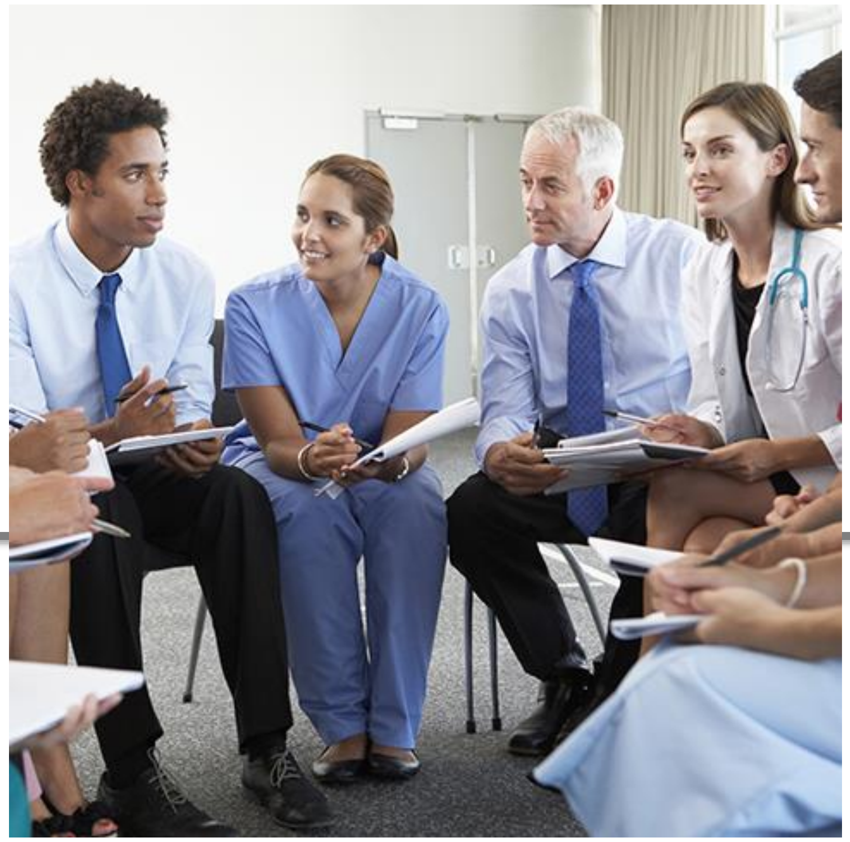

Educate

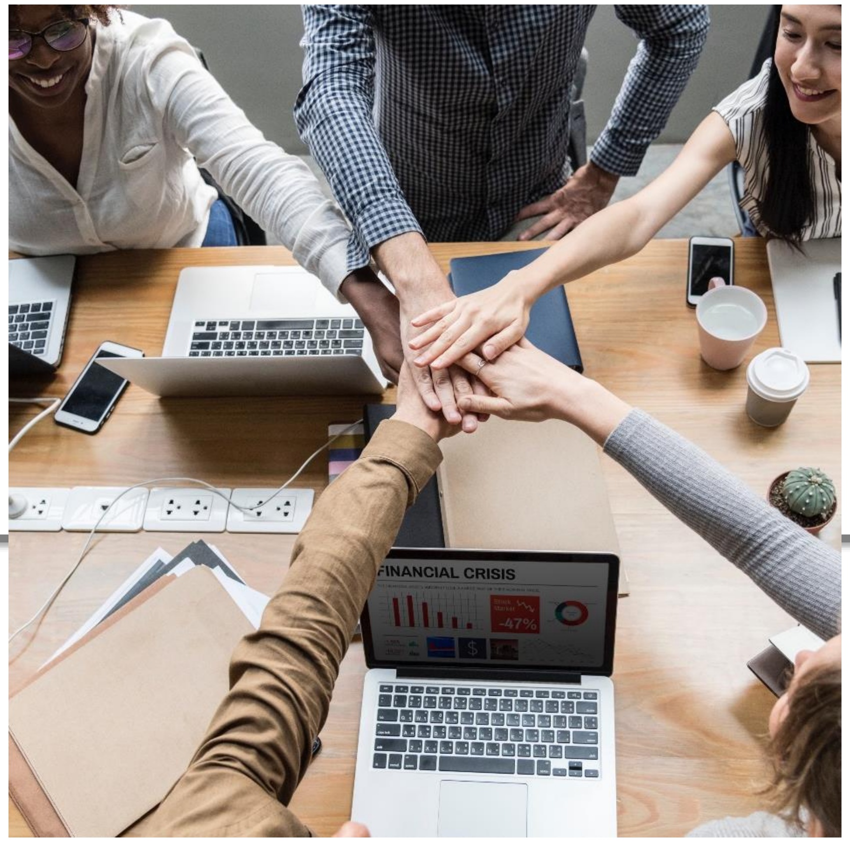

Connect

Why Peer Support is Important to Healthcare Workers

- Reduces Burnout
- Improves Mental Health
- Decreases Stigma
- Fosters a sense of Community

Adverse Patient Event or Outcome

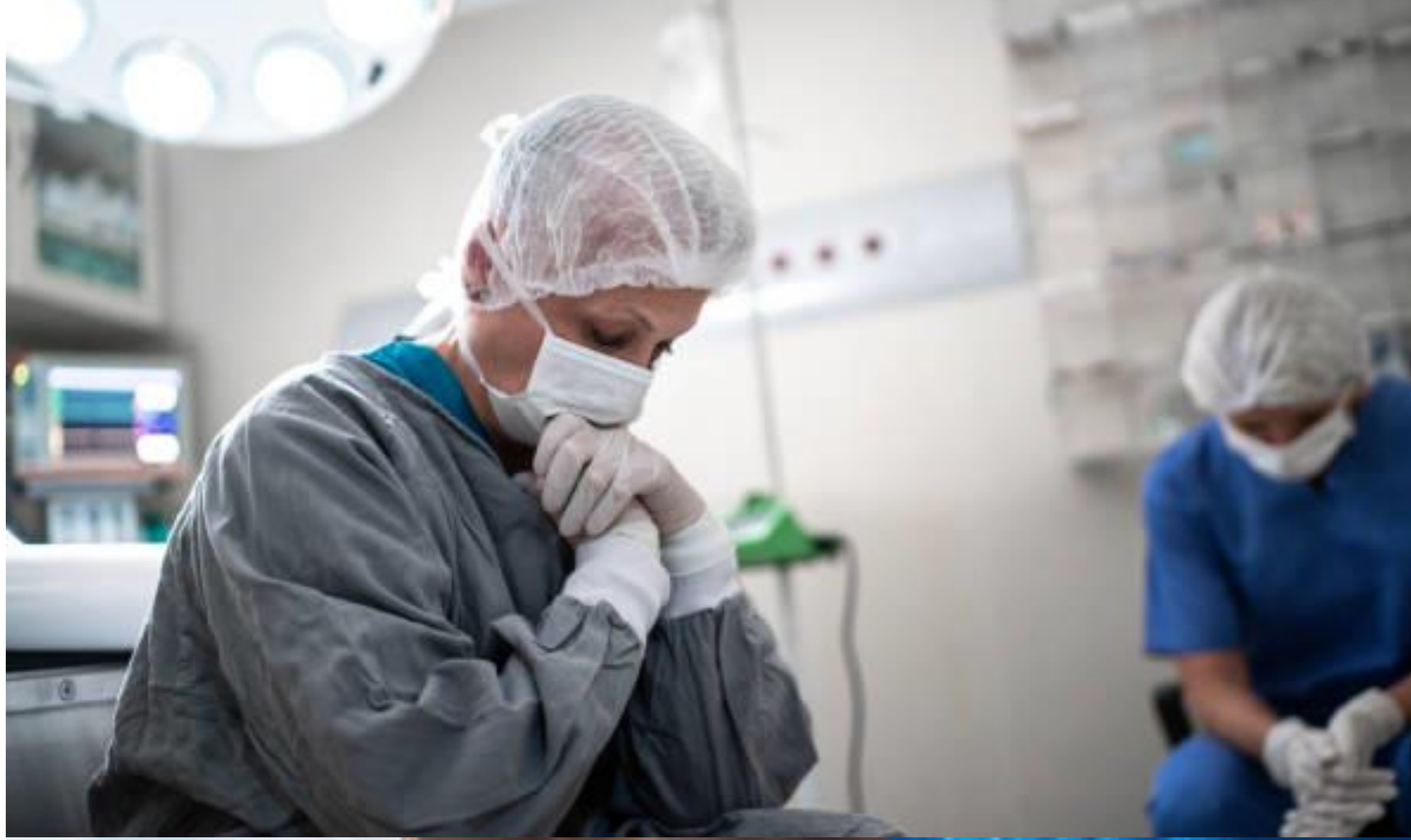

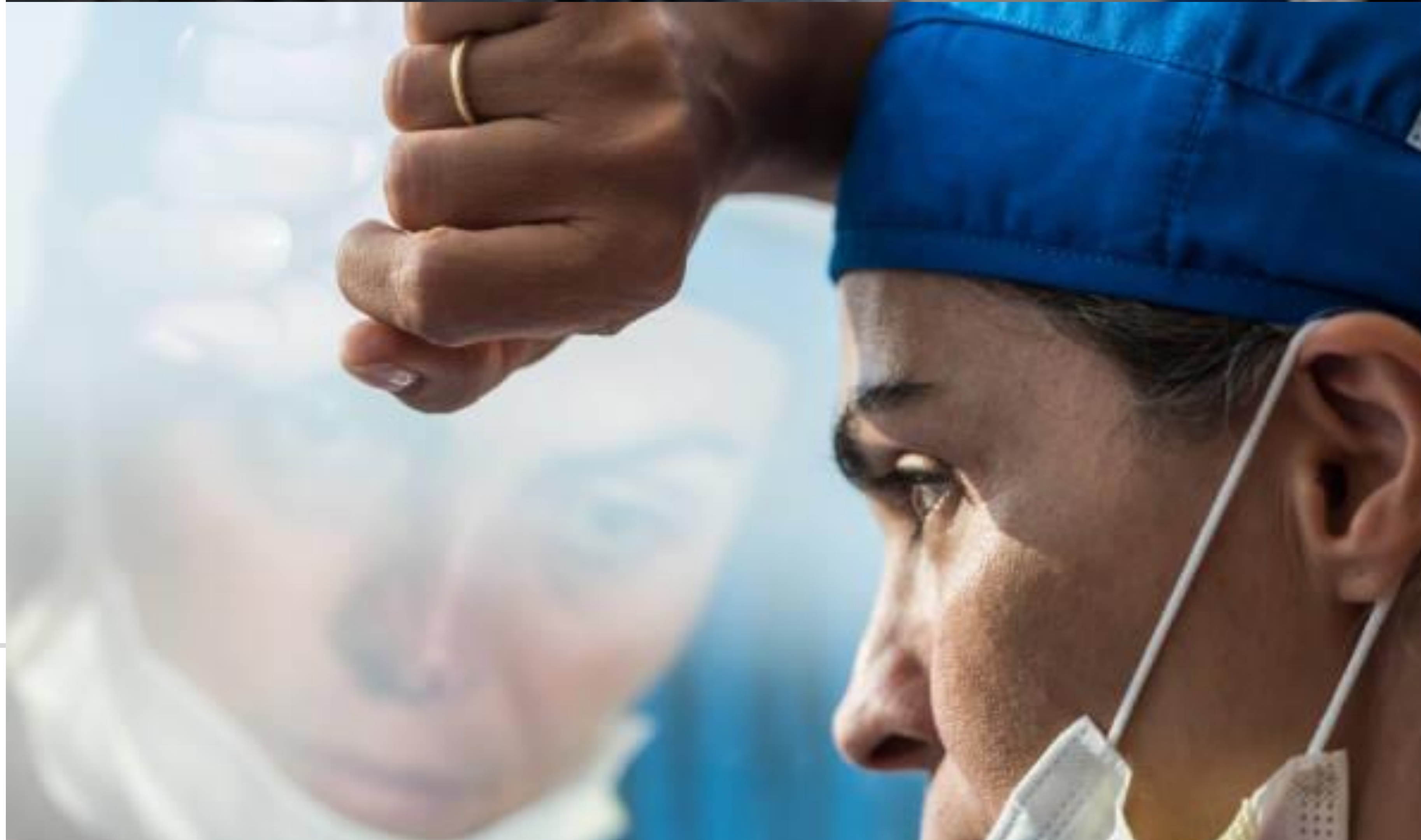

SECOND VICTIM

Medical error: the second victim

*"Virtually **every** practitioner knows the sickening realization of making a **bad** mistake. You feel singled out and exposed... You **agonize** about what to do... Later, the event **replays** itself over and **over** in your mind."*

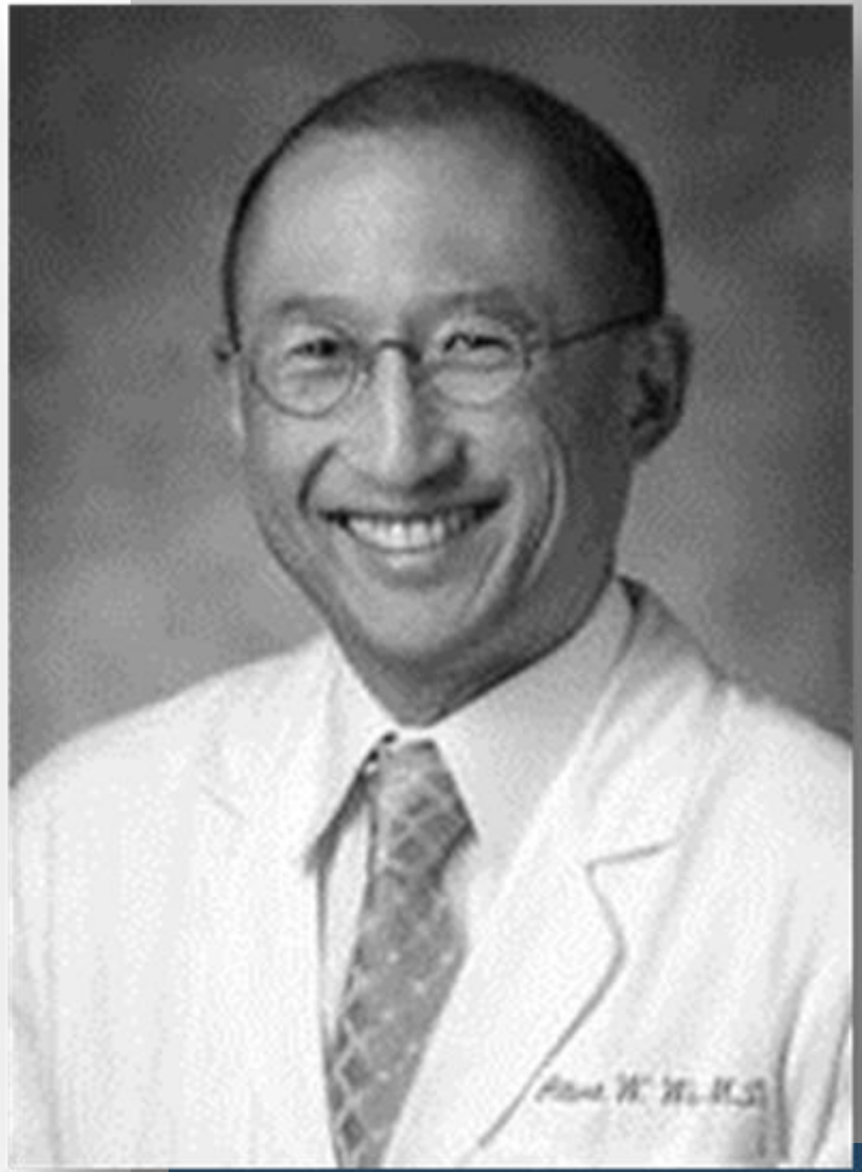

Albert Wu, MD

Second Victim Encounter Examples

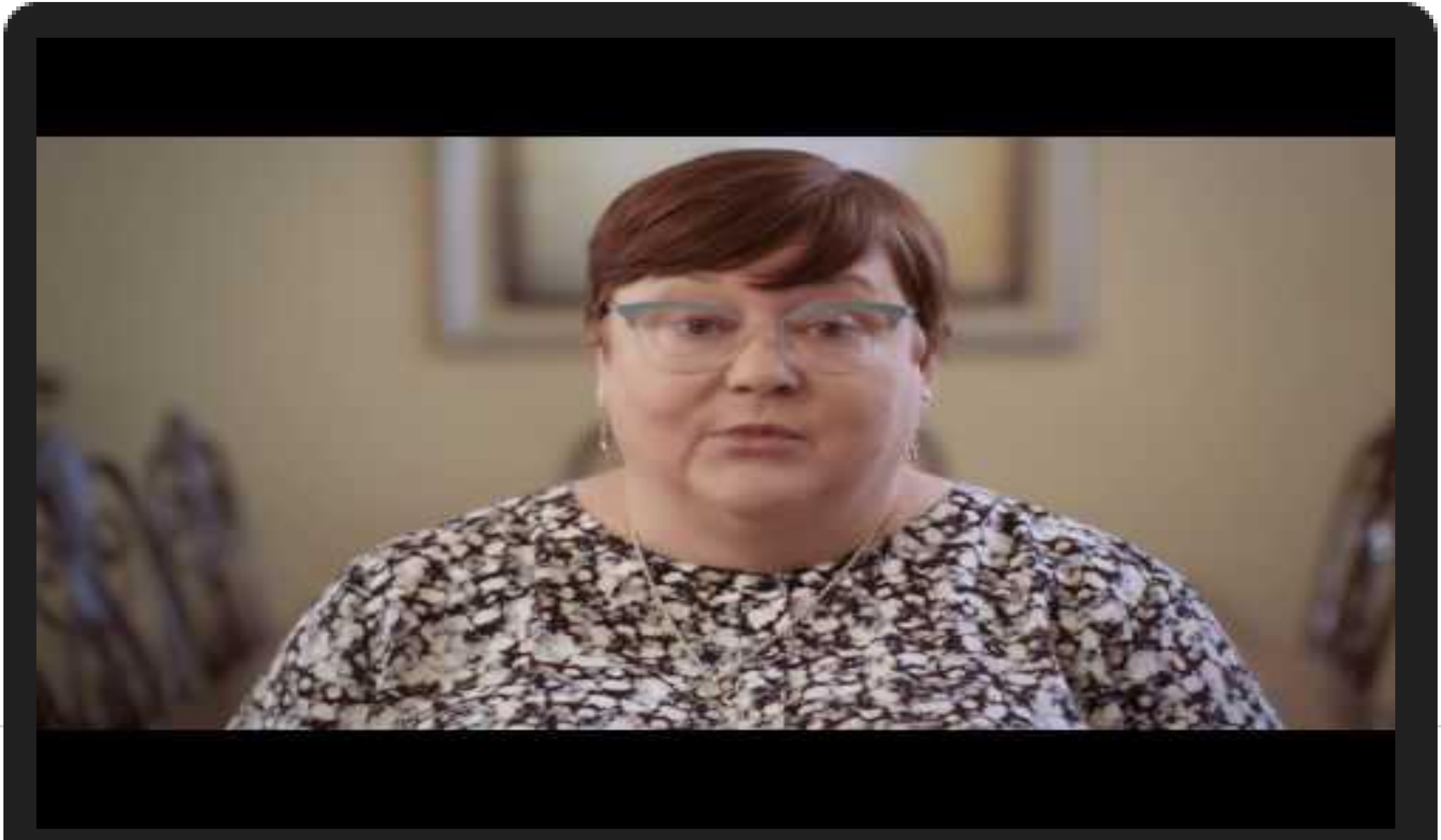

Group Debrief

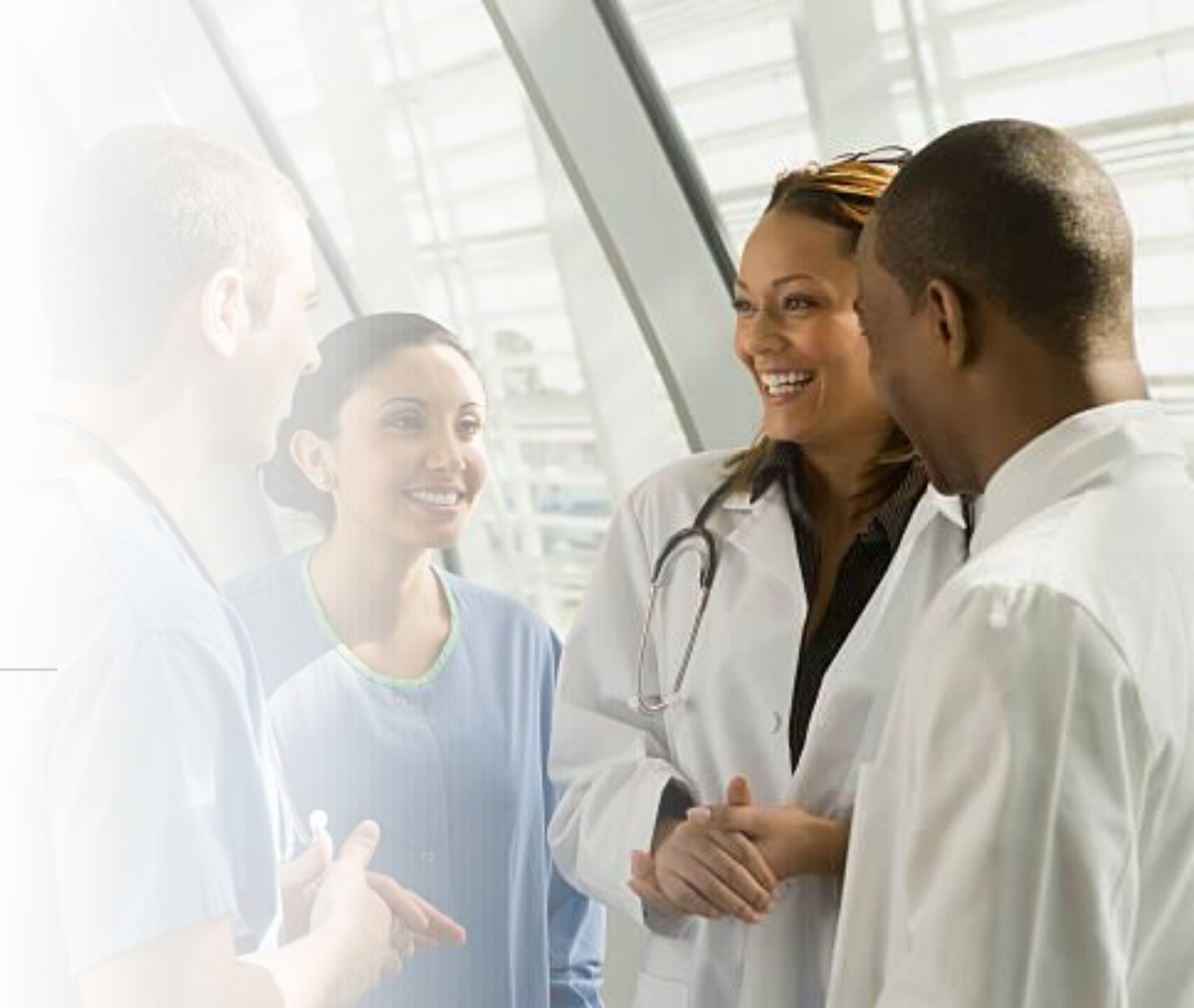

Commonly Heard Phrases

This event shook me to my core...

It just keeps playing over & over in my head

I do not deserve to practice medicine anymore...

This has been a turning point in my career..

People are going to start questioning my skills...

It's never been the same

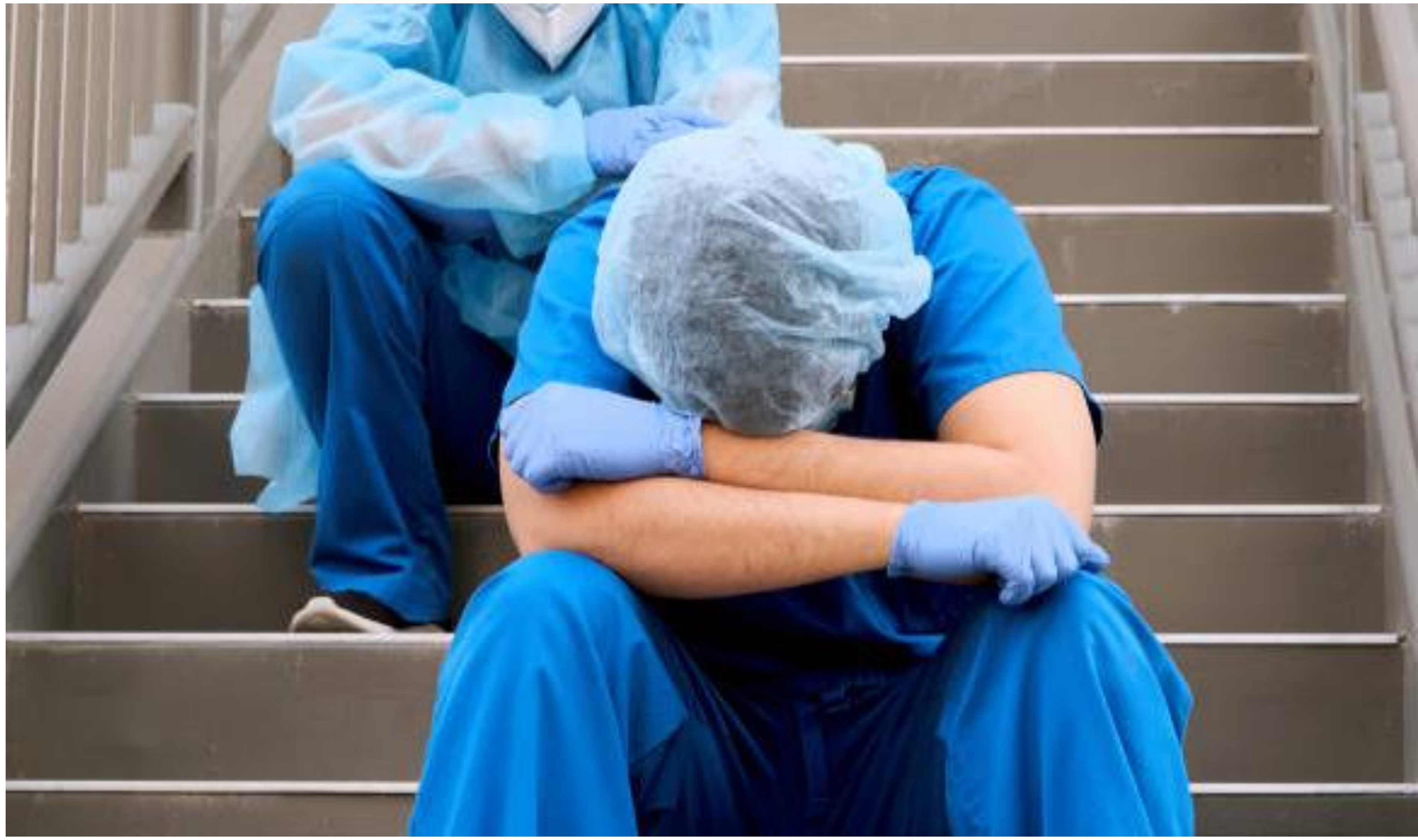

Common Stress Reaction

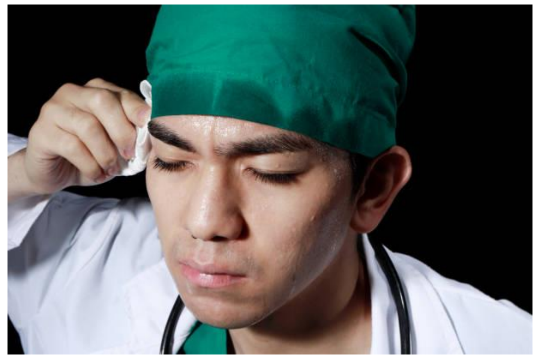

Sweating

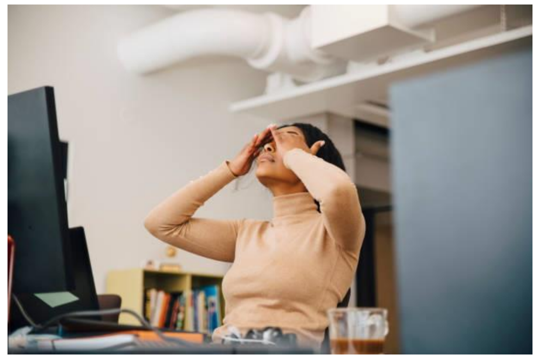

Poor Concentration

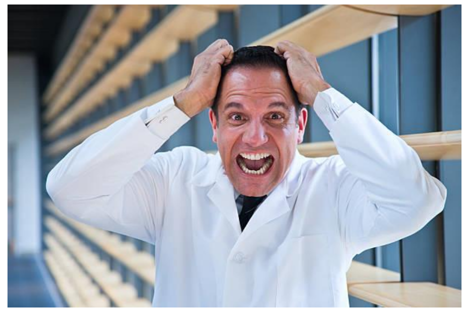

Yelling/
Irritability

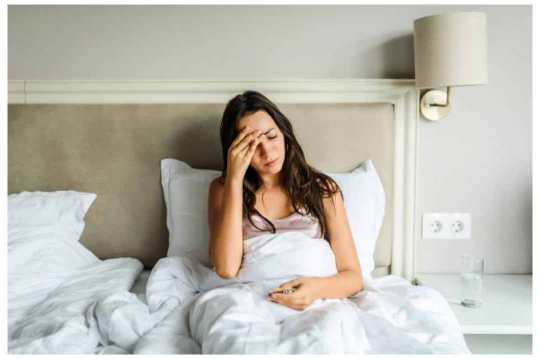

Fatigue

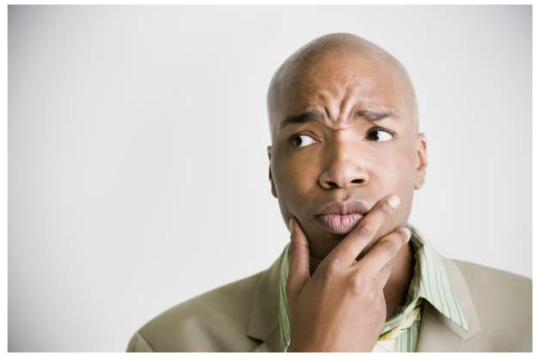

Inability to
Recall Events

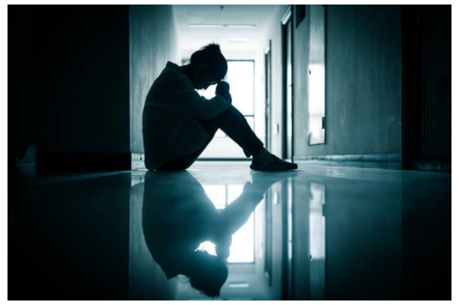

Isolation

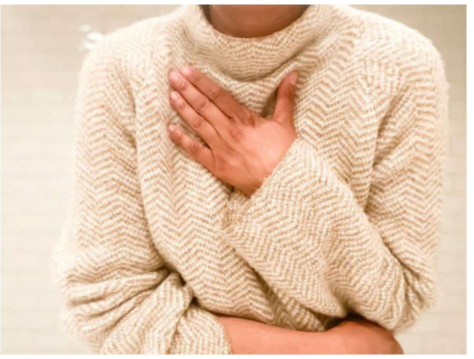

Rapid
Heartbeat

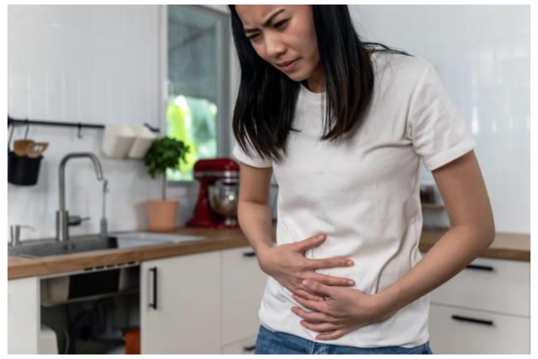

Physical
Body
Symptoms

Self-Reflection

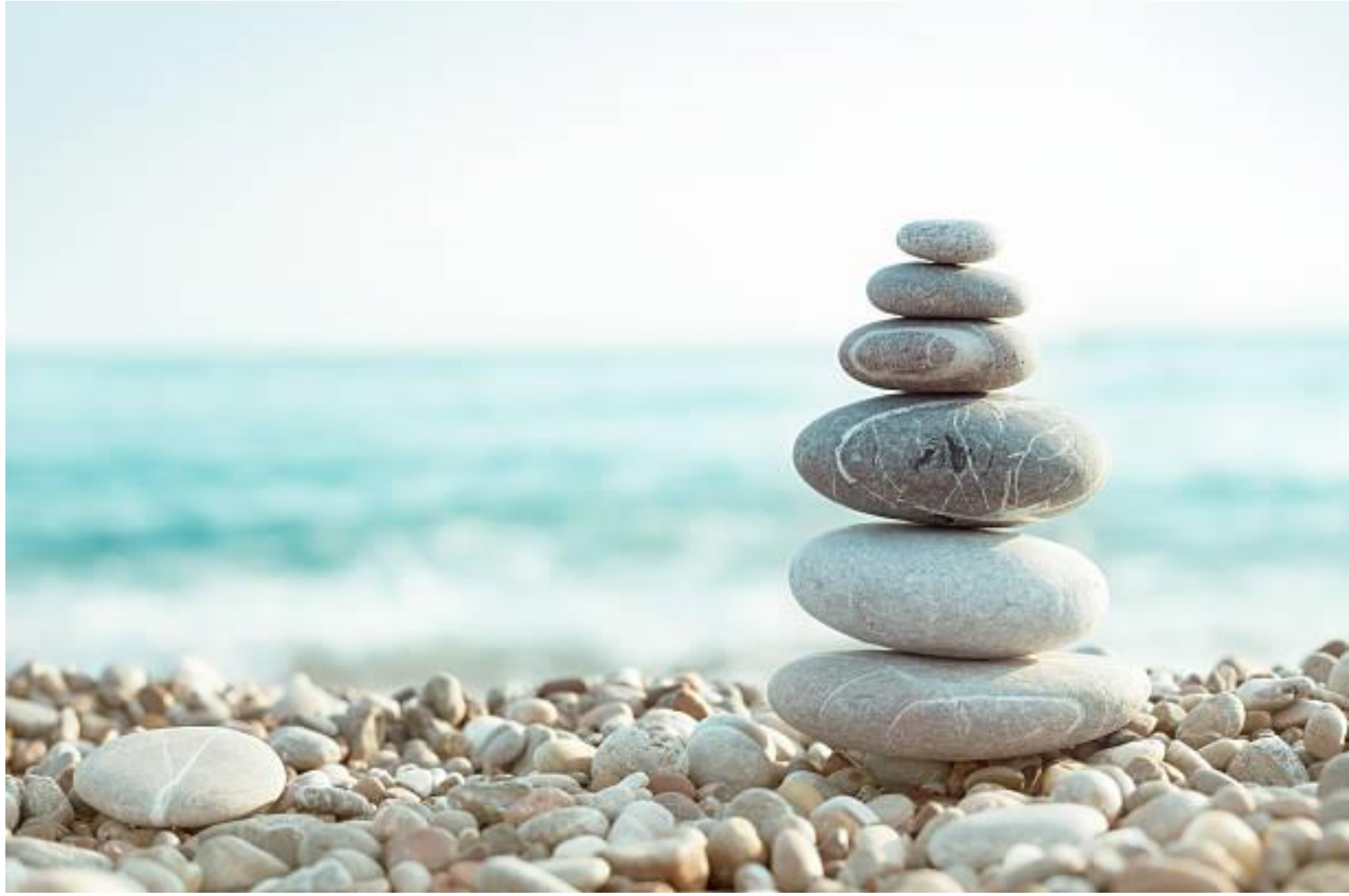

Second Victim Physical/Psychosomatic Symptoms

Medical error: the second victim

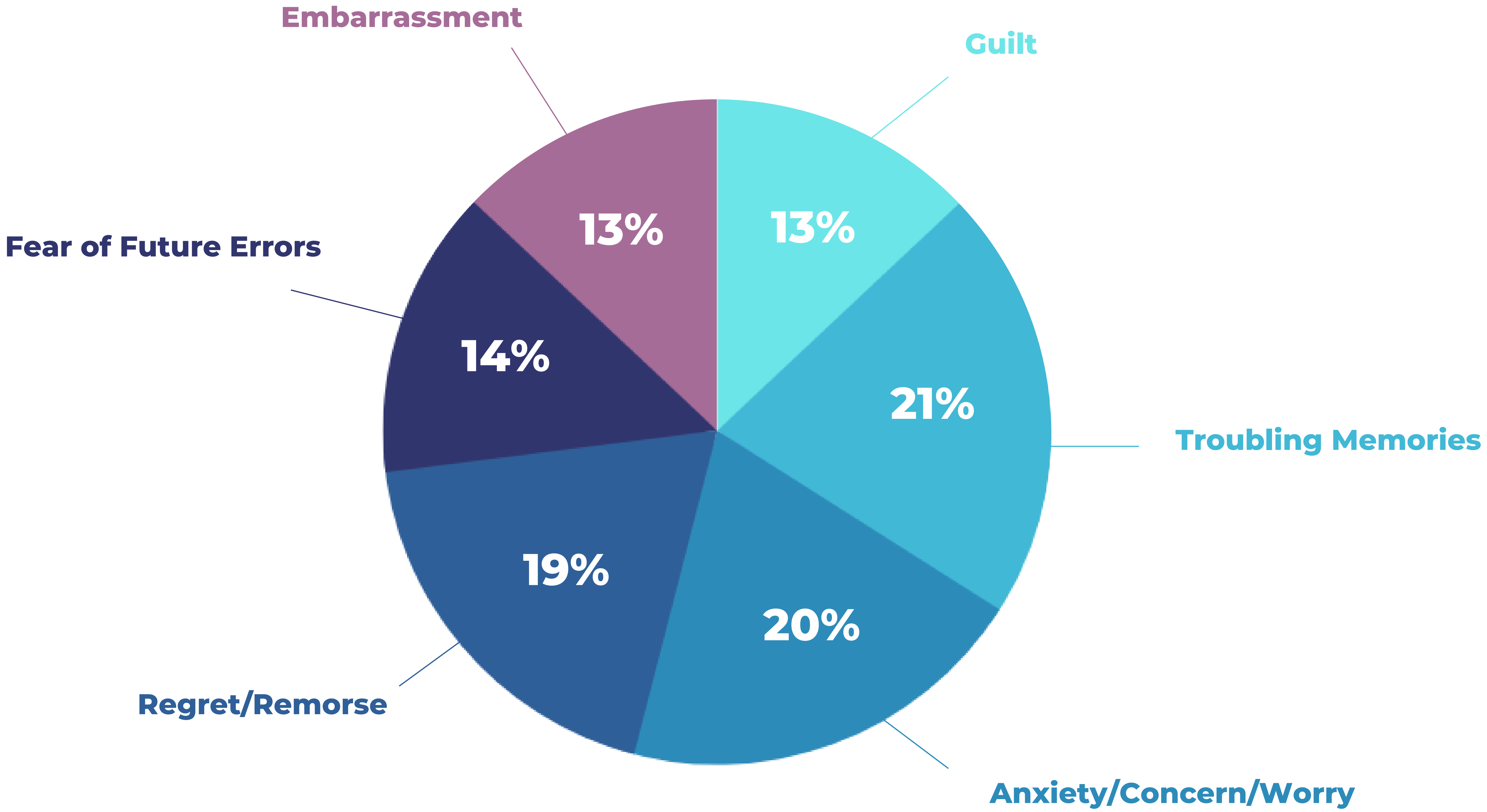

Busch, IM, Moretti, F, Purgato, M, Barbui, C., Wu, A. & Rimondini, M. (201bv 9). Journal of Patient Safety. Psychological and psychosomatic symptoms of second victims of adverse events: a systematic review and meta-analysis. Doi: 10.1097/PTS.0000000000000589

High Risk Scenarios

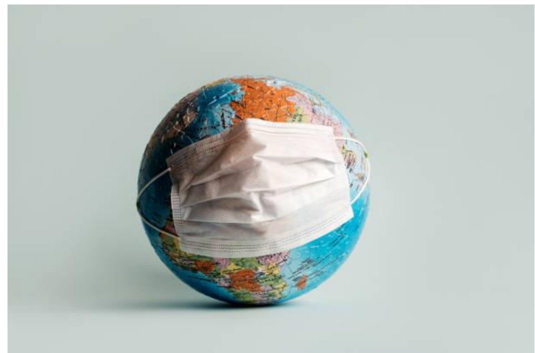

Global Pandemic

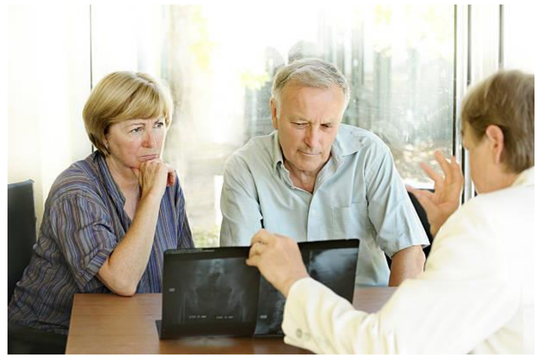

Poor Patient Outcomes

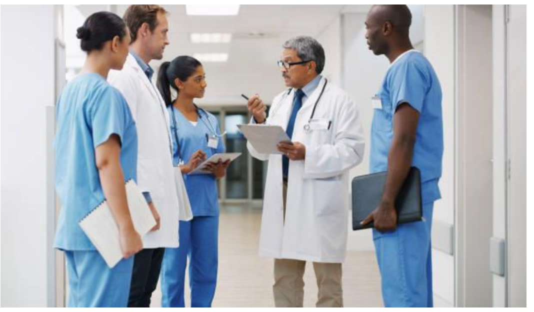

Internal Dynamics

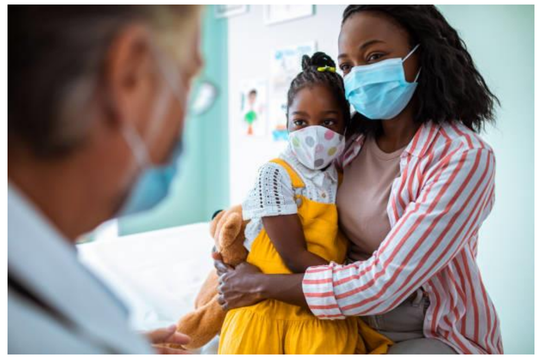

Pediatric cases

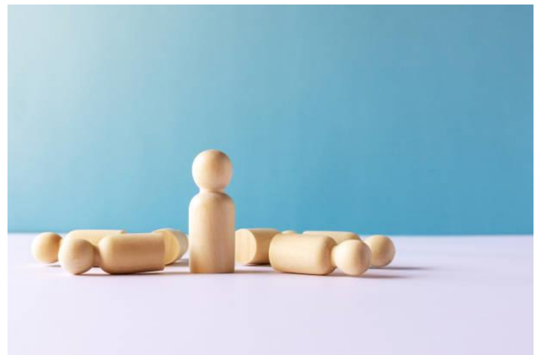

Failure to rescue cases

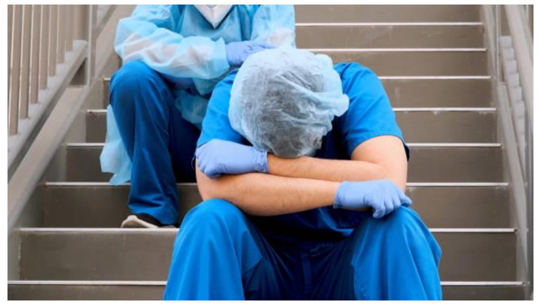

First Death Experience

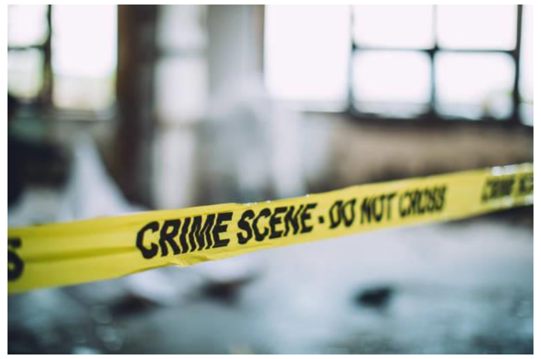

Workplace Violence Events

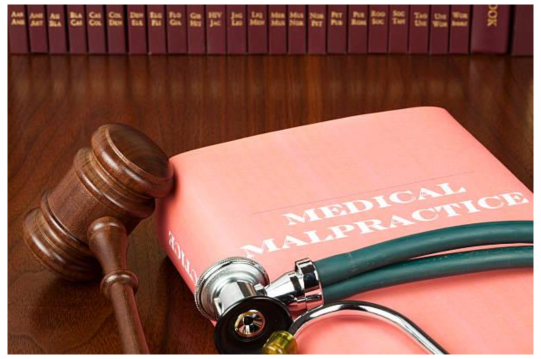

Medical Errors

Barriers to Receiving Support

- Stigma associated with reaching out for help
- Organizational patient safety blame culture
- Survival mode and time of getting help
- Shame- Loss of professional integrity
  - Loss of licensure
  - Compromise of collegial relationships
  - Future legal woes
  - HIPAA, confidentiality Implications
  - Vulnerability

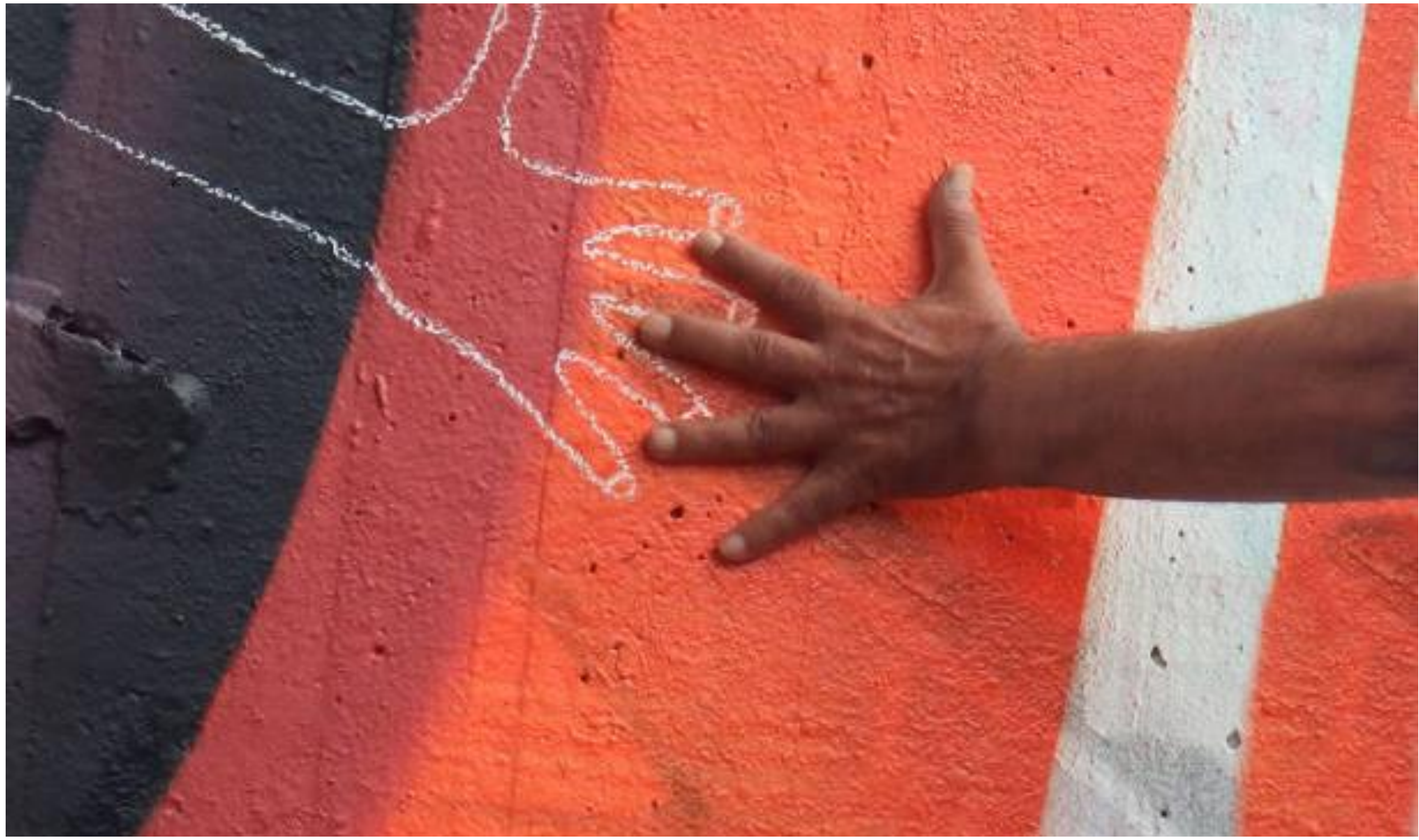

C.A.L.M. Peer Support Session

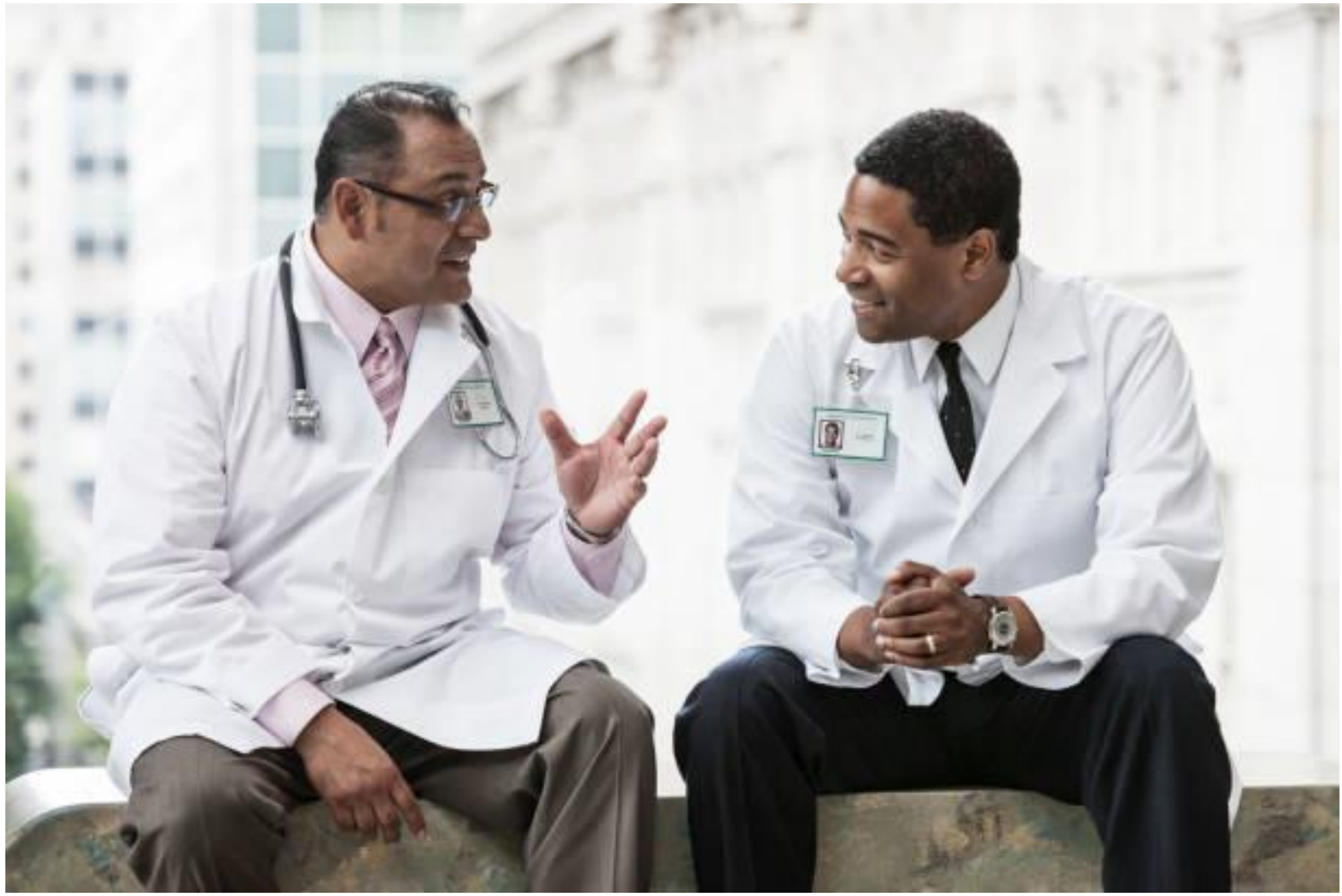

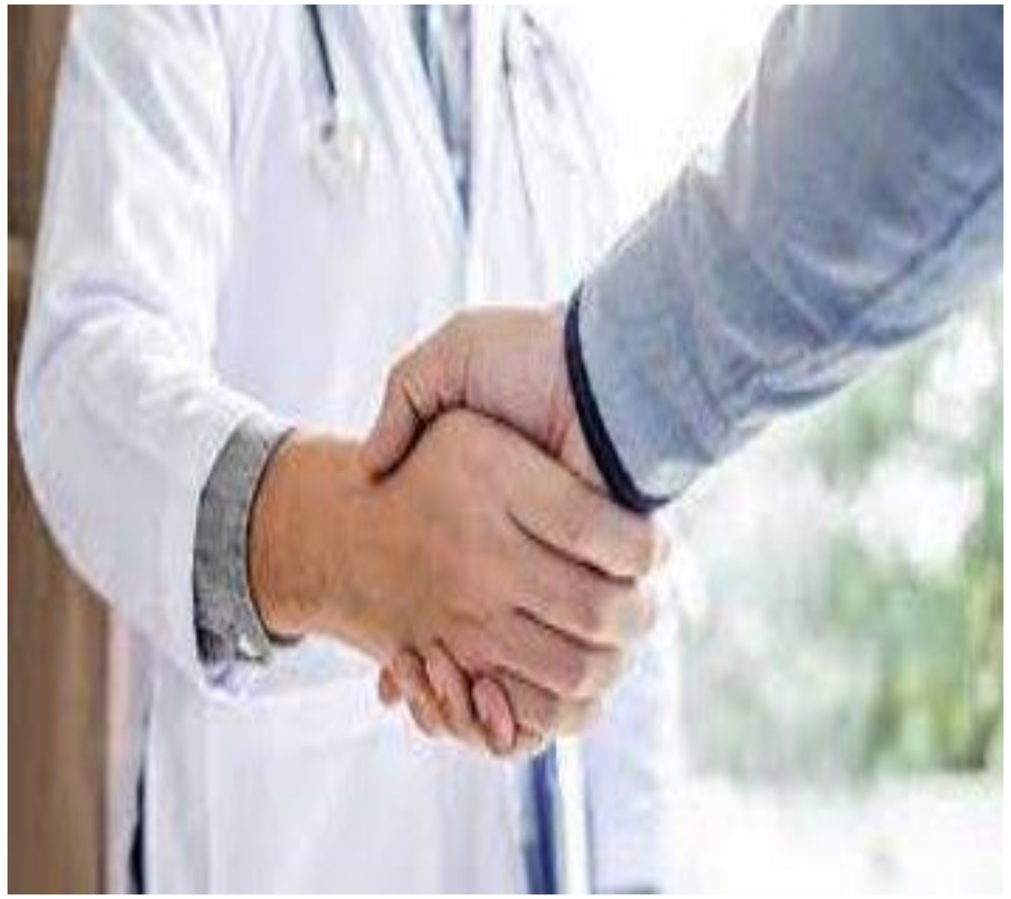

Introduction

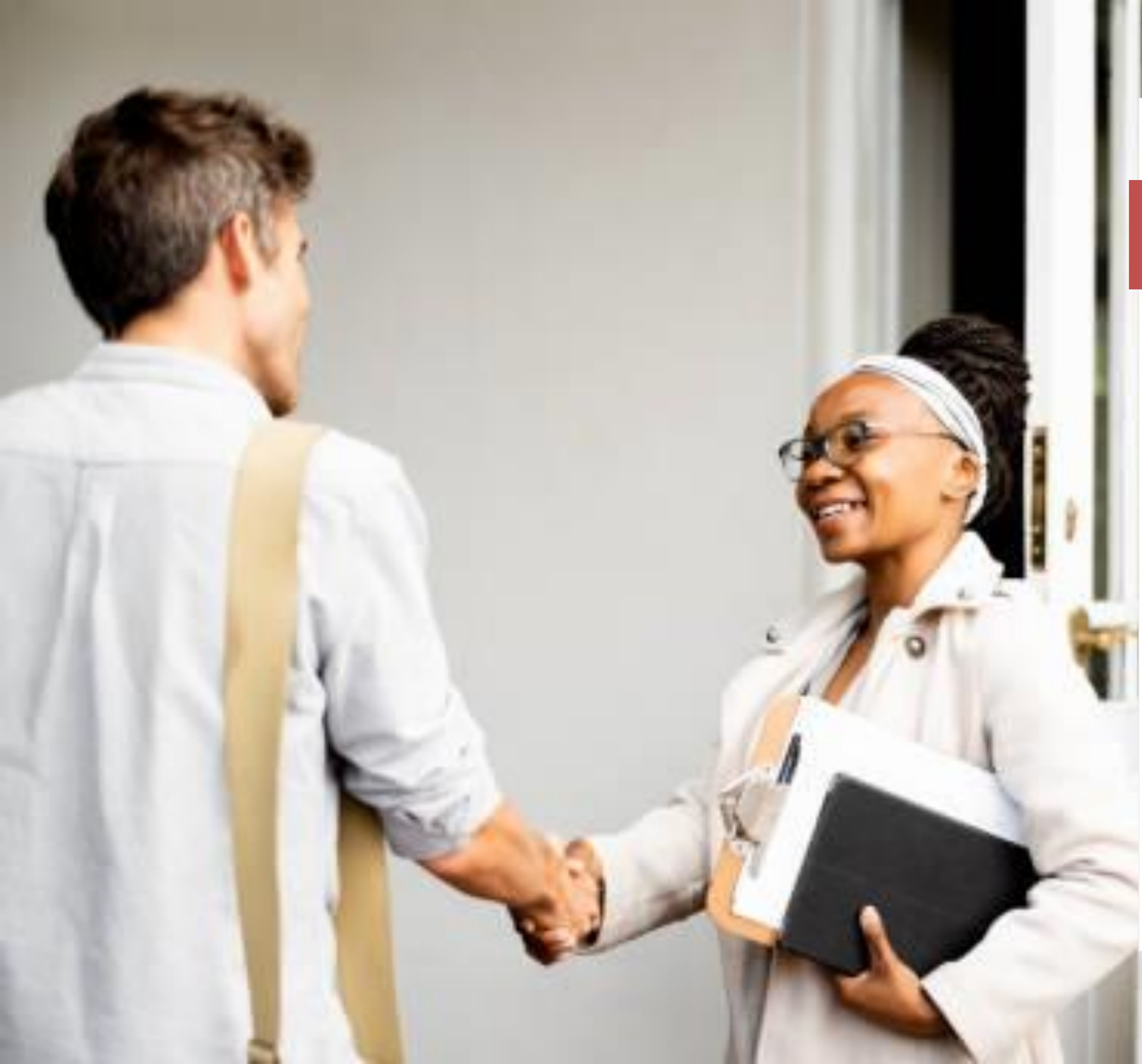

Introduction

- Introductions
- Build Rapport
- Establish Confidentiality

Introductions:

- Names & pronunciations
- Role(s)

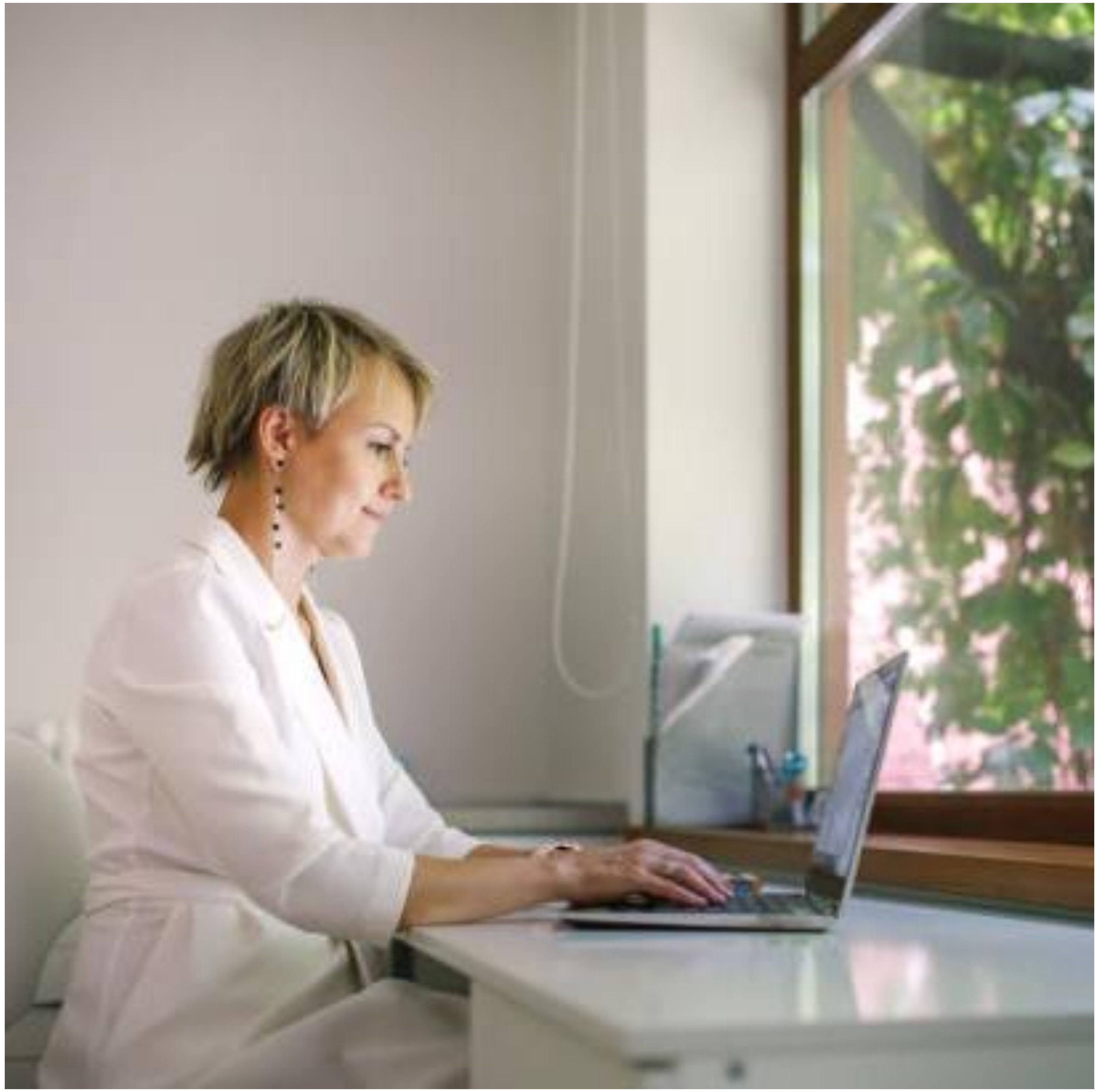

Build Rapport:

Small talk before big talk
- Weather
- Commute
- Weekend
- Pace of day
- Office / Environment
- Accessories

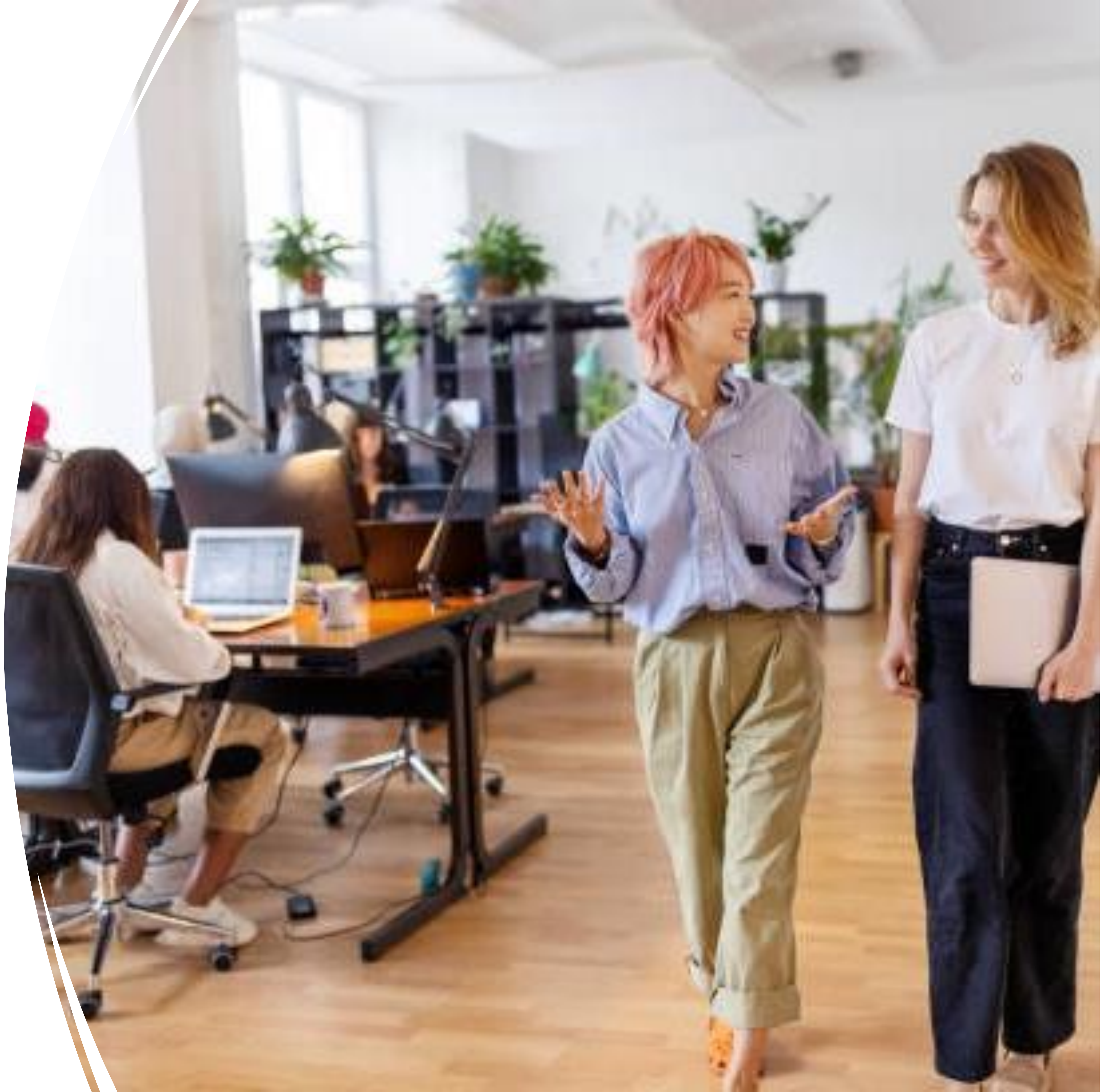

Scope of Peer Support:

- My role in peer support is to:
  - Listen
  - Offer understanding and support
  - Explore:
    - Your experience
    - Coping strategies
    - Resources

- "I am not a therapist or counselor."
- "I am here to provide a supportive and compassionate space for you."

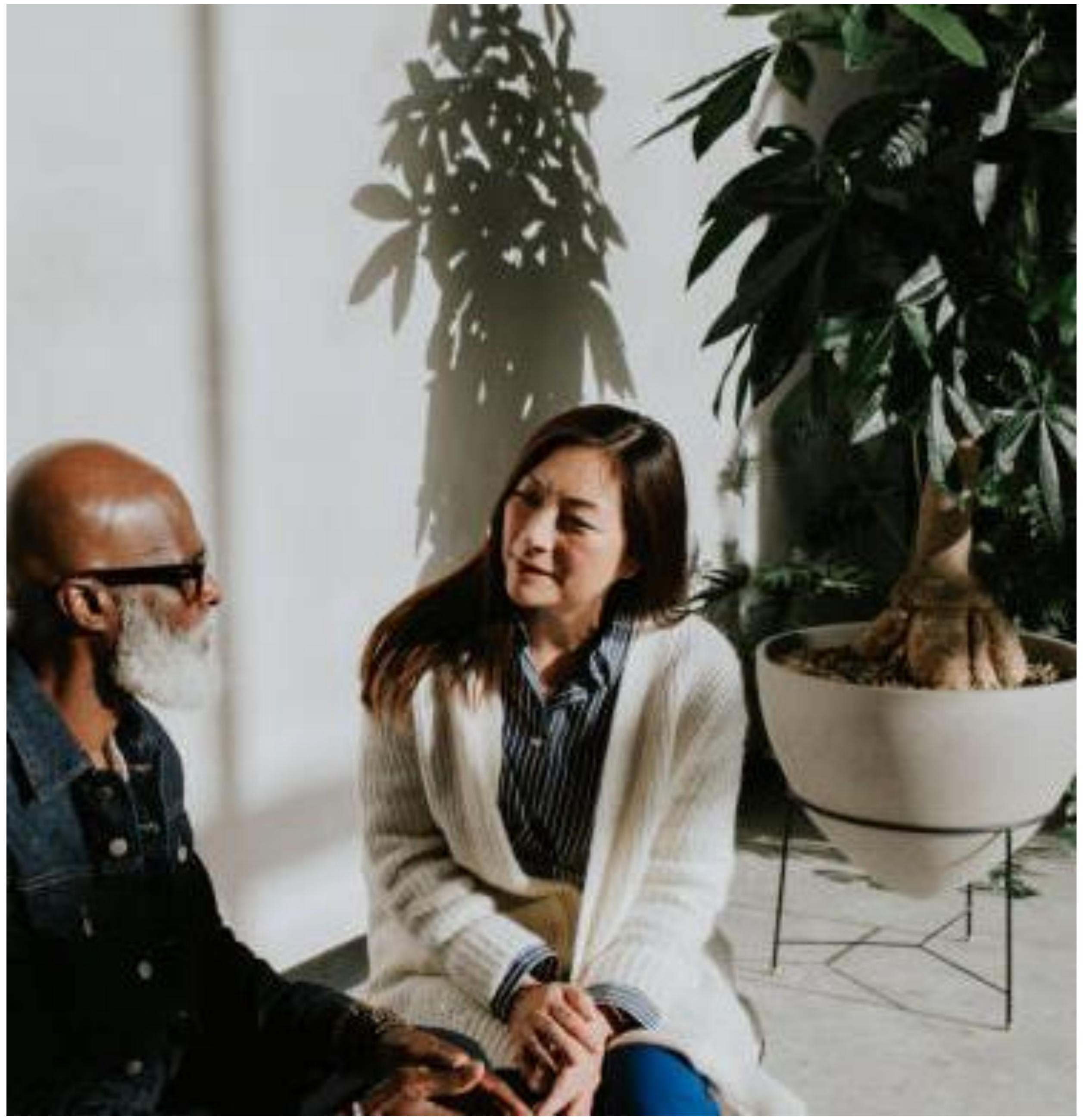

Confidentiality:

- Transparency about exceptions:
  - Intent to harm self/others
  - Title IX- discrimination on the basis of sex
  - Sexual harassment, sexual assault, dating violence, domestic violence, stalking, or other sexual misconduct committed by or against a student, resident, faculty, or employee
  - Substance use
- "What questions or concerns do you have?"

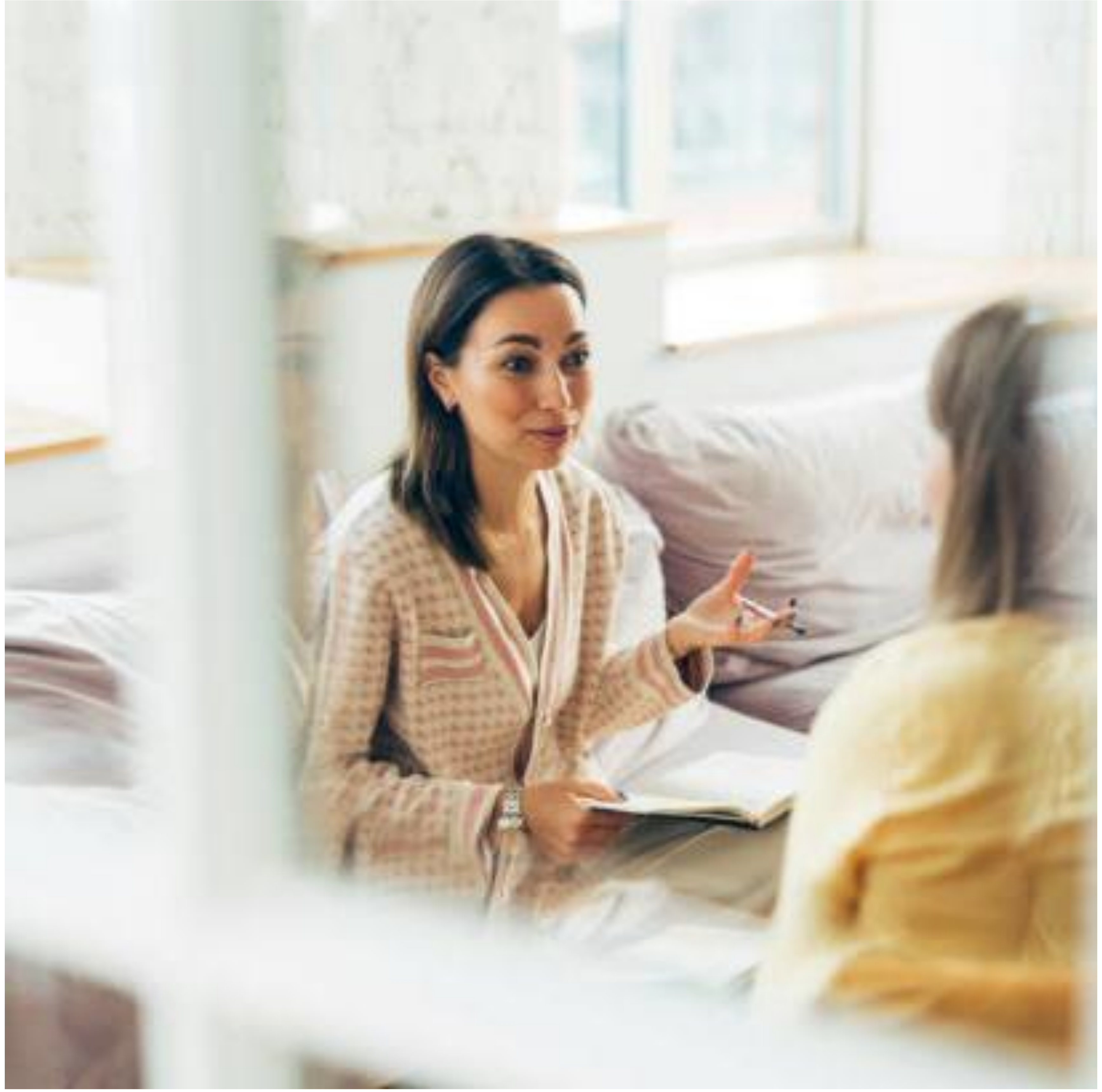

Demo & Skills Practice

- Introductions
- Build Rapport
- Establish Confidentiality

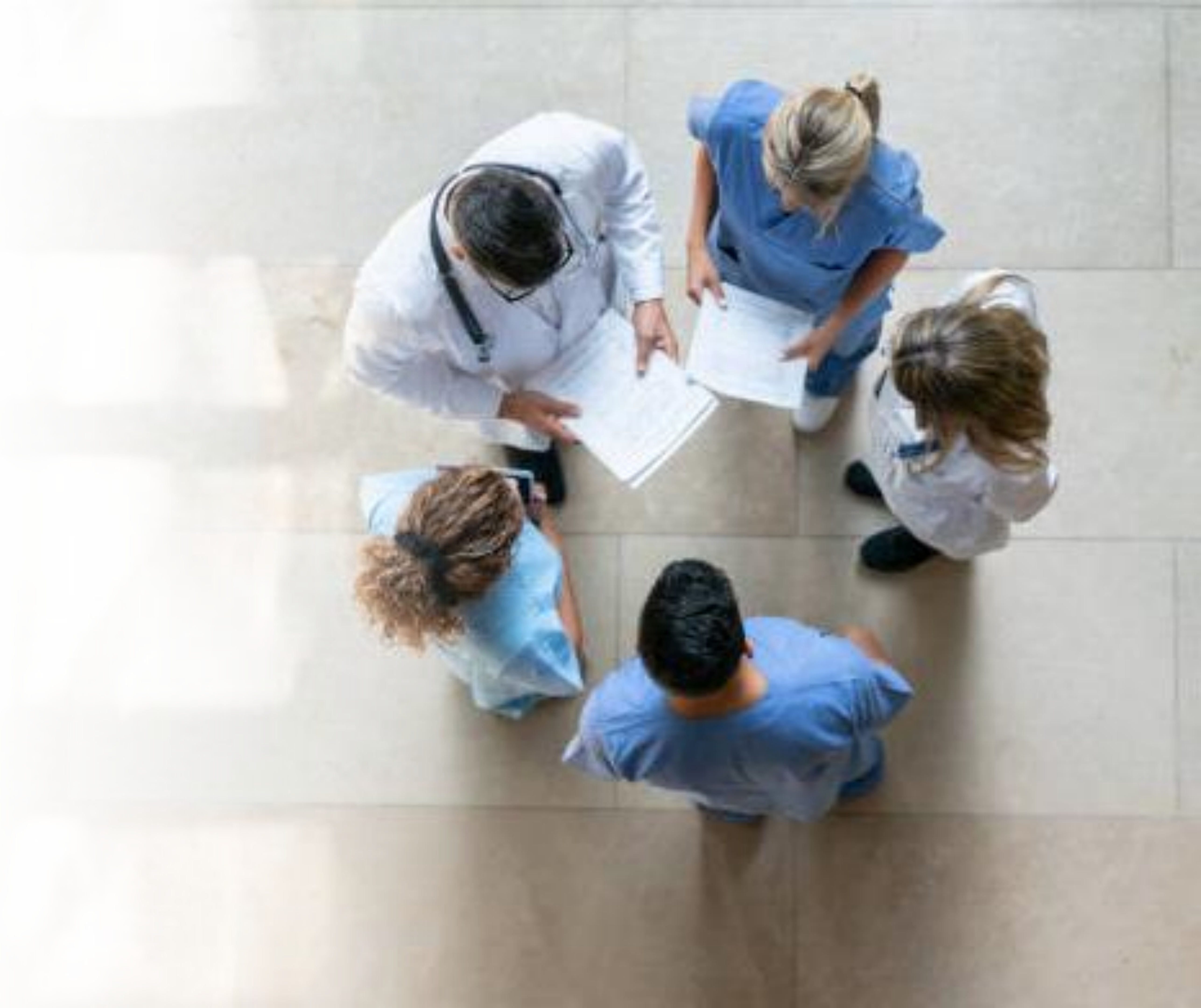

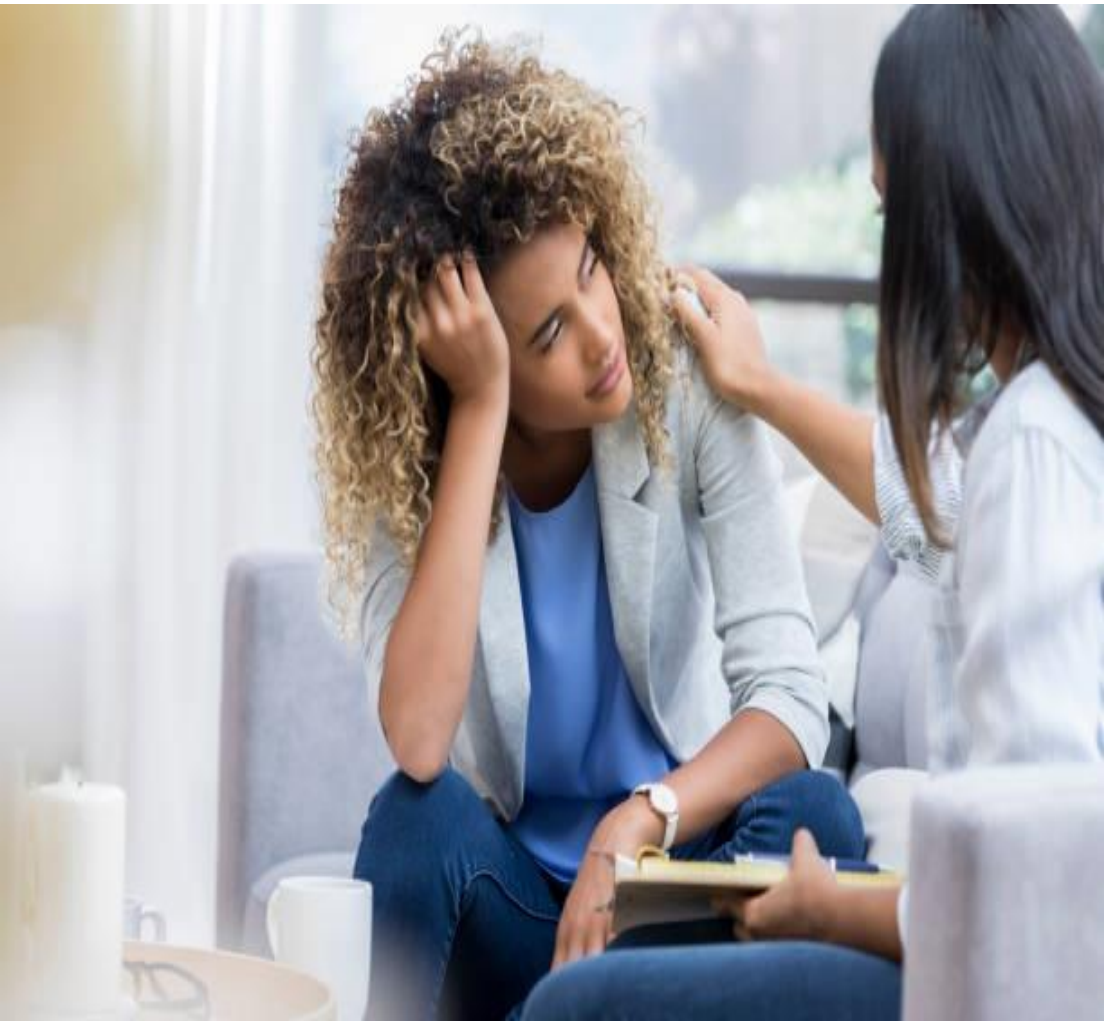

Exploration of Emotions

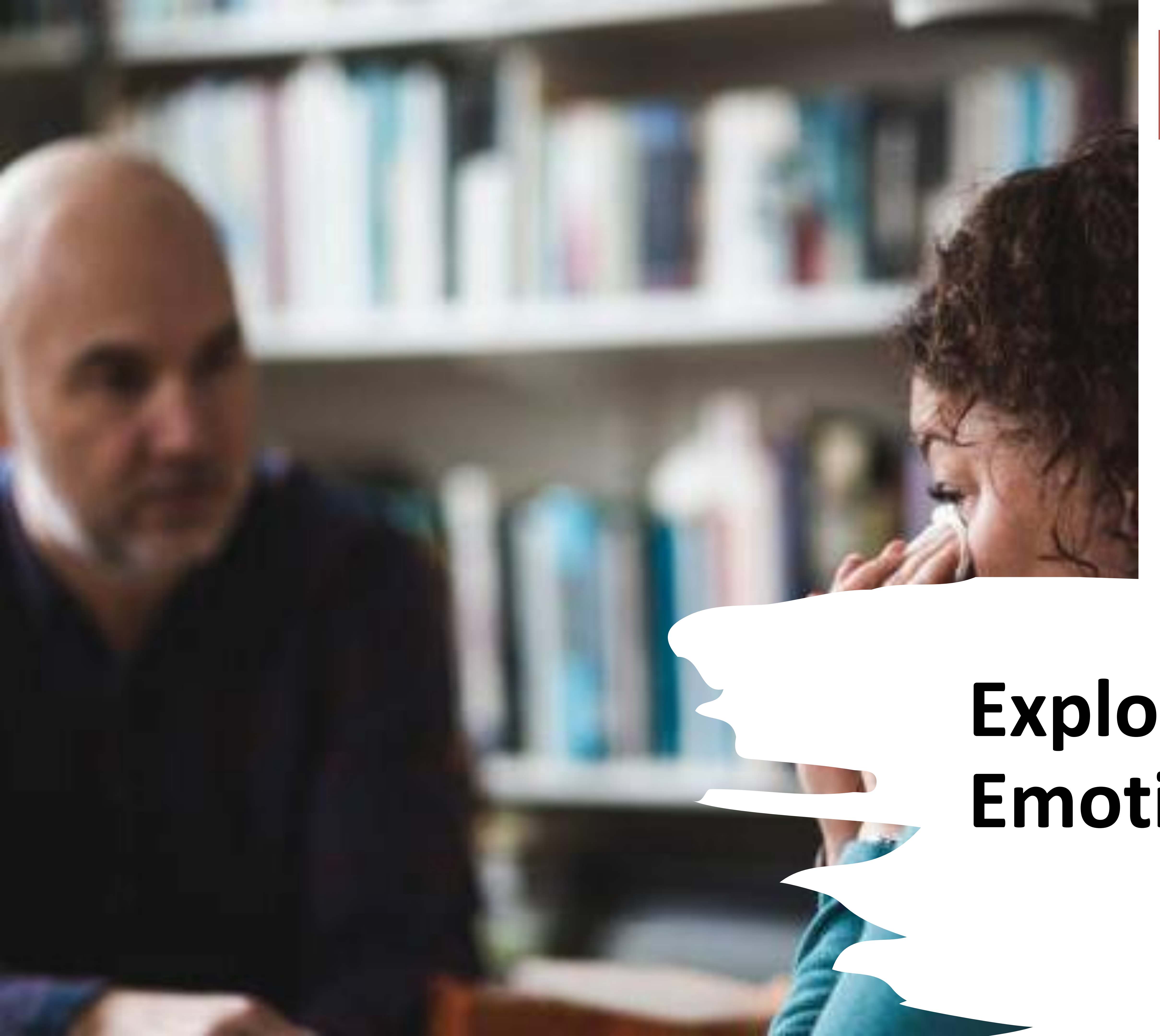

Exploration of Emotions

- Open-Ended Questions
- Respond with Compassion
- Avoid moving too quickly to solutions

Exploration of Emotions

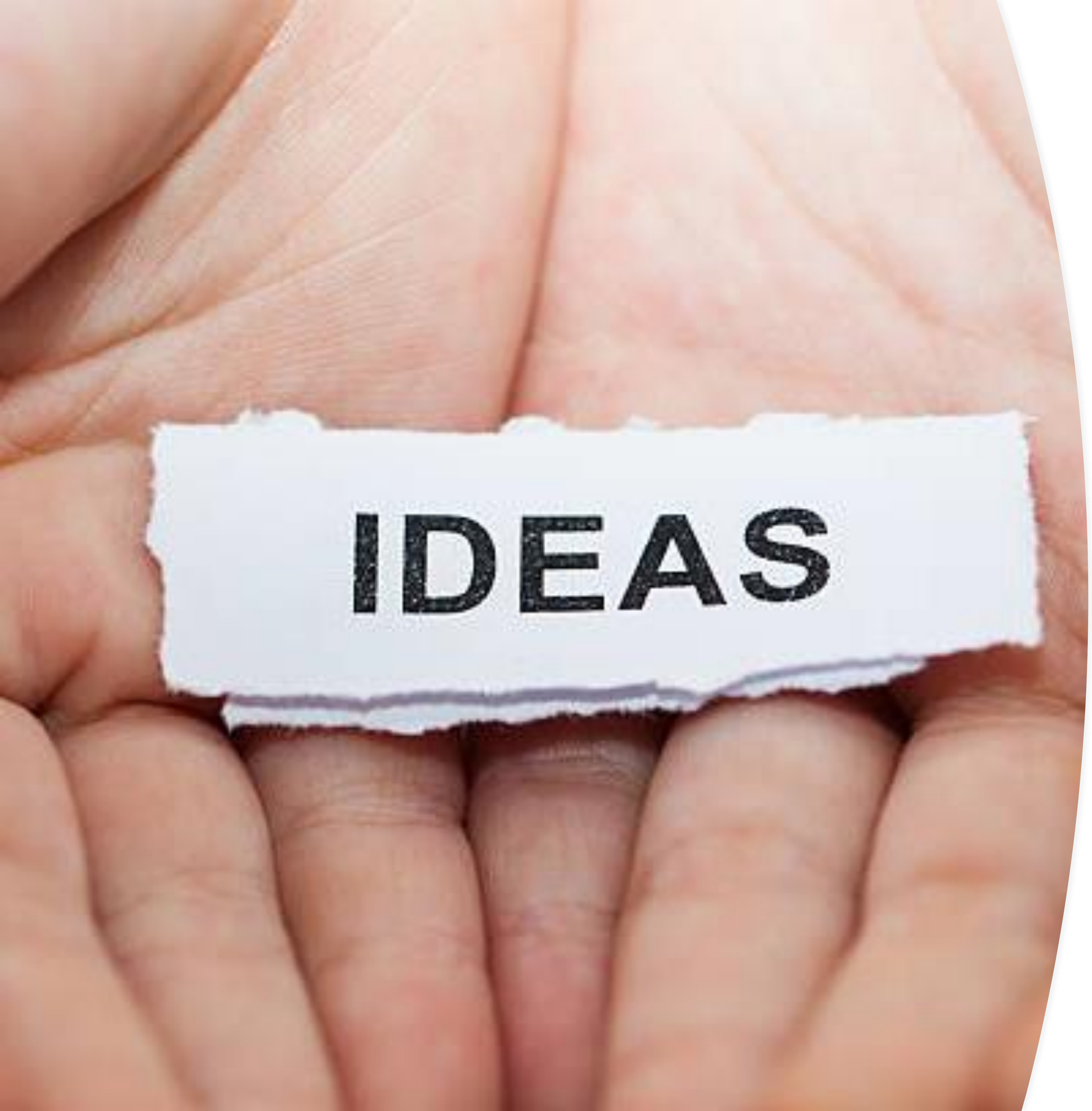

Open-Ended Questions:

- How are you doing with all of this?
- How is this affecting you?
- What is hurting most right now?
- How can I support you right now?
- What do you need right now?

Exploration of Emotions
Respond with empathic PEARLS©

Academy of Communication in Healthcare

- **P**artnership *"We are in this together as colleagues…"*
- **E**motion *"I can hear how much frustration this case has caused you…"*
- **A**pology *"I acknowledge the team may have not supported you in the way you would have hoped."*
- **R**espect *"It takes great courage to talk about this."*
- **L**egitimization *"It is natural to have these feelings and emotions following a complex case."*
- **S**upport *" I am here to support you as you process these feelings. What part of this are you struggling with right now?"*

Flickinger, et al. *Patient Educ Couns*. 2016. Hagiwara, et al. *Patient Educ Couns*. 2019.Kim, Kaplowitz, and Johnston. *Eval Health Prof.* 2004

Exploration of Emotions
Respond with empathic PEARLS©

- **P**artnership
- **E**motion
- **A**pology
- **R**espect
- **L**egitimization
- **S**upport

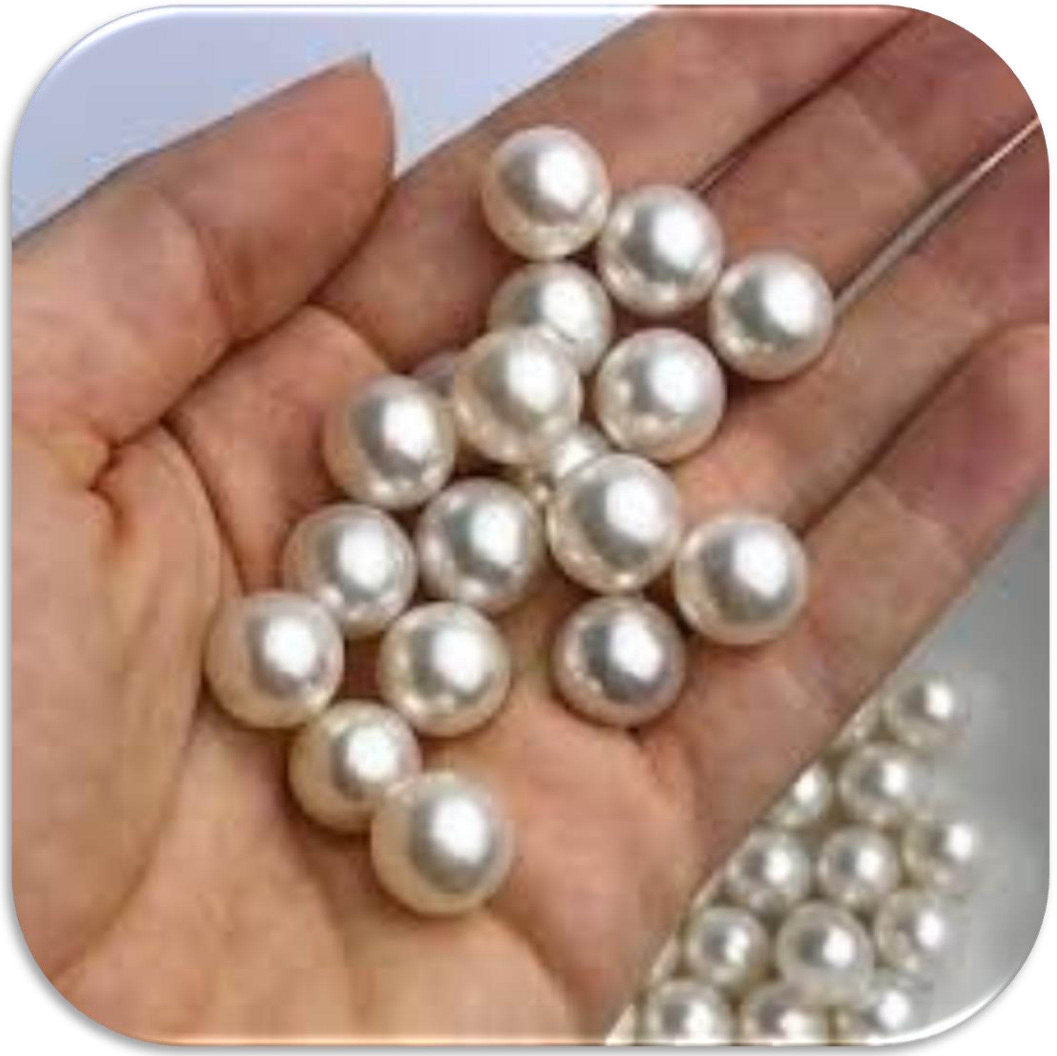

Flickinger, et al. *Patient Educ Couns*. 2016. Hagiwara, et al. *Patient Educ Couns*. 2019.Kim, Kaplowitz, and Johnston. *Eval Health Prof.* 2004

Exploration of Emotions

Respond with empathic PEARLS©

Academy of Communication in Healthcare

- **P**artnership *"We are in this together as colleagues…"*

Peer: "I feel so alone in all of this and the shame I feel doesn't allow me to talk to anyone about my feelings."
Peer Supporter: "Shame can feel very heavy and can be a lot to carry alone. As your peer supporter, I will be here to support you. We are in this together."

Flickinger, et al. *Patient Educ Couns*. 2016. Hagiwara, et al. *Patient Educ Couns*. 2019.Kim, Kaplowitz, and Johnston. *Eval Health Prof.* 2004

Exploration of Emotions
Respond with empathic PEARLS©

Academy of Communication in Healthcare

- **E**motion *"I can hear how much frustration this case has caused you…"*

Peer: "I'm just so frustrated with myself. I keep making the same mistakes over and over again."
Peer Supporter: "It's completely understandable to feel frustrated. Making mistakes can be disheartening, and we all make them as humans."

Flickinger, et al. *Patient Educ Couns*. 2016. Hagiwara, et al. *Patient Educ Couns*. 2019.Kim, Kaplowitz, and Johnston. *Eval Health Prof.* 2004

Exploration of Emotions
Respond with empathic PEARLS©

Academy of Communication in Healthcare

- **A**pology *"I acknowledge the team may have not supported you in the way you would have hoped."*

Peer: "I just feel like the team isn't supporting me. This was my first time making a medical error and now everyone is treating me as if I know nothing about medicine. I feel like an outcast..."
Peer Supporter: "I am sorry that you are not receiving the support from your team like you would have hoped. What support do you need most right now?"

Flickinger, et al. *Patient Educ Couns*. 2016. Hagiwara, et al. *Patient Educ Couns*. 2019.Kim, Kaplowitz, and Johnston. *Eval Health Prof.* 2004

Exploration of Emotions
Respond with empathic PEARLS©

Academy of Communication in Healthcare

- **R**espect *"It takes great courage to talk about this."*

Peer: "It is so hard to find someone to talk to who truly understands this part of medicine, the constant struggles patient care, feeling inadequate, and really no longer wanting to practice medicine anymore. I always think, why am I even doing this job."
Peer Supporter: "It takes a lot of courage to seek out support to express things feelings and that's admirable. It also takes a lot of courage to practice medicine and care for other people."

Flickinger, et al. *Patient Educ Couns*. 2016. Hagiwara, et al. *Patient Educ Couns*. 2019.Kim, Kaplowitz, and Johnston. *Eval Health Prof.* 2004

Exploration of Emotions
Respond with empathic PEARLS©

Academy of Communication in Healthcare

- **L**egitimization *"It is natural to have these feelings and emotions with competing priorities."*

Peer: "I've been having a hard time balancing my personal life and work. I feel guilty for not spending enough time with my family."
Peer Supporter: "I appreciate you opening up about this. It's common to struggle with work-life balance, and it can be challenging to find the right equilibrium. Remember, it's okay to prioritize your well-being and make adjustments that work for you and your family."

Flickinger, et al. *Patient Educ Couns*. 2016. Hagiwara, et al. *Patient Educ Couns*. 2019.Kim, Kaplowitz, and Johnston. *Eval Health Prof.* 2004

Exploration of Emotions
Respond with empathic PEARLS©

Academy of Communication in Healthcare

- **S**upport *" I am here to support you as you process these feelings. What part of this are you struggling with right now?"*

  Peer: "I find myself continuously thinking about what will happen next, if I even want to be a healthcare provider anymore. Everything is just a lot right now, and I can't gather my thoughts and I feel alone."
  Peer Supporter: "Navigating those feelings by yourself can be overwhelming. Know that you are not alone, I am here to support you as you navigate those feelings."

Flickinger, et al. *Patient Educ Couns*. 2016. Hagiwara, et al. *Patient Educ Couns*. 2019.Kim, Kaplowitz, and Johnston. *Eval Health Prof.* 2004

What else would you want to share?

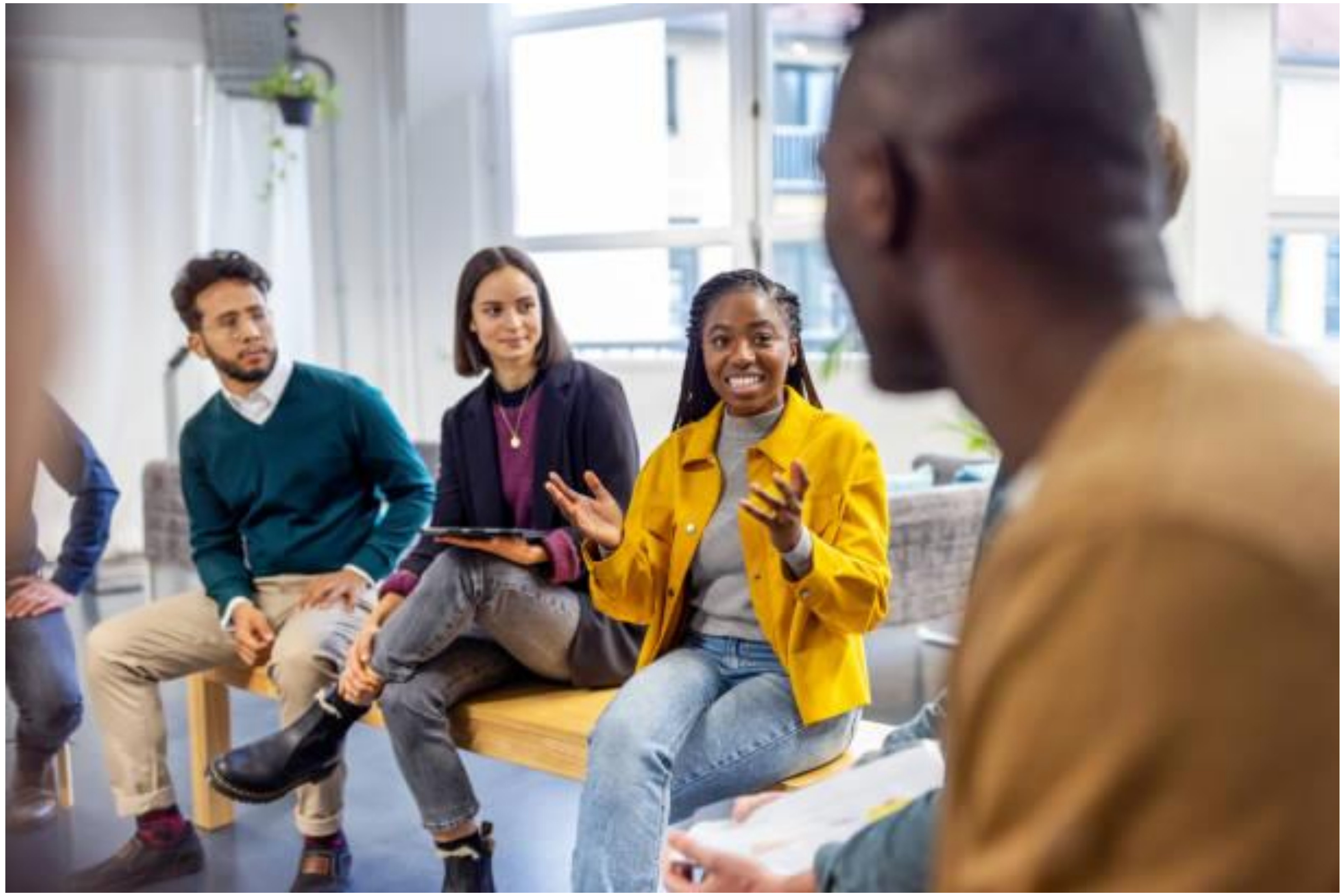

Demo & Skills Practice

Exploring Emotions:

- Open-Ended Questions
- Respond with PEARLS of compassion
- Resist moving too quickly to ideas/solutions!
- What else?

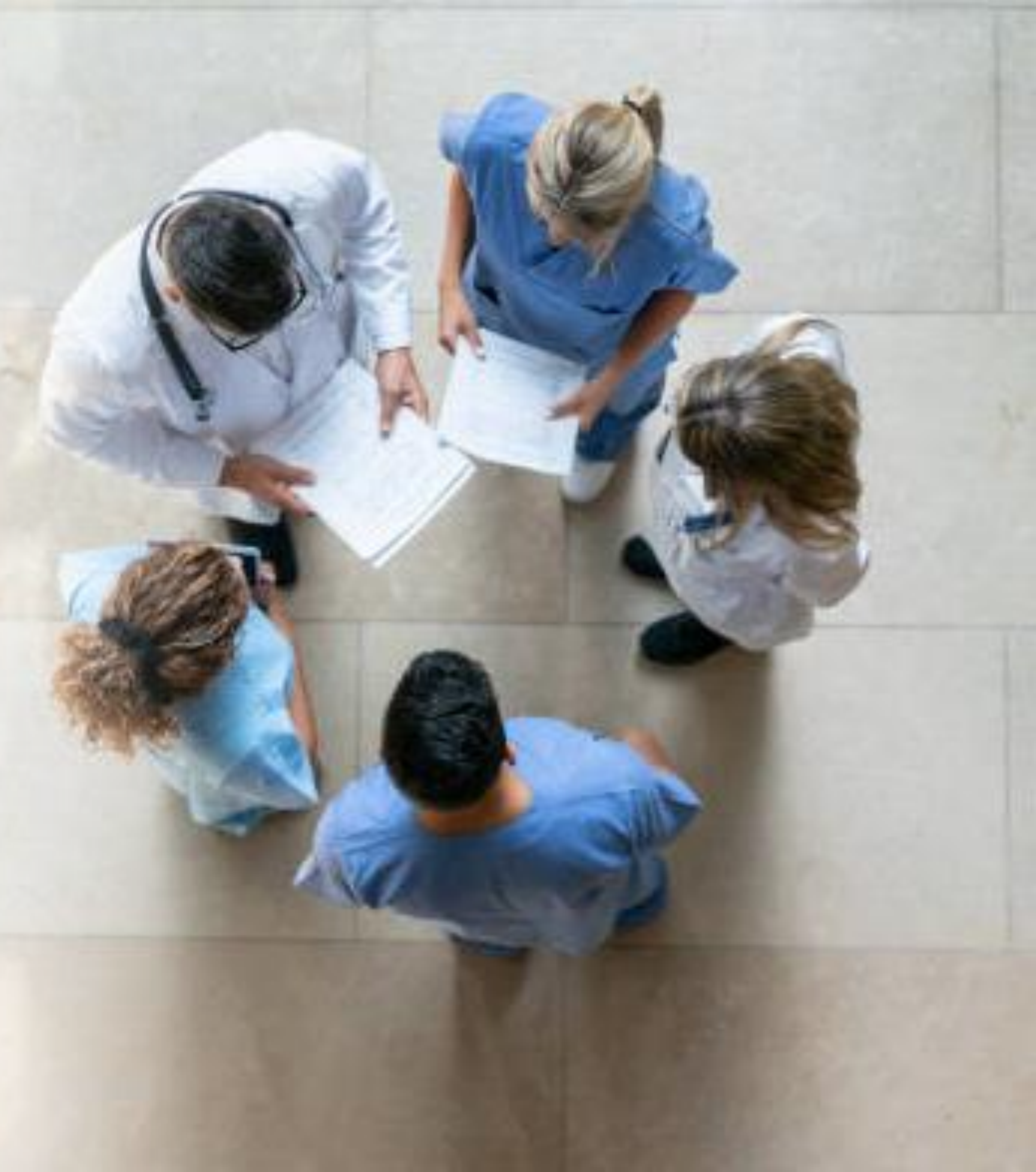

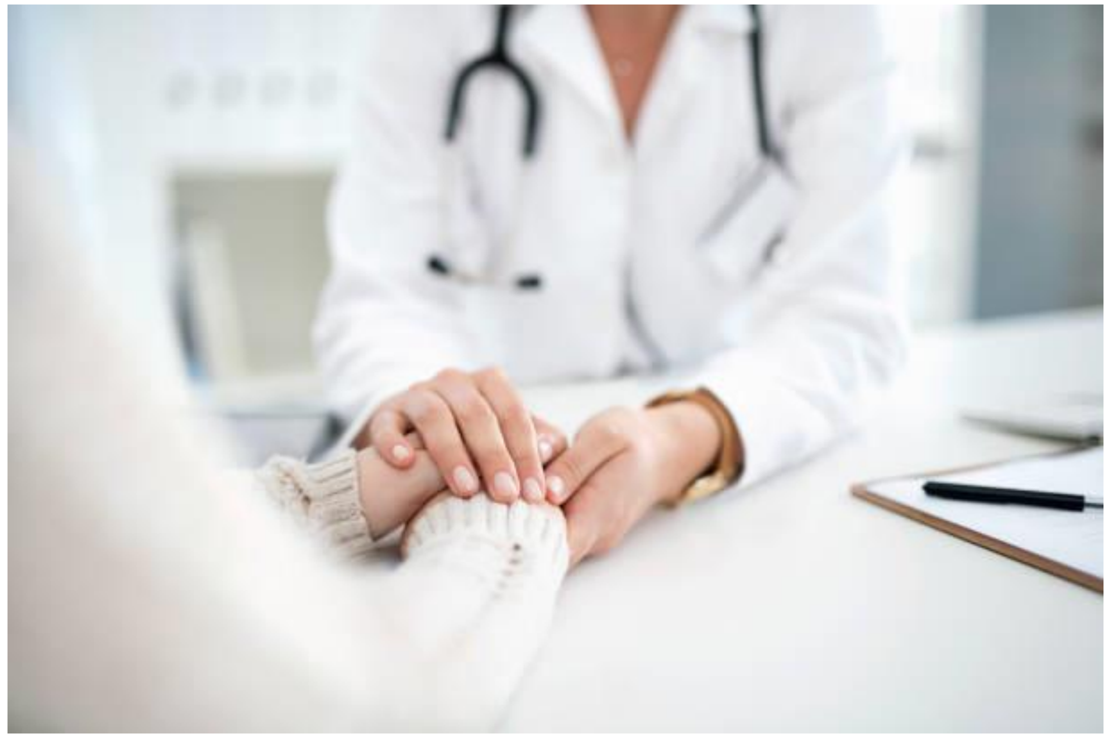

Providing Comfort

Providing Comfort & Strategies

- Reassure
  - Dealing with the situation will take time
- Legitimize & Normalize
  - They are not alone in their experience & feelings
- Healthy vs Unhealthy Behaviors
  - What has been successful for you in coping?
  - What tends to help you in stressful situations?

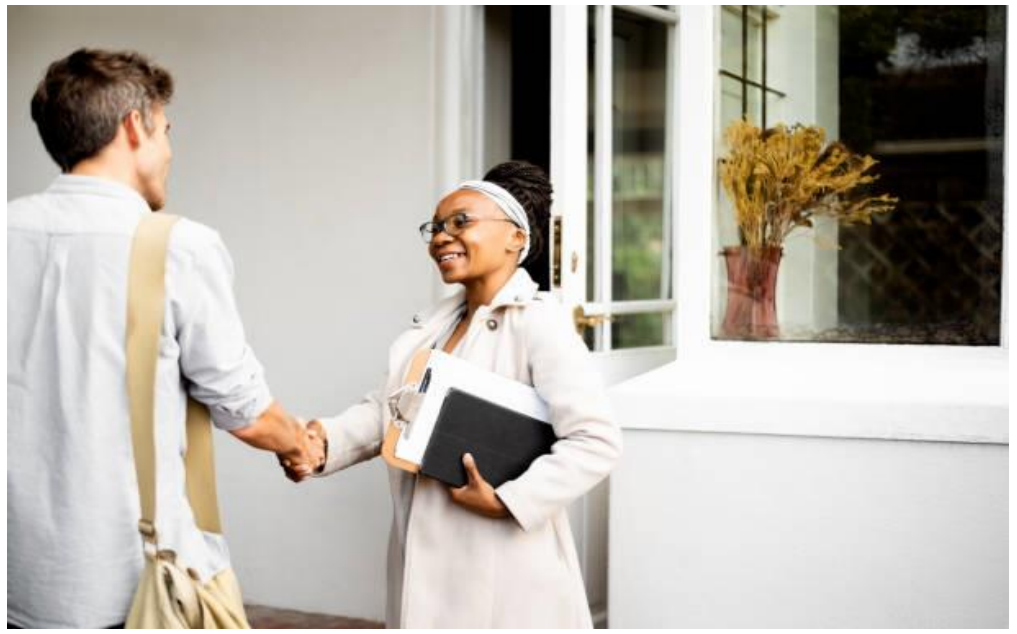

Follow-Up

Provide Information and Follow-up

- Let them know you are here for them
- Offer the opportunity to follow-up x1 or check in when it seems appropriate
- Share additional resources and offer to refer them to other therapeutic interventions if needed
  - Spiritual Care Services
  - UTSW Outpatient Psychiatry Clinic
  - Headspace
  - NOMAD (Navigating Our Multifaceted Acute Distress)
  - National Suicide Hotline: 9-8-8
  - Jaime Harry, LCSW Program Director. CALM Peer Support, Faculty Wellness Program

Demo & Skills Practice

Providing Comfort

- Reassure
- Explore Healthy Coping Behaviors

Follow up

- Offer resources as appropriate
- Next steps

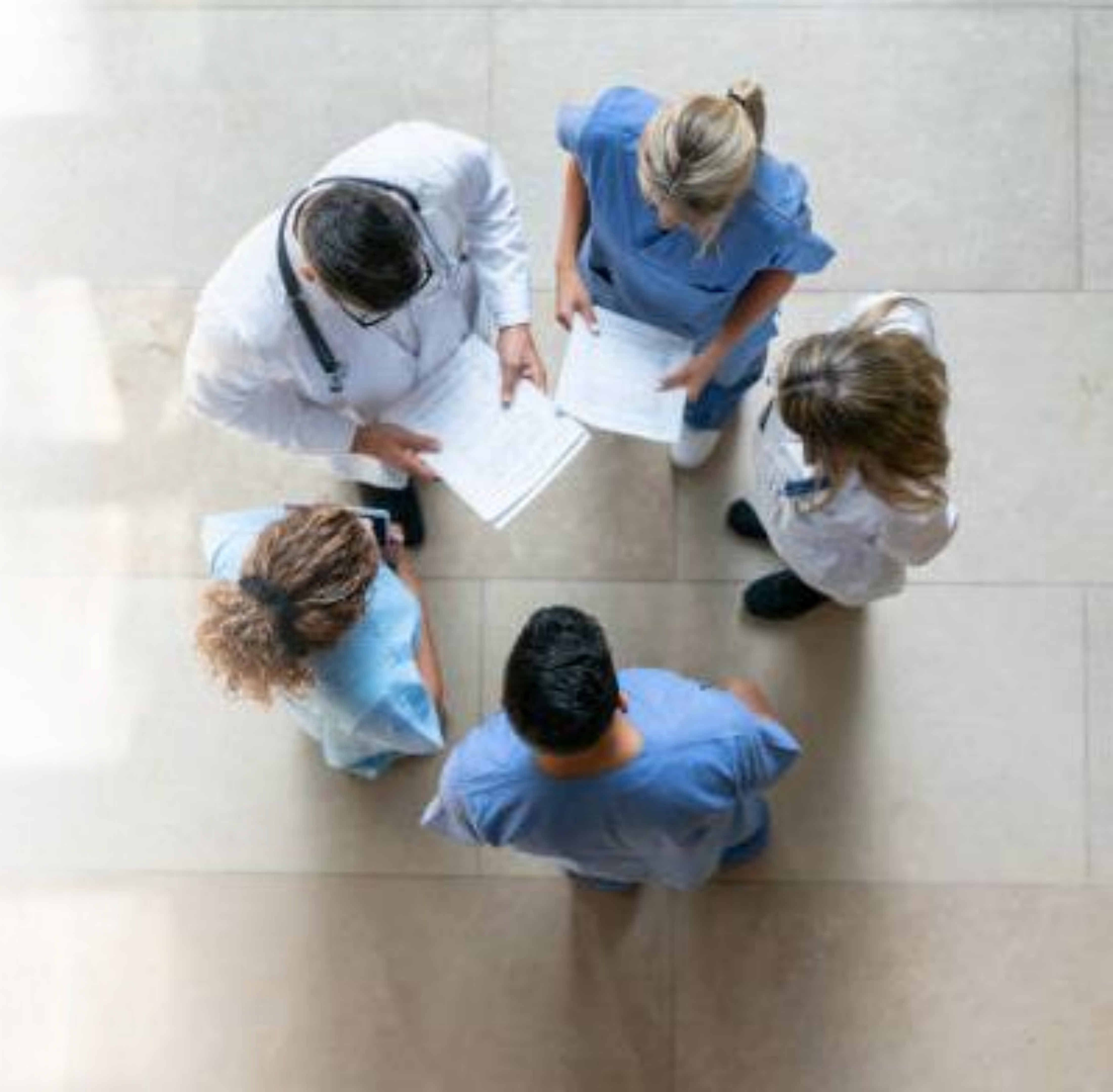

When to refer:

- Specialized psychotherapy: Concerns relating to having a significant personal crisis related to the event
- Patient Safety: Concerns regarding specific quality issues relating to the event
- Spiritual Care Services: Concerns regarding sense of spirituality and beliefs
- **Title IX Disclosures and reports regarding sexual harassment, sexual assault, dating violence, domestic violence, stalking, or other sexual misconduct committed by or against a student, resident, or employee**

  NOTE: Concerns about risk of self-harm, or suicidal ideation: remain with individual and follow organizational protocol

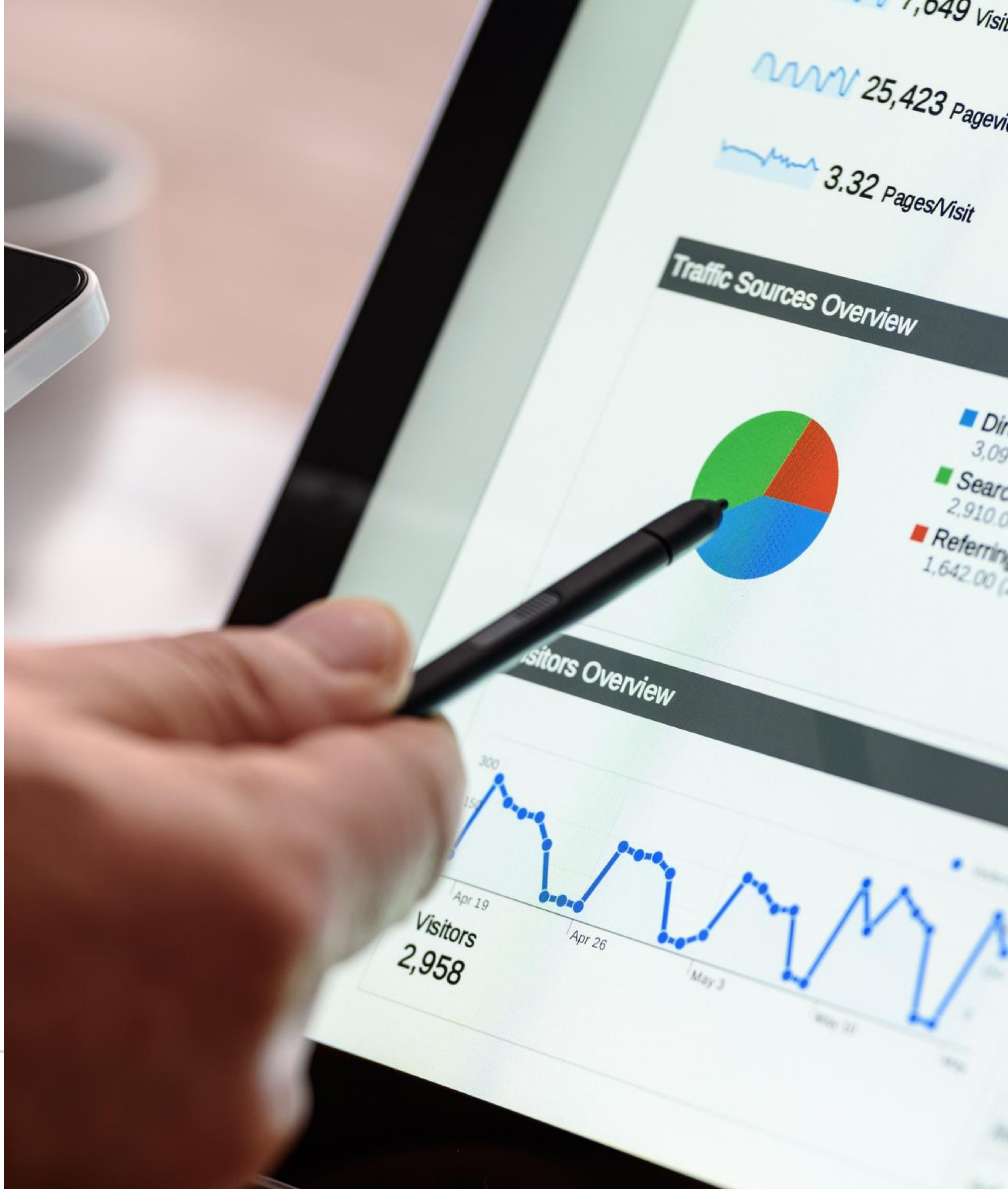

Bringing it All Together

The role of peer support in the Second Victim Trauma Trajectory

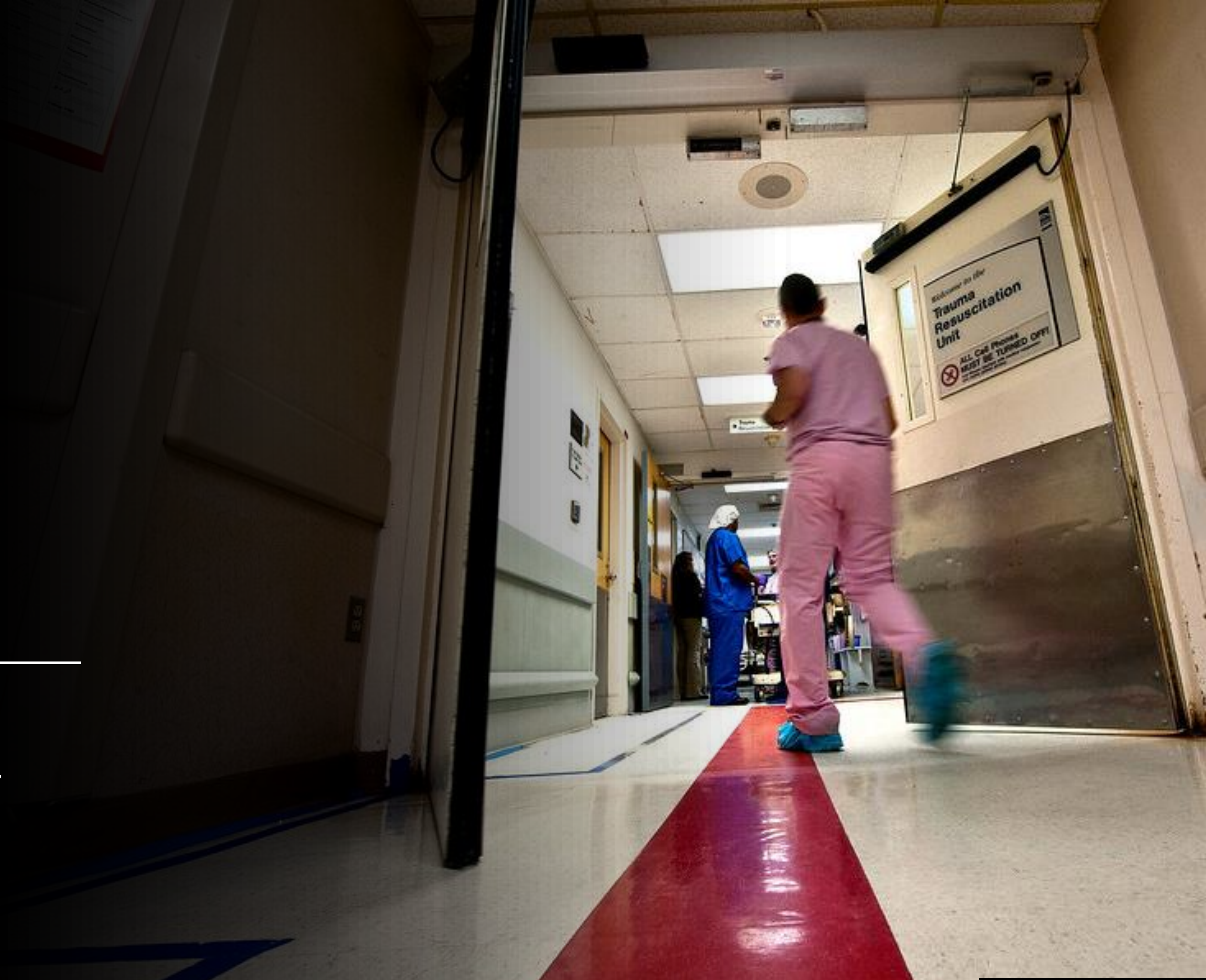

This Photo by Unknown author is licensed under CC BY-NC.

Second Victim / Trauma Recovery Trajectory

STAGE 1
Chaos & Accident Response

STAGE 2
Intrusive Reflections

STAGE 3
Restoring Personal Integrity

STAGE 4
Anticipating the Inquisition

STAGE 5
Obtaining Emotional First Aid

STAGE 6
Moving On

STAGE 1: CHAOS & ACCIDENT

Responses

- Event recognized
- Tell someone/get help!
- Stabilize/treat patient
- May not be able to continue care of patient
- Sometimes inability to process event due to next acute patient

"Right after the... code, I was having trouble concentrating. It was nice to have people take over... that I trusted. I was in so much shock I don't think I was useful."

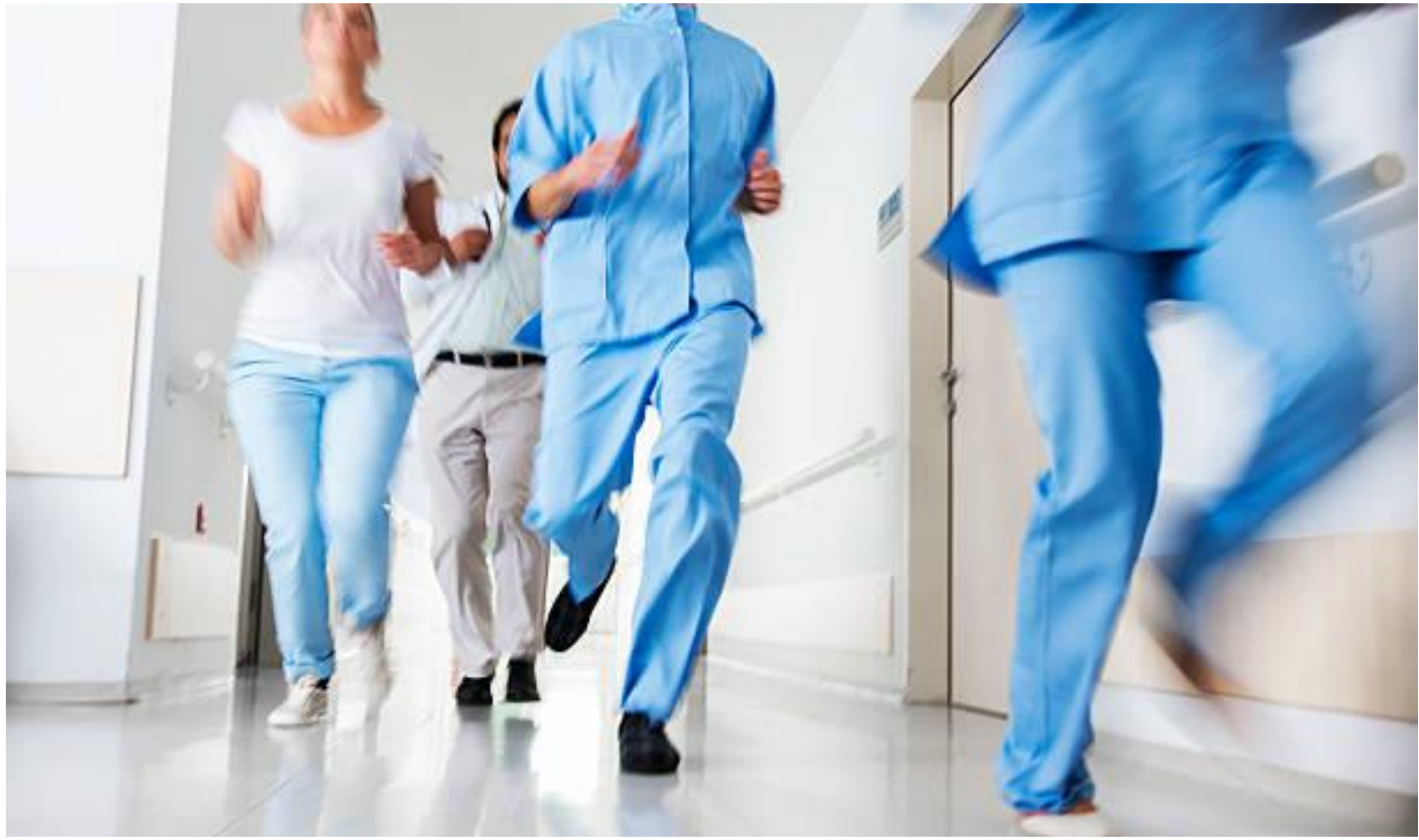

STAGE 2: INTRUSIVE REFLECTIONS

Responses:

- Re-evaluate scenario
- Self-isolate
- Poor sleeping habits and nightmares
- Feelings of internal inadequacy

"I started to doubt myself... There were some things that I thought maybe if I'd have done it this way it wouldn't have happened... but everything was clearer looking at things in retrospect. I lost my confidence for some time."

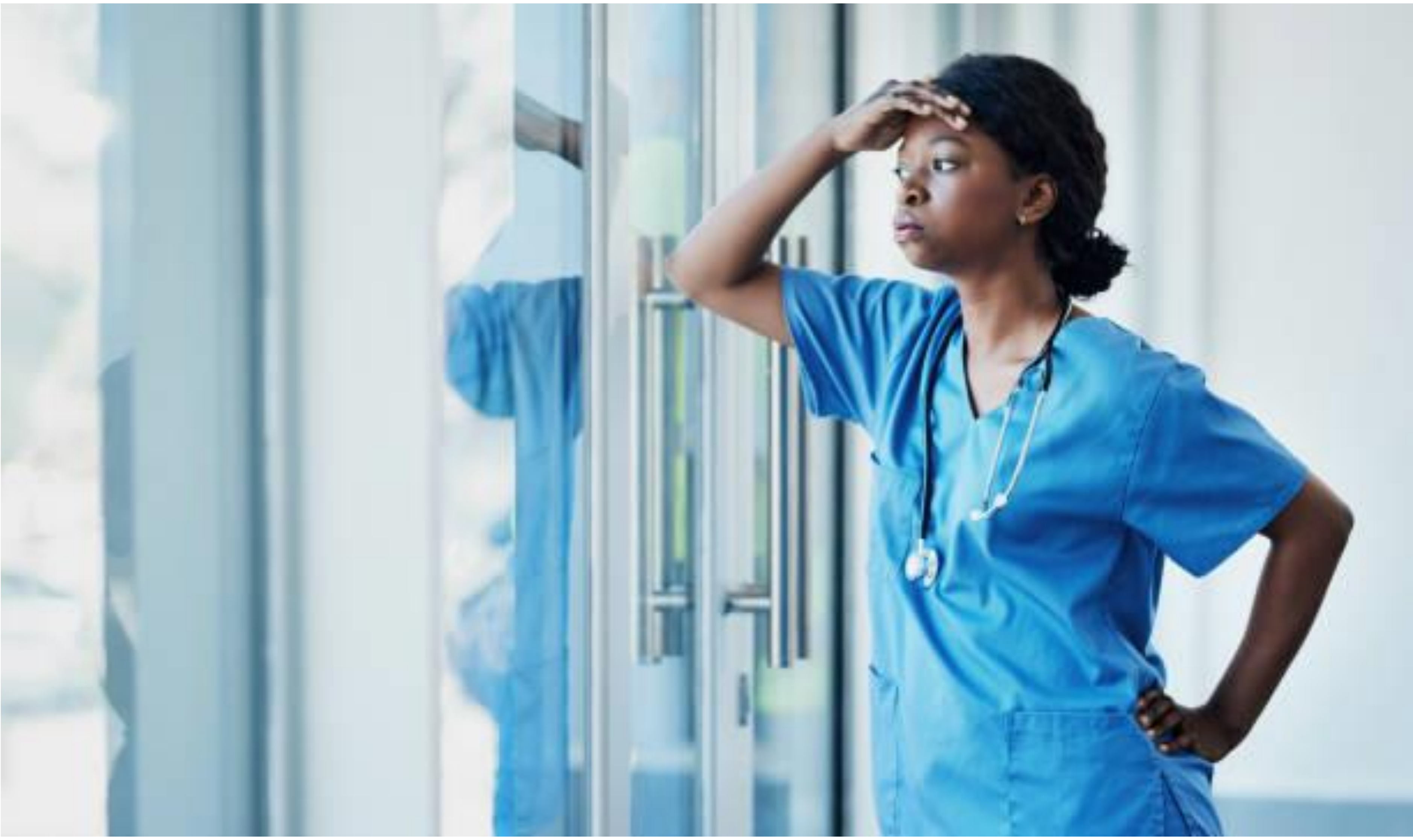

STAGE 3: RESTORING PERSONAL INTEGRITY

Responses:

- Desiring to be accepted back into the workplace
- Protecting of reputation
- Gossip mill
- Fear of judgement
- Fear of not being accepted

"I thought every single day for months I'd walk in and think everyone knows what happened… I thought these people are never going to trust me again."

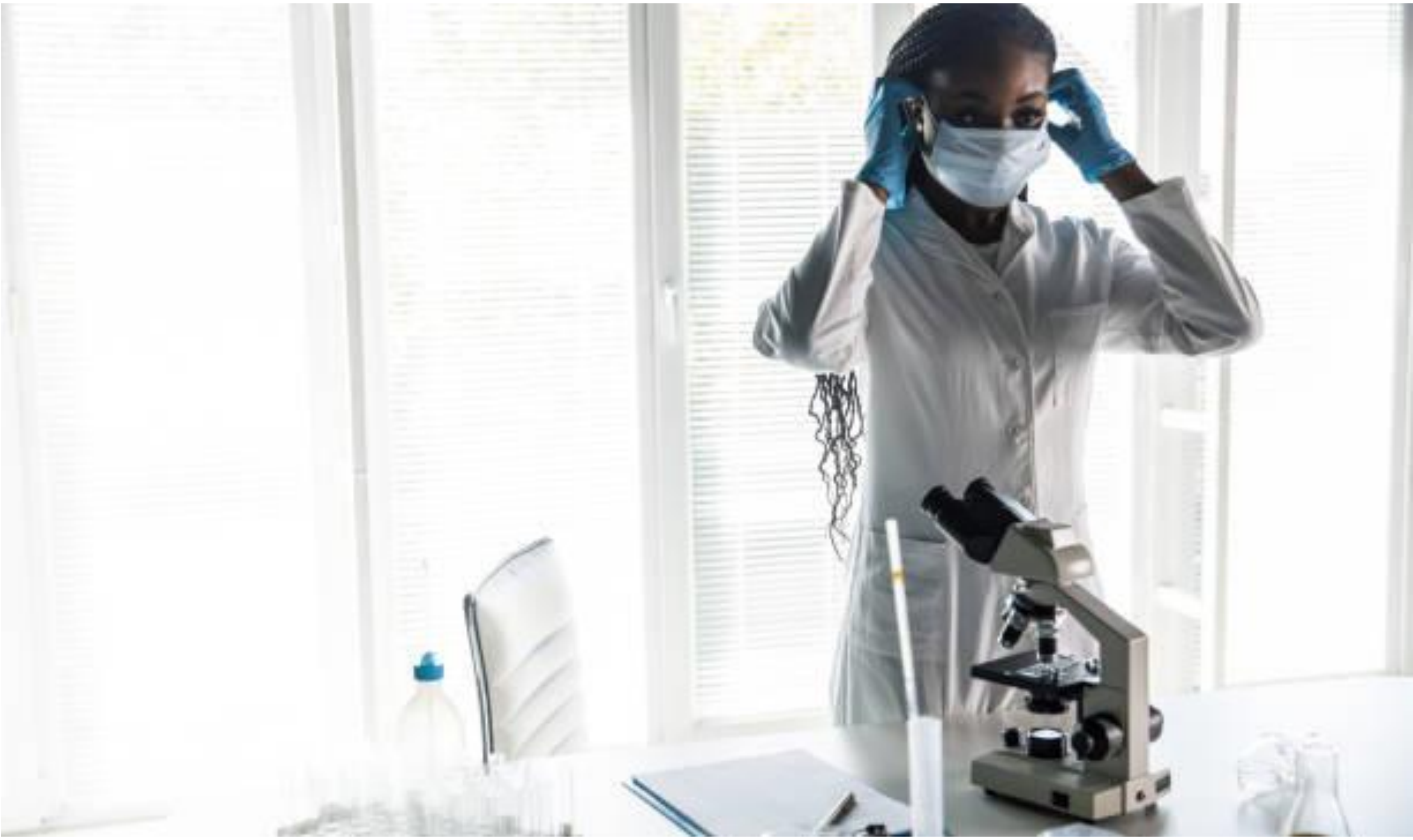

STAGE 4: ANTICIPATION OF THE INQUISITION

Responses:

- Realization of the level of seriousness
- Reiterate case scenario
- Responding to the "why's"
- Interacting with many different "event" responders
- Understanding event disclosure to patient/family
- Litigation concerns emerge

"I just wanted the questions to stop. It became overwhelming and I felt so alone"

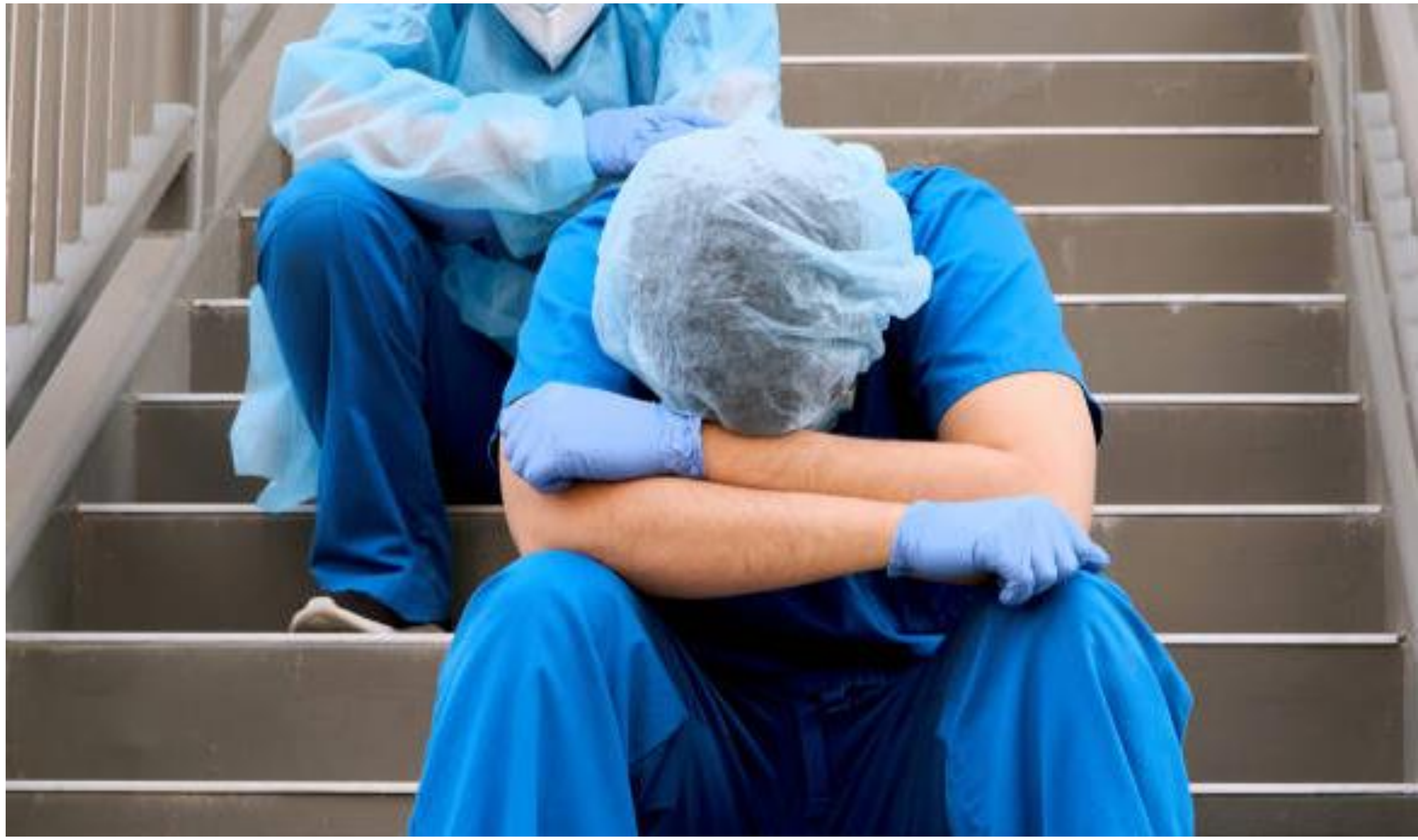

STAGE 5: OBTAINING EMOTIONAL FIRST AID

Responses:

- Identify who is safe to confide in
- Hoping someone will reach out
- Getting personal and/or professional support
- Litigation concerns emerge
- Confidentially

"There was nobody I could tell, not even my family. All I could say is I've had a really horrible day."

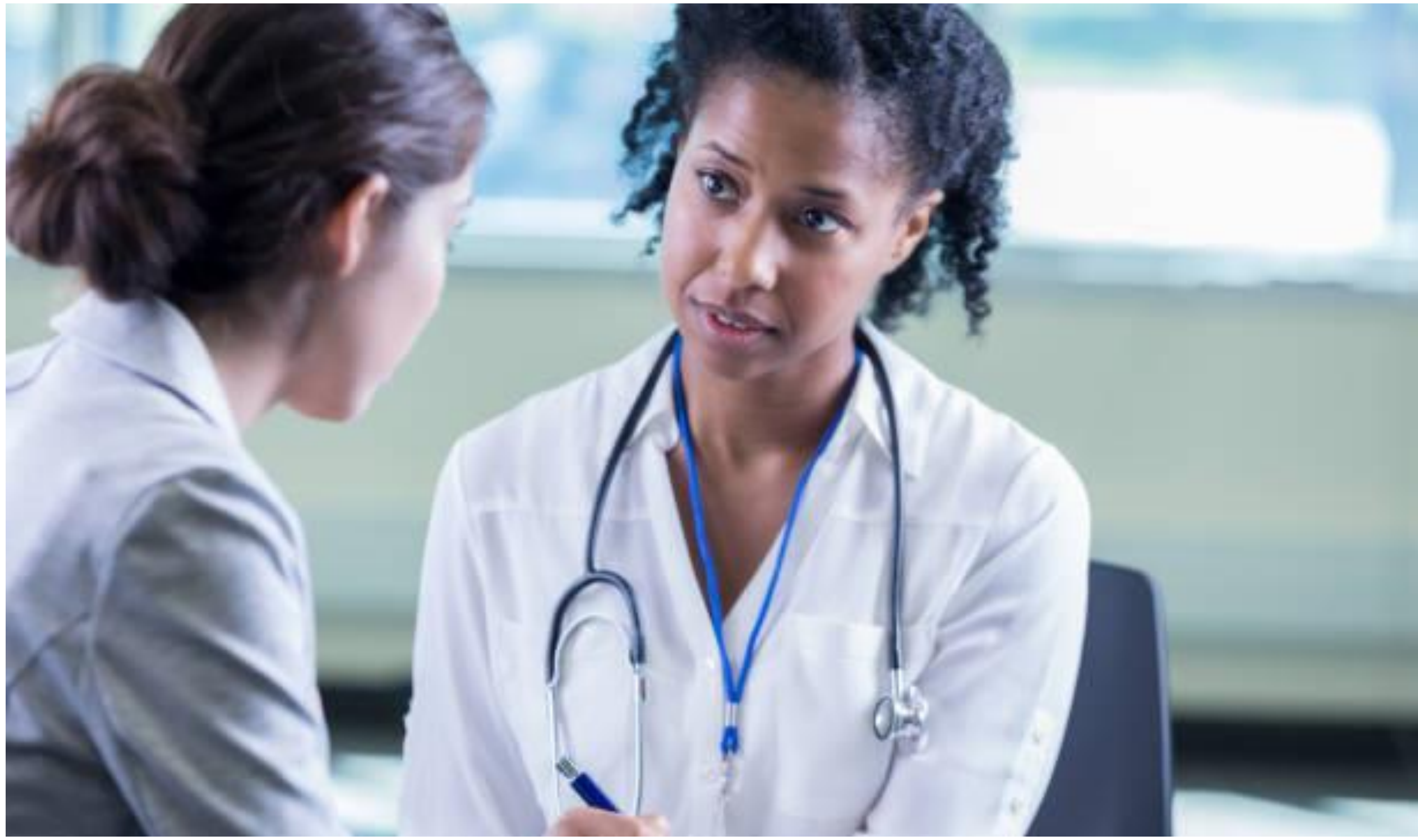

Stage 6: Moving on

STAGE 6: MOVING ON

DROPPING OUT

Response:

Feelings of inadequacy/failure
Leave current role by transferring to different department/role
Consider quitting profession altogether

A fresh start was good for me.

"I actually ended up moving to a different department. My new supervisor who oriented me expressed confidence and belief in me and helped me re-grow my own sense of confidence and self-belief."

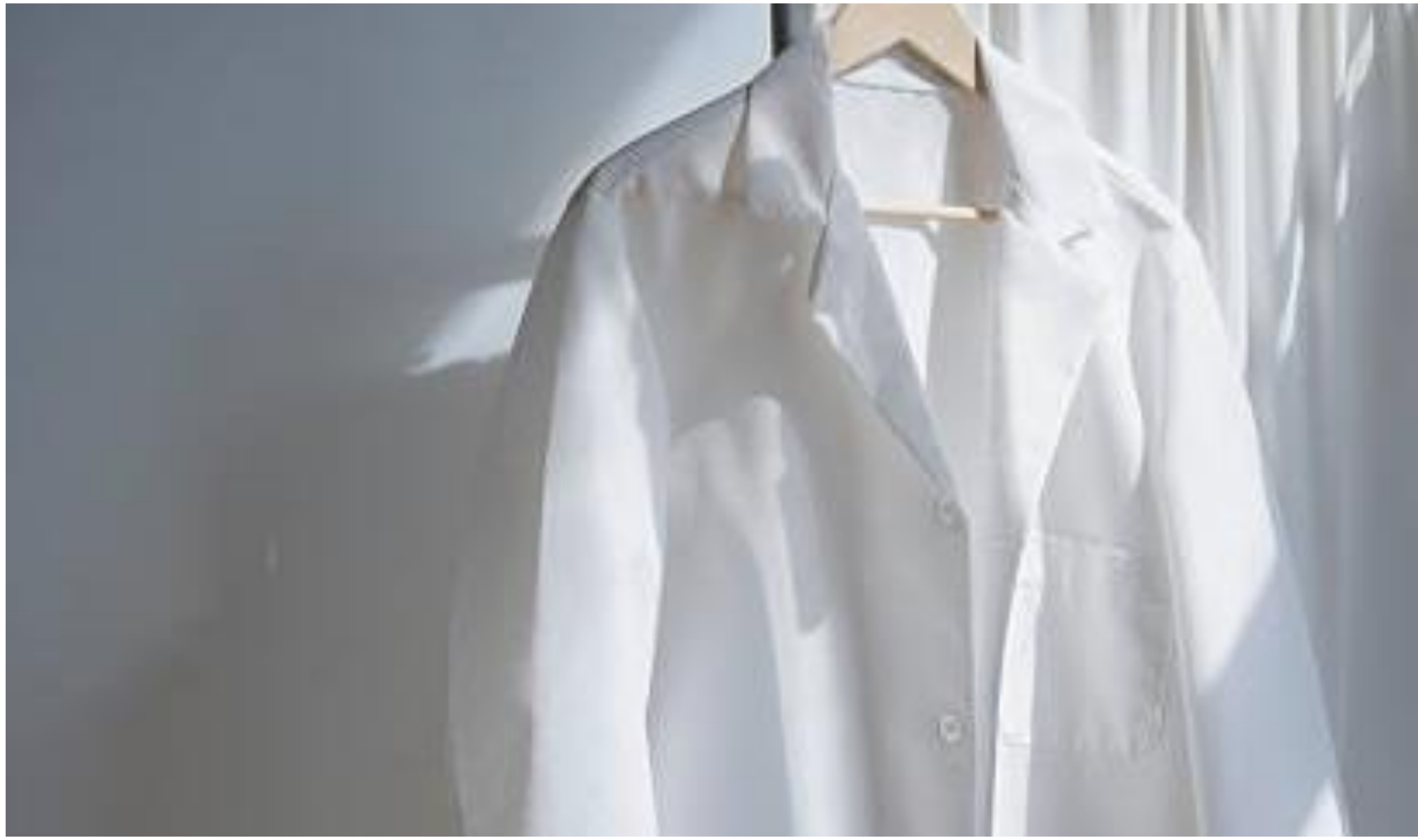

STAGE 6: MOVING ON

SURVIVING

Response:

Coping with what happened
Persistent sadness prevails
Trying to learn from the event
Never quite the same…

"I figured out how to cope and how to say yes, I made a mistake. And that mistake caused a bad patient outcome, but I haven't figured out how to forgive myself for that or forget it. It's impossible to let go."

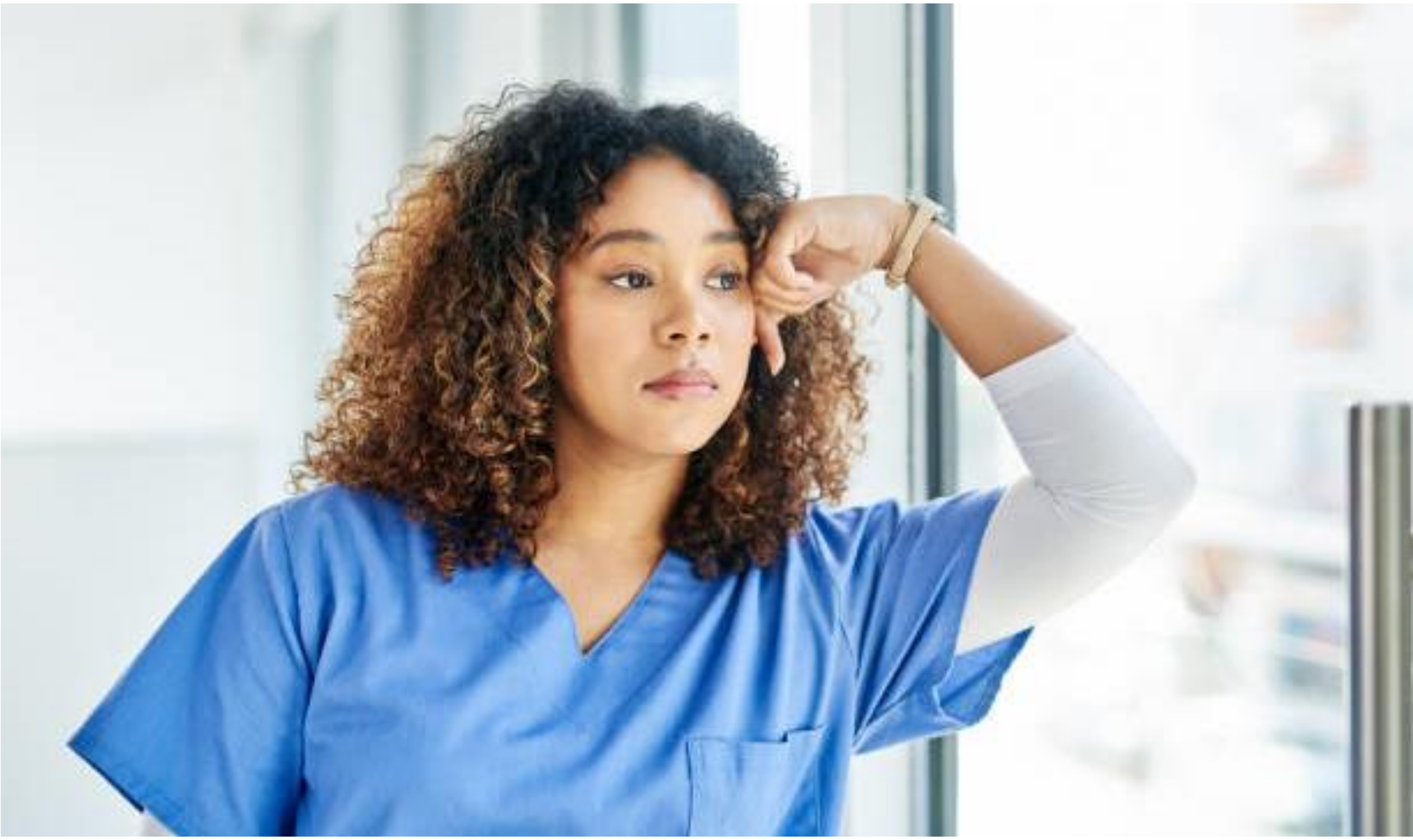

STAGE 6: MOVING ON

THRIVING

Response:

Does not base practice/work on one event
Minimal adverse effect from event
Advocates for patient safety initiatives
Tries to make a difference for the next patient/clinician

"I was questioning myself over and over again...but then I thought ... I've just had this experience in my life where I had to encounter this tragedy, but it made me a better person. It really did, and it gave me insight."

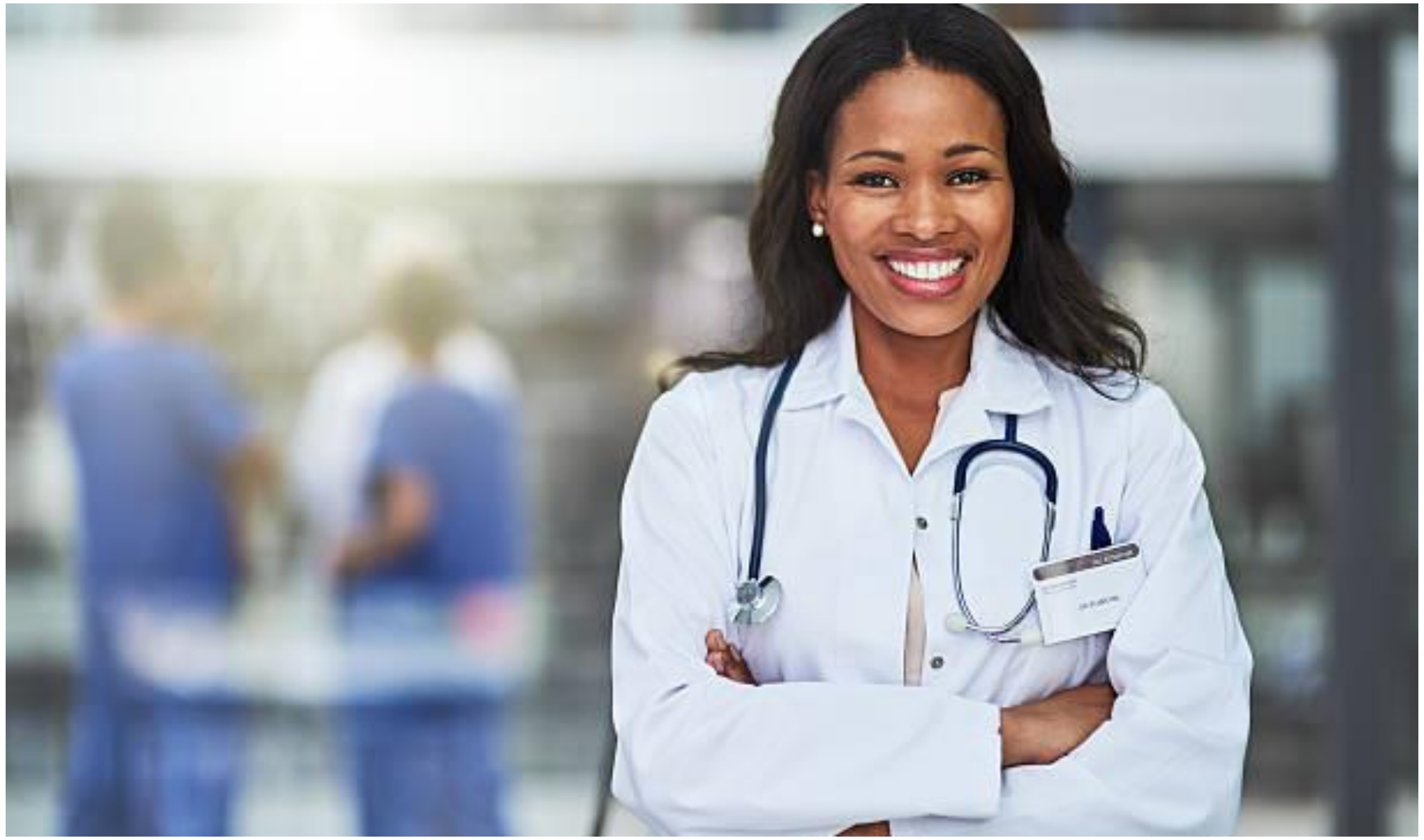

- Peer Practice (Triads)

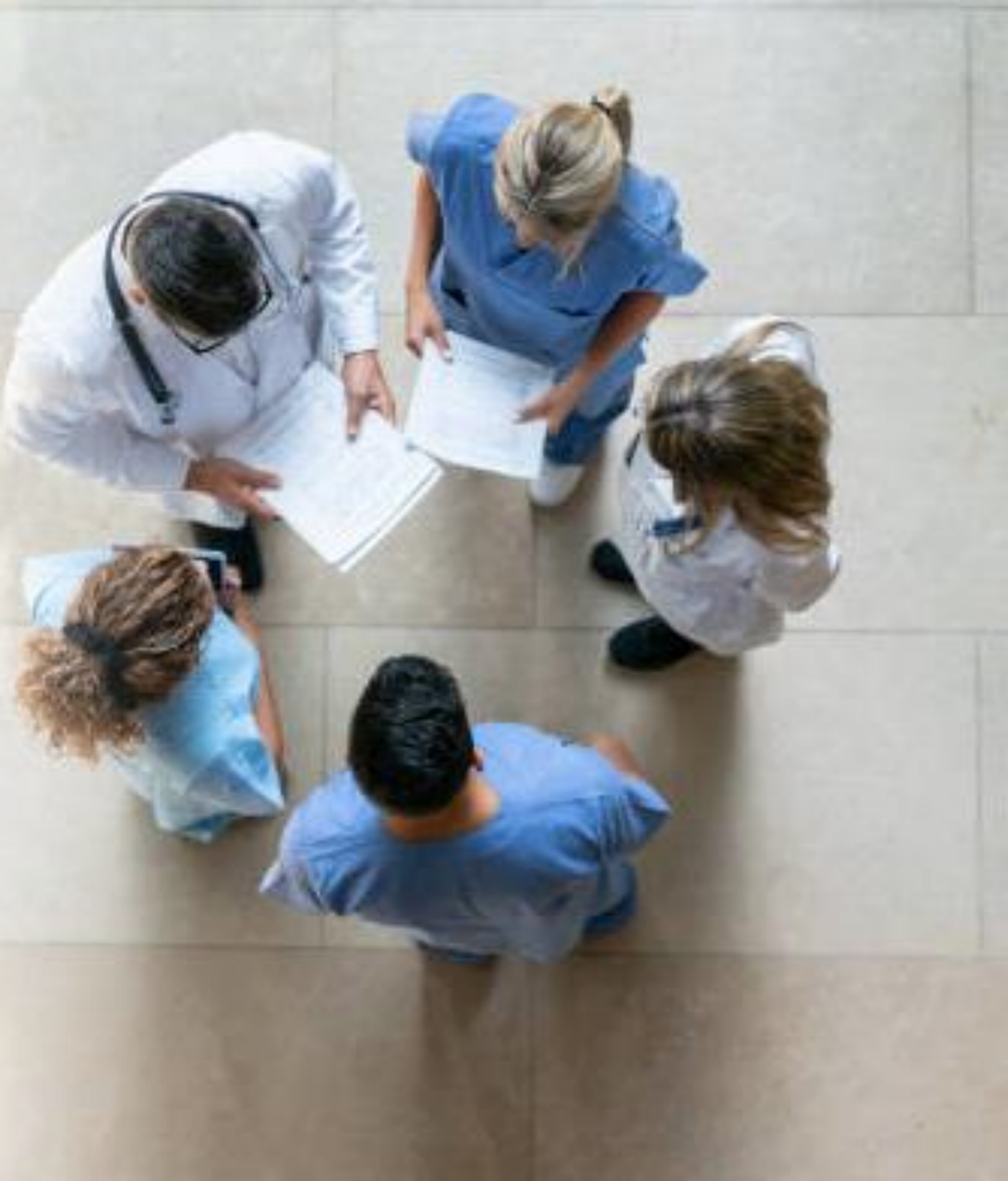

Scenario Reflections

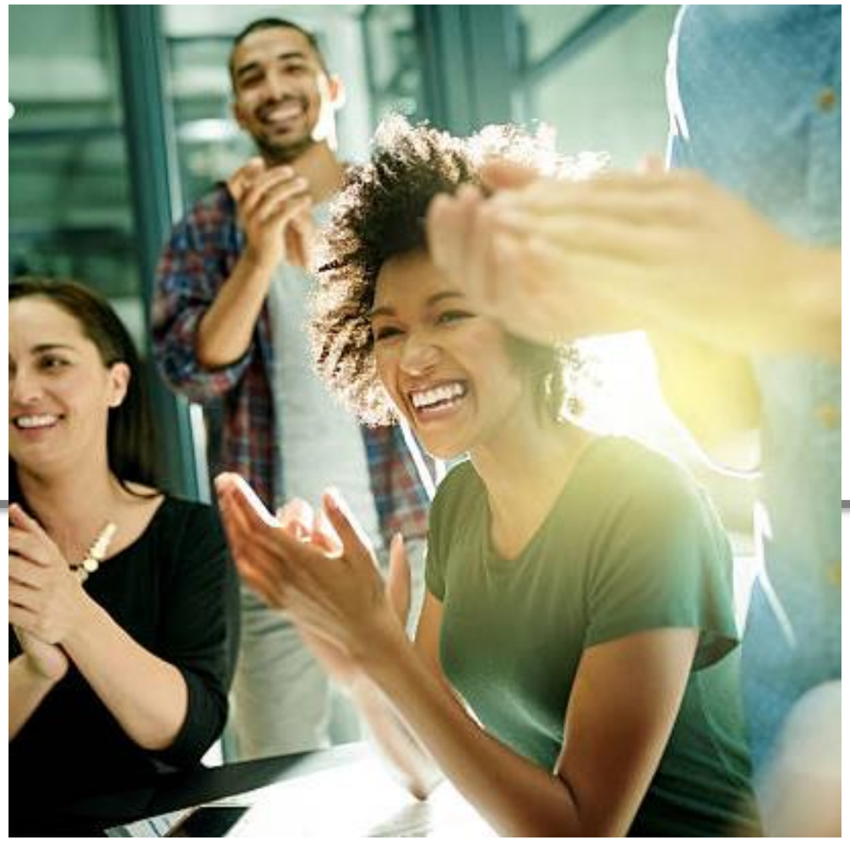

What went Well?

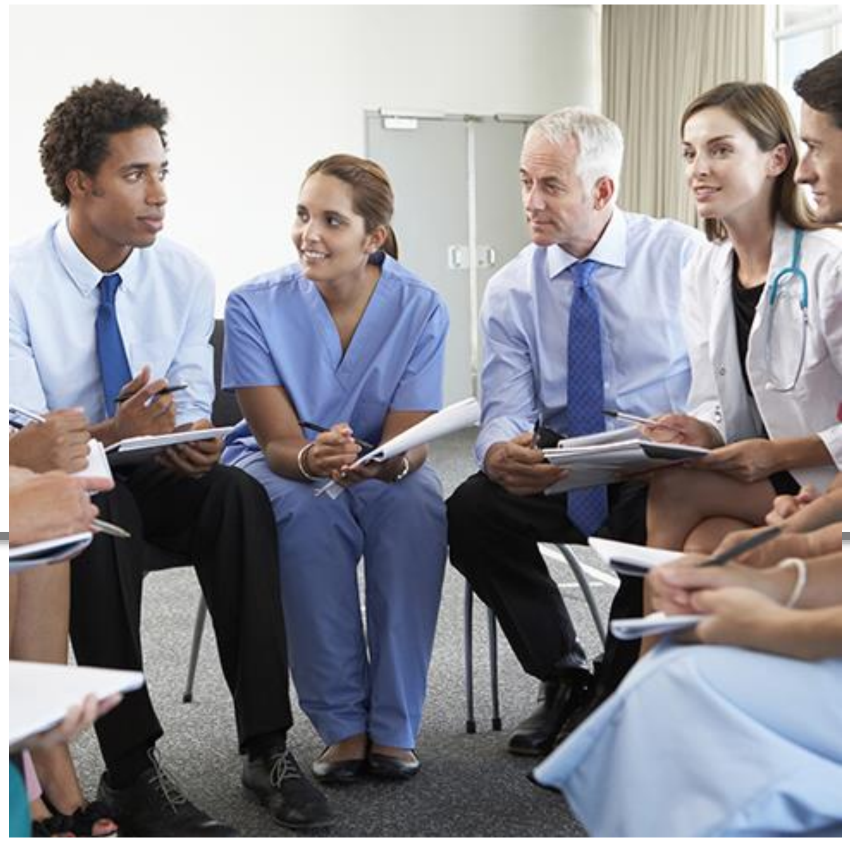

What would you have done differently?

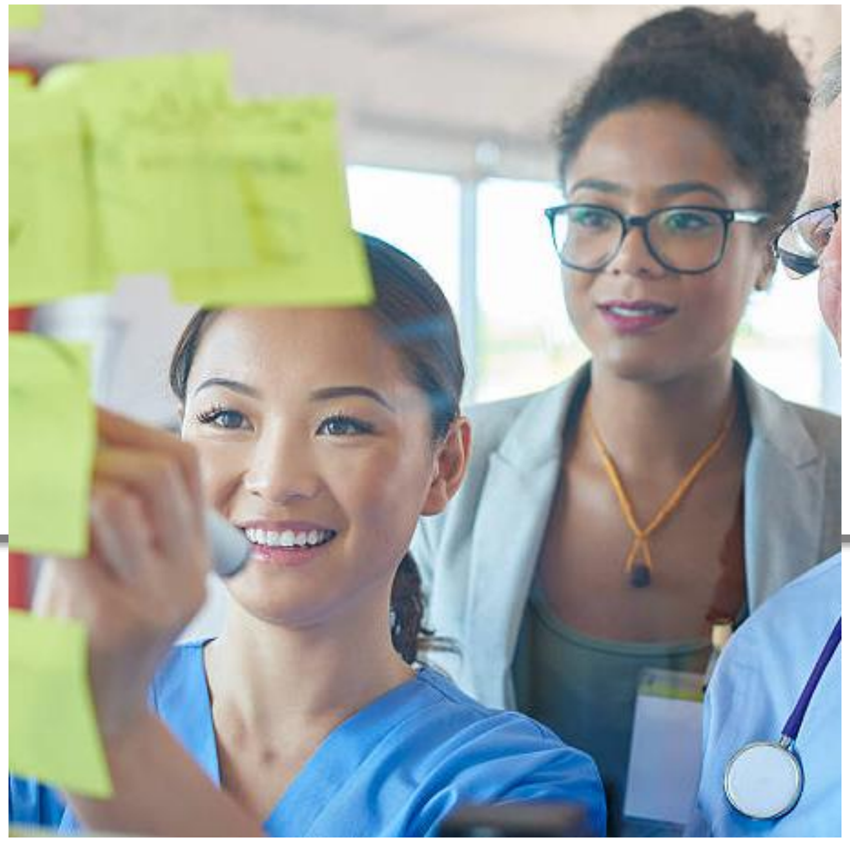

What one tip did you identify during your session?

Words of Caution

Take care of Yourself → Understand the limits in your role → Reach out to your peer support leader when things because overwhelming → Debriefing the debriefer is important too!

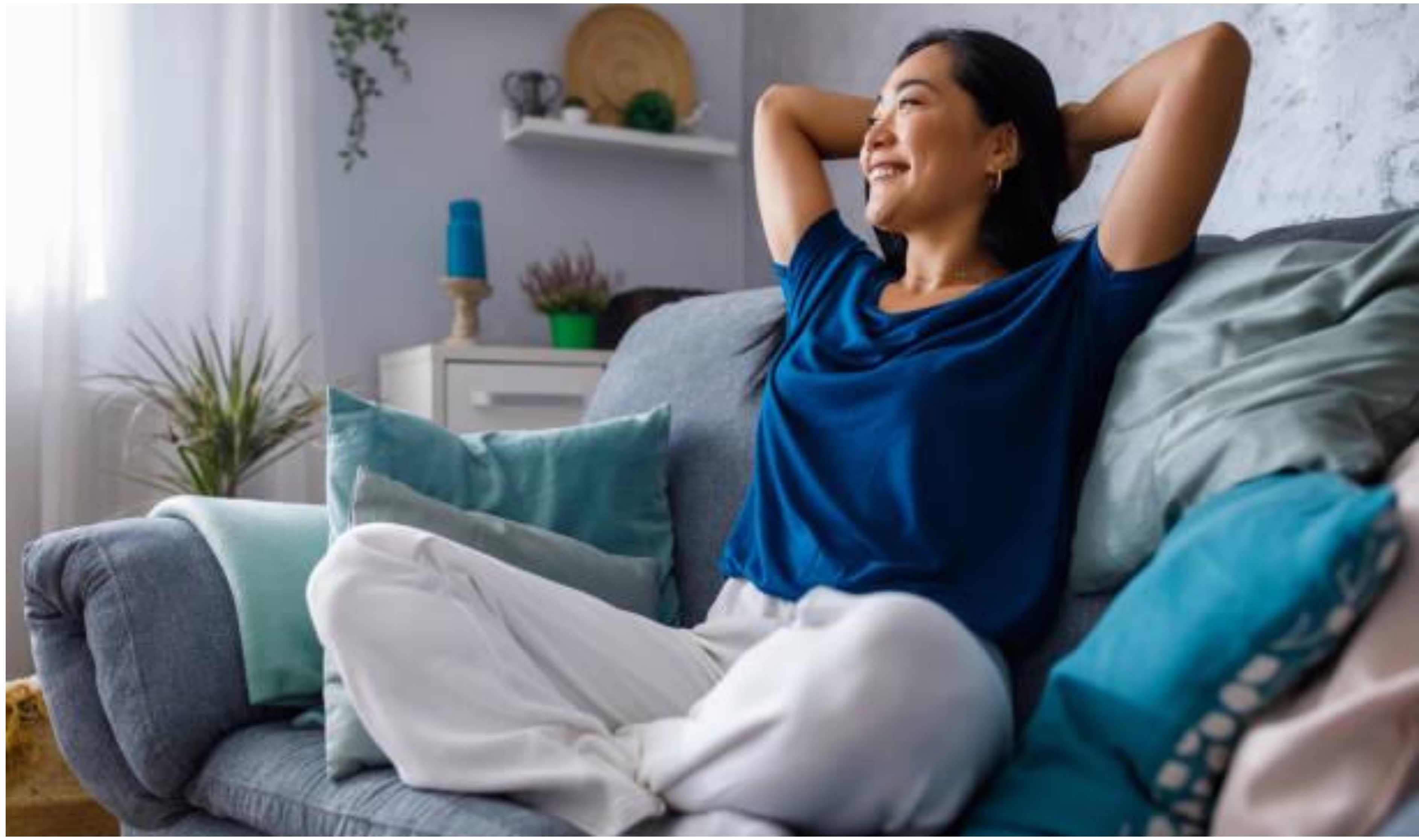

CALM Peer Supporters 2023

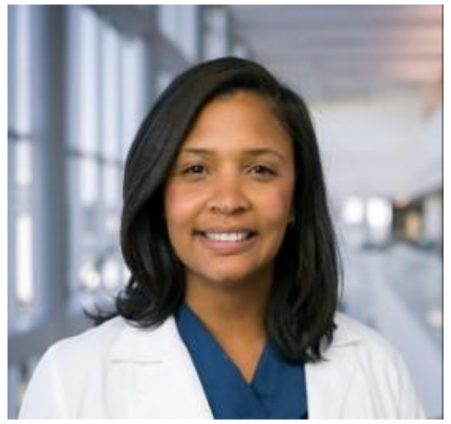

Andrea (Hill) Josey

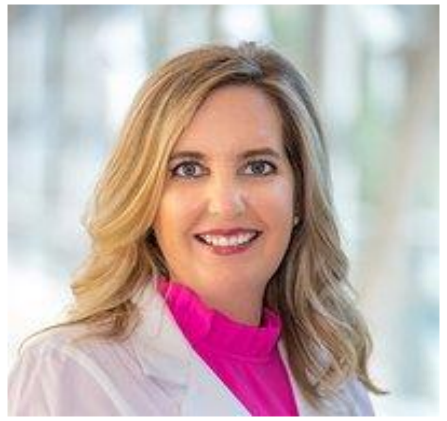

Angi Courtney

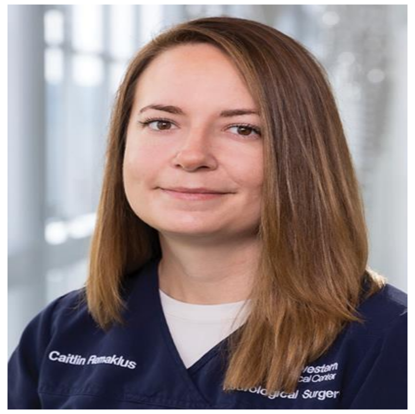

Caitlin Remaklus

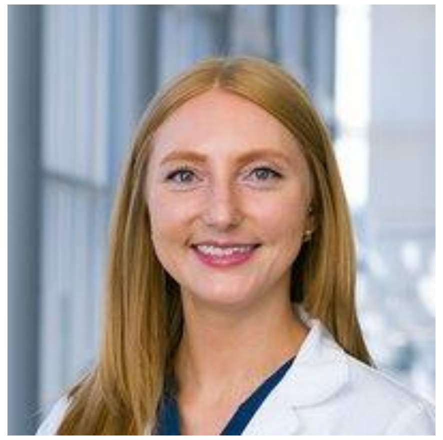

Grace Bouvier

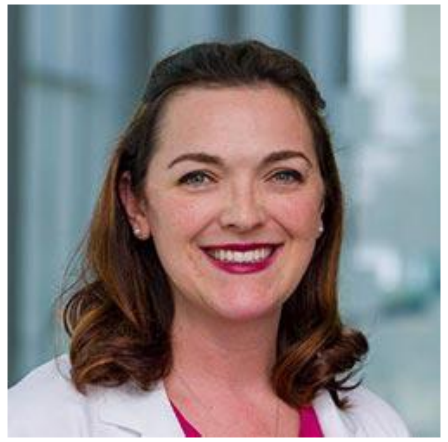

Jennifer Kargel

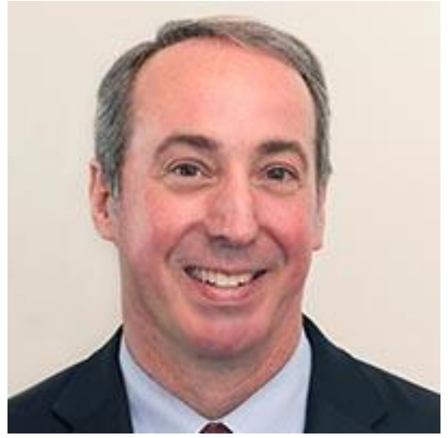

Jonathan White

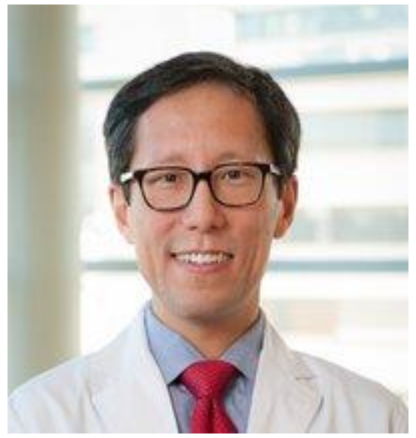

Jonathan Cheng

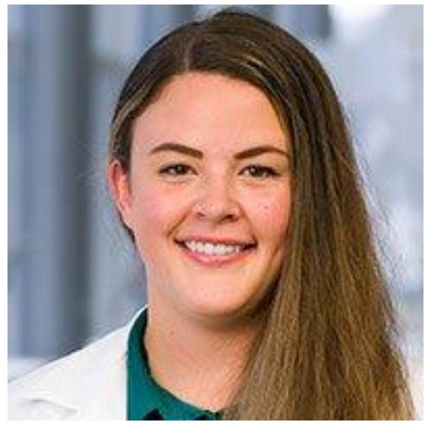

Laura Ervin

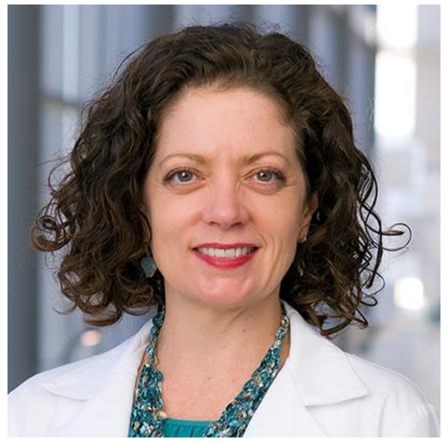

Laura Kirk

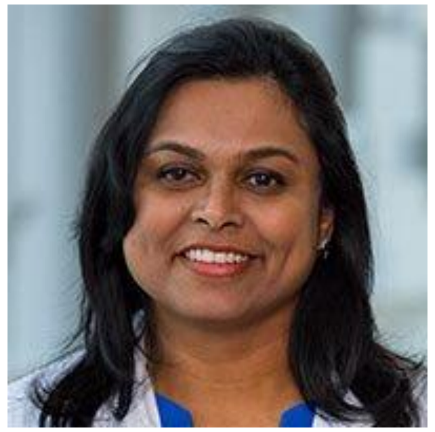

Nisha Thekkedam

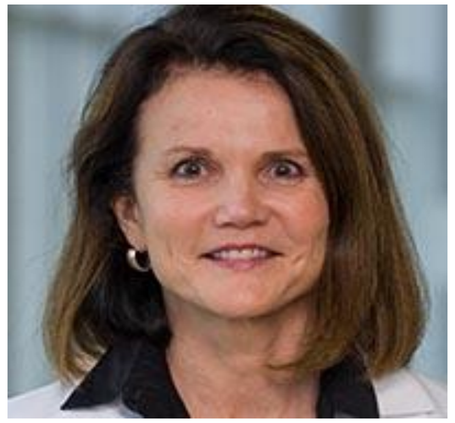

Scarlett Harden

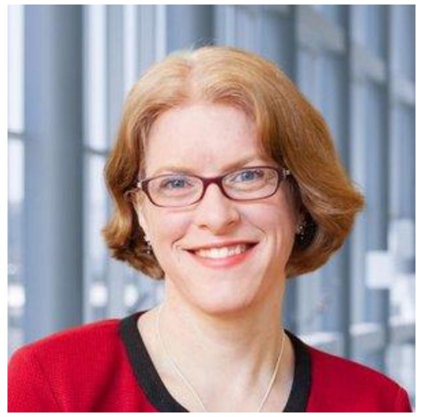

Susan Matulevicius

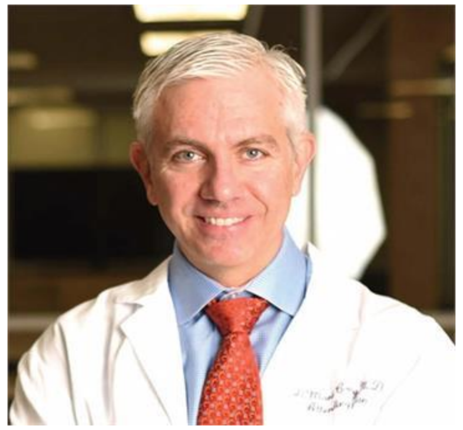

Mark Courtney

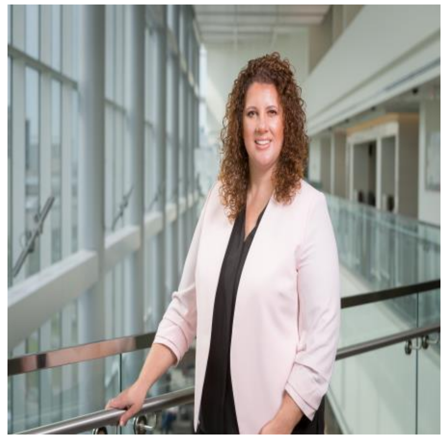

Rachel Kelley

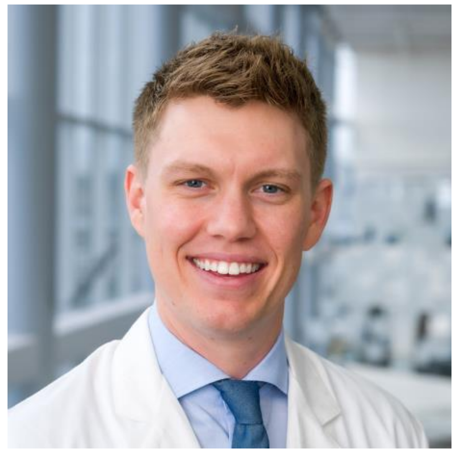

IV Mirus

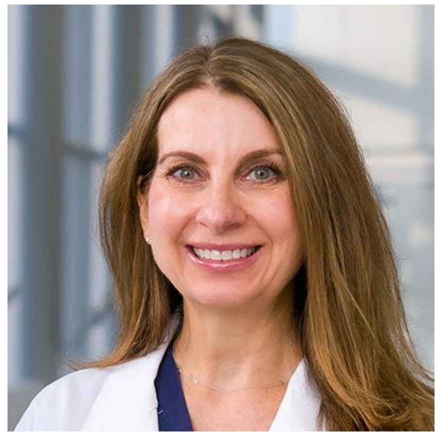

Jacqueline Presson

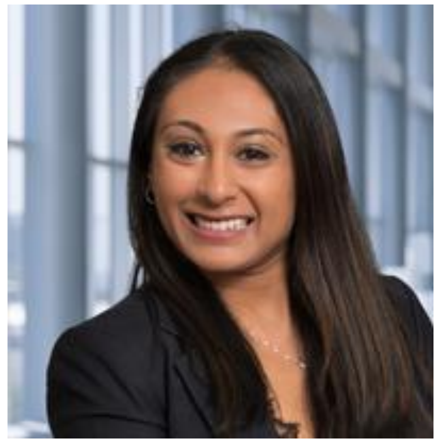

Roma Mehta

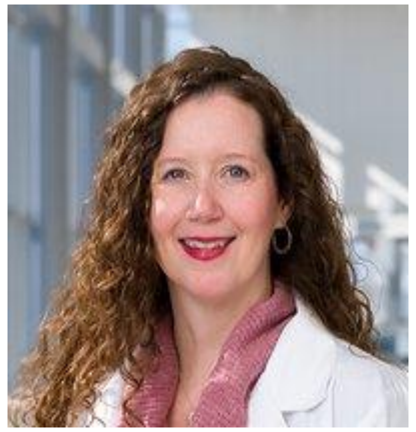

Pamela Garcia

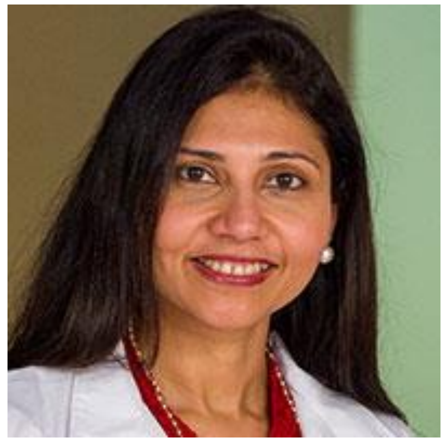

Rina Sanghavi

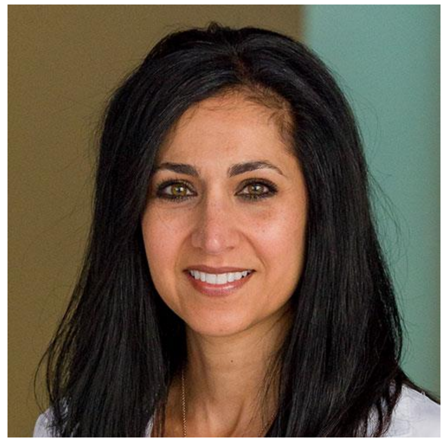

Rana Said

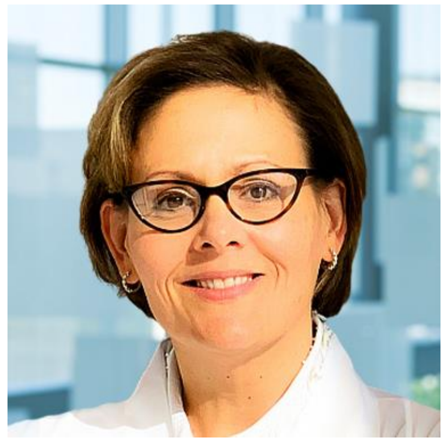

Vanessa Rogers

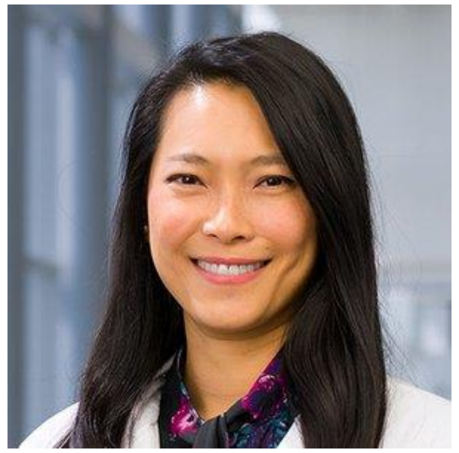

Joy Chen

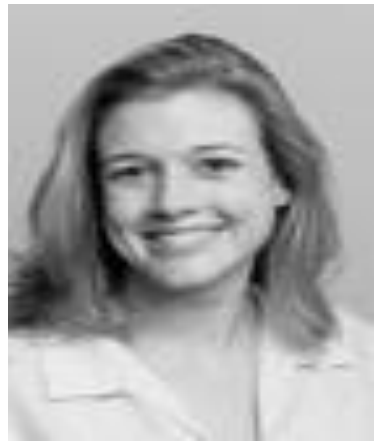

Ellen O' Connell

Keep in Touch

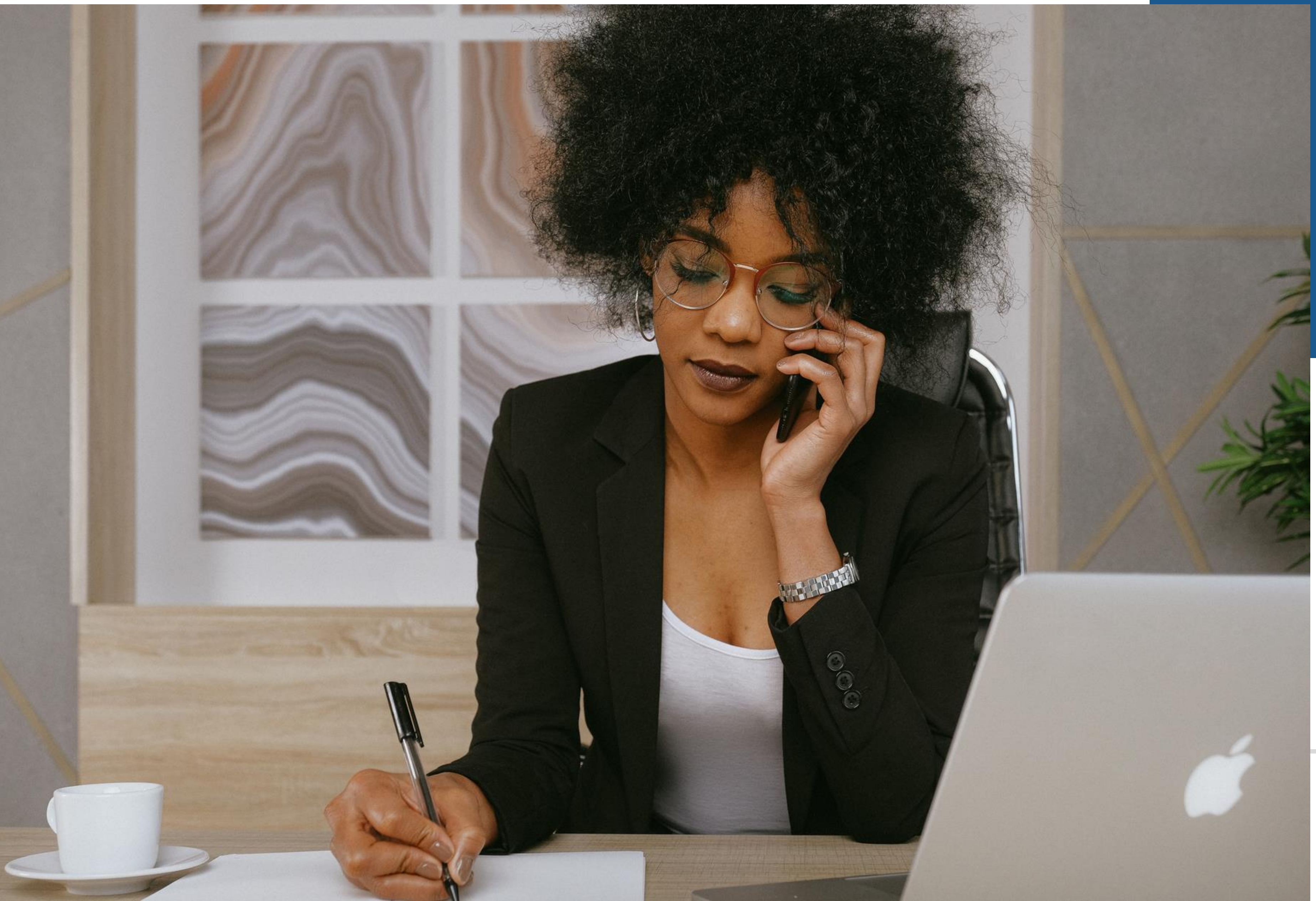

Contact us to get more info

CalmPeerSupport@UTSouthwestern.edu

5323 Harry Hines Blvd;
McDermott Bldg - Floor 4;
Dallas, TX 75390

[Faculty Wellness - UT Southwestern, Dallas, Texas]

Presented By : Jaime Harry, LCSW
