# Supplementary material for: Effect of psychological first aid training for fellows on resident burnout and distress in the intensive care unit
Source: PLoS One. 2026 Feb 9;21(2):e0340456. doi: 10.1371/journal.pone.0340456 (PMC12885303; doi:10.1371/journal.pone.0340456)
Supplement: S2 Appendix — (DOCX) [file pone.0340456.s002.docx]

Psychological First Aid Cases

Case 1

A 65 year old female with a history of small cell lung cancer is admitted to the MICU with acute on chronic respiratory failure. The patient undergoes a CTPA in the ED. The radiology report results after the patient is transferred to the MICU and the patient is diagnosed with an acute PE. The admitting resident orders therapeutic Lovenox which is given. Four hours later, the nurse alerts the resident that the patient has an acute mental status change and is now obtunded. The patient is intubated for airway protection and transported for a stat head CT, which demonstrates an acute intracerebral hemorrhage and new metastatic brain lesions.

Case 2

A 78 year old female with a history of widely metastatic breast cancer is admitted to the MICU with septic shock. On initial discussions regarding code status, the patient suggests that she would not like aggressive interventions but wants to talk to her daughter, who is her MPOA, before opting for DNAR. Her daughter arrives and urges that her mom is a fighter and she be kept full code, which her mother then agrees to. The patient’s condition deteriorates. Her vasopressor needs continue to escalate and she develops a worsening metabolic acidosis. She is intubated and a dialysis line and arterial line are placed. The intern and resident discuss the patient’s poor prognosis with her daughter and recommend her code status be changed to DNR, but her daughter maintains that her mom would want to be “brought back.” A couple of hours after this conversation, the patient suffers a PEA arrest. A code is run by the resident for 35 minutes before the attending declares the patient dead.

Case 3

A 65 year old male with a history of type 2 diabetes, hypertension, and COPD is admitted to the MICU for acute on chronic hypercarbic respiratory failure secondary to a COPD exacerbation. He is started on BiPAP but requires intubation for worsening respiratory acidosis. He remains intubated in the MICU for several days during a time when the MICU census and acuity is very high. Daily rounds were lasting into the afternoon and frequently being interrupted as the team needed to tend to crashing patients. Eight days into his hospital course, he develops tachycardia and the fellow observes that his minute ventilation is higher. CTPA confirms that he has an acute PE. The resident notices that he did not have DVT prophylaxis ordered despite the checklist in the intern’s daily progress notes indicating that he was on enoxaparin.
