# Supplementary material for: Effect of psychological first aid training for fellows on resident burnout and distress in the intensive care unit
Source: PLoS One. 2026 Feb 9;21(2):e0340456. doi: 10.1371/journal.pone.0340456 (PMC12885303; doi:10.1371/journal.pone.0340456)
Supplement: S3 Appendix — (PDF) [file pone.0340456.s003.pdf]

Human Research Protection Program

December 19, 2023

To: Traci Adams
Assistant Professor of Medicine

From: Stephanie Francisco, CIP
Sr. Regulatory Analyst
Human Research Protection Program Office

RE: Non-Regulated Research

Project Title: Implementation and evaluation of a quality improvement program to improve mental health of physicians in the medical intensive care unit

The UT Southwestern Human Research Protection Program (HRPP) has reviewed the above referenced project and determined that it does not meet the definition of research under 45 CFR 46.102 and therefore does not require IRB approval or oversight.

If you have any questions related to this communication or the UT Southwestern HRPP, please call 214-648-3060.

DocuSigned by:
Stephanie Francisco CIP
0AC9472F165C44D...
