# Supplementary material for: Effect of psychological first aid training for fellows on resident burnout and distress in the intensive care unit
Source: PLoS One. 2026 Feb 9;21(2):e0340456. doi: 10.1371/journal.pone.0340456 (PMC12885303; doi:10.1371/journal.pone.0340456)
Supplement: S5 Appendix — (DOCX) [file pone.0340456.s005.docx]

Supplementary Appendix B. Survey given to fellows immediately after PFA training

| My overall impression of the training was: | 1 – Extremely negative  2 – Negative  3 – Neutral  4 – Positive  5 – Extremely positive |
| --- | --- |
| My impression of the presenter was: | 1 – Extremely negative  2 – Negative  3 – Neutral  4 – Positive  5 – Extremely positive |
| Please rate your level of agreement with the following statement: The course achieved the learning objectives. | 1 – Strongly disagree  2 – Disagree  3 – Neutral  4 – Agree  5 – Strongly agree |
| Please rate your level of agreement with the following statement: Psychological first aid is a valuable tool for ICU clinicians. | 1 – Strongly disagree  2 – Disagree  3 – Neutral  4 – Agree  5 – Strongly agree |
| Please rate your level of agreement with the following statement: The course will lead to a change in my practice. | 1 – Strongly disagree  2 – Disagree  3 – Neutral  4 – Agree  5 – Strongly agree |
| How confident do you feel about performing psychological first aid with residents in the intensive care unit? | 1 – Not at all confident  2 – Slightly confident  3 – Confident  4 – Very confident  5 – Extremely confident |

What were the most helpful aspects of this training?

What was the least helpful aspect of this training? What can or should we change for next time?
