# Supplementary material for: Effect of psychological first aid training for fellows on resident burnout and distress in the intensive care unit
Source: PLoS One. 2026 Feb 9;21(2):e0340456. doi: 10.1371/journal.pone.0340456 (PMC12885303; doi:10.1371/journal.pone.0340456)
Supplement: S6 Appendix — (DOCX) [file pone.0340456.s006.docx]

Supplementary Appendix C. Survey given to fellows 3 months after PFA training.

Please circle an answer to the following questions.

1. Prior to completing the psychological first aid training, had you ever provided psychological first aid in the intensive care unit? If so, how often did you provide PFA during your ICU rotations?

Yes No

Frequency: Once Once a month Once a week Daily

1. Have you provided psychological first aid with anyone in the ICU since your training? If so, how often did you provide PFA during your ICU rotations?

Yes No

Frequency: Once Once a month Once a week Daily

1. Who have you used psychological first aid with? Circle all that apply.

Patients Family members of patients Residents Fellows Faculty N/A

1. Did the psychological first aid training change your practice?

Yes No

1. How effective are you in performing psychological first aid?

Extremely ineffective

Ineffective

Neutral

Effective

Extremely effective

1. If you provided psychological first aid since your training, did you feel that it helped your colleague, patient, or patient’s family member?

Yes No
