# Supplementary material for: Effect of psychological first aid training for fellows on resident burnout and distress in the intensive care unit
Source: PLoS One. 2026 Feb 9;21(2):e0340456. doi: 10.1371/journal.pone.0340456 (PMC12885303; doi:10.1371/journal.pone.0340456)

Supplementary Appendix G. Resident Survey.

Please circle one answer for each of the following questions Date ____________

Gender: Male Female

Age: ________

Race: White Black Other (fill in) _______________________

Hispanic Ethnicity: Yes No

Marital status: Married Not married

(If not married): Cohabitating with significant other: Yes No

Number of children living with you: _____________

Position: Attending Fellow Resident Intern

(if Attending) Faculty rank: Assistant professor Associate professor Full professor

(If resident): Which residency program are you in? UTSW IM residency Other(fill in) ________

(If IM resident): Do you plan to pursue a fellowship in pulmonary/critical care? Yes No

(If IM resident): Do you plan to pursue a fellowship in cardiology? Yes No

(If faculty) Primary practice site: CUH Parkland

Year started residency: ____________

Year started pulmonary and critical care fellowship: __________

Do you know how to access psychiatric assistance at UTSW? Yes No

Do you know any healthcare workers at UTSW who could not access psychiatry care in a timely manner?

Yes No

Time spent in MICU this academic year: < 1 month 1 month – 3 months > 3 months

Is this your first ever MICU rotation? Yes No


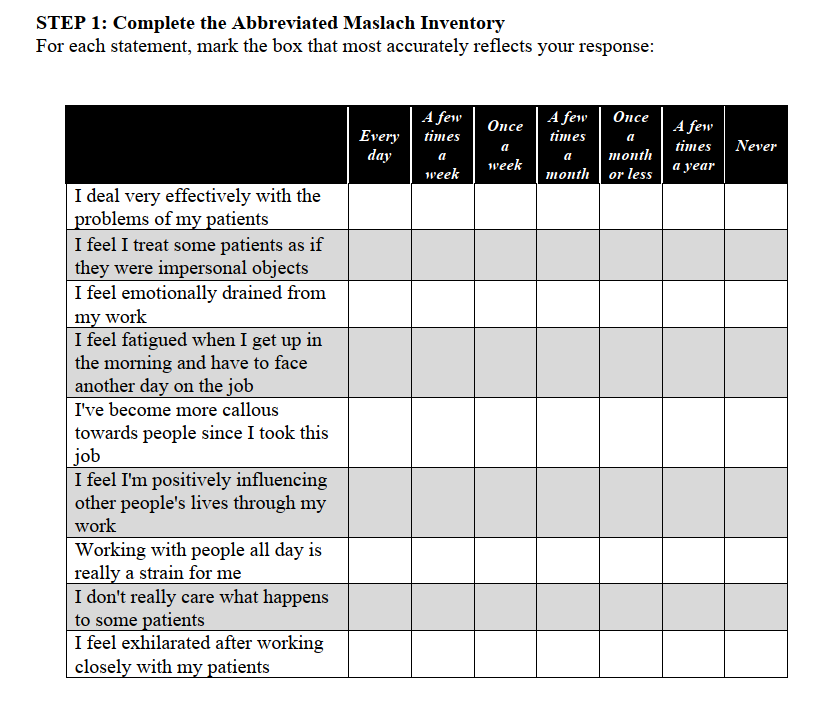


Select a number between 1-100 reflecting your current stress level:
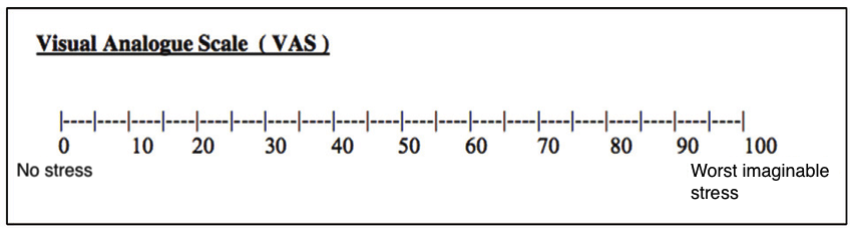

Supplement: S7 Appendix — (DOCX) [file pone.0340456.s007.docx]
