# Supplementary material for: Effect of psychological first aid training for fellows on resident burnout and distress in the intensive care unit
Source: PLoS One. 2026 Feb 9;21(2):e0340456. doi: 10.1371/journal.pone.0340456 (PMC12885303; doi:10.1371/journal.pone.0340456)
Supplement: S4 Appendix — (PDF) [file pone.0340456.s004.pdf]

Parkland

From: Susan Partridge
Vice President, Research Administration
Human Subjects Protection Administrator

RE: Acknowledgement of QA/QI – Non- Human Subject Research

Project: Implementation and evaluation of a quality improvement program to improve mental health of physicians in the medical intensive care unit

Project Lead: Traci Adams, MD Assistant Professor of Medicine, UT Southwestern

Dear Dr. Adams,

Your project has been reviewed by the Parkland Office of Research Administration (ORA) and it has been determined that it is a quality project to implement a psychological first aid curriculum to improve the mental health of residents, fellows, and faculty. This project does not meet the definition of human subjects research with 45 CFR 46.102. Therefore, approval from the Institutional Review Board (IRB) and the Parkland ORA is not required.

Please note that projects that utilize Parkland data and protected health information (PHI) must utilize good data stewardship practices and maintain the security, limited access and confidentiality of data used for this project.

Any reports, presentations or publications that result from this project should make clear that the project is not research with the findings to improve the mental health of the residents, fellows and faculty by engaging in mental health problems in ICU providers with an overview of psychological first aid that has been a well establish, consensus- based form of mental health assistance provided in immediate aftermath of disasters to address acute distress and re-establish coping and functioning. If there are any changes to the project or interest in presenting the results as "research," then approval from the IRB and the Parkland ORA will be needed.

If you have any questions about this determination, please don't hesitate to contact the Parkland Office of Research Administration or the IRB.

Sincerely,

Susan Partridge

Susan Partridge

12/20/2023
Date

Cc: Gretchen Blake, VP Quality and Clinical Effectiveness, Parkland
Patty Brown, Manager Performance Improvement, Parkland
Allison Beaver, RN, Director Research Compliance, Parkland

Parkland Health
8435 Stemmons Fwy. Suite 1000
Dallas, Texas 75247
Telephone: 214-590-1170
Fax : 214-590-4167

UT Southwestern
Medical Center

Human Research Protection Program

December 19, 2023

To: Traci Adams
Assistant Professor of Medicine

From: Stephanie Francisco, CIP
Sr. Regulatory Analyst
Human Research Protection Program Office

RE: Non-Regulated Research

Project Title: Implementation and evaluation of a quality improvement program to improve mental health of physicians in the medical intensive care unit

The UT Southwestern Human Research Protection Program (HRPP) has reviewed the above referenced project and determined that it does not meet the definition of research under 45 CFR 46.102 and therefore does not require IRB approval or oversight.

If you have any questions related to this communication or the UT Southwestern HRPP, please call 214-648-3060.

DocuSigned by:
Stephanie Francisco CIP
0AC9472F165C44D...

Form Y2
UT Southwestern Medical Center
Request for Determination That a Proposed Activity is Not Research Requiring IRB Review

**1. Date:**	**11/9/23**

**2. Title:**	Implementation and evaluation of a quality improvement program to improve mental health of physicians in the medical intensive care unit

**3. Name and Address of Project Director/Principal Investigator (PD/PI):** (This is the primary local contact information used by the HRPP. Indicate where mail can most reliably reach the PD/PI.)	
PD/PI Name	Traci Adams
Employer(s):	UT Southwestern
Department:	Medicine
PD/PI's Telephone #	832-428-8315
PD/PI's e-mail address:	Traci.adams@utsouthwestern.edu
PD/PI's Position Title:	Assistant Professor of Medicine
Point of contact name:	Traci Adams
Point of contact email	See above
Point of contact telephone #:	See above

**4. Project Sites - List all sites where your project will occur**	
**Check all that apply**	**Name of Institution / Site** *(list all participating sites below)*
☑	**UT Southwestern** ***(Insert Department or Clinic)*: Intensive care unit (**Department of Medicine)
☑	**Parkland HHS (PHHS)** ***(Insert Department or Clinic)*:** Intensive care unit (department of medicine)
☐	**Children's Health** ***(Insert Department or Clinic)*:**
☐	**Texas Scottish Rite** ***(Insert Department or Clinic)*:**
☐	**Retina Foundation of the Southwest** ***(Insert Department or Clinic)*:**
☐	**Texas Health Resources (THR) Hospital** ***(Insert Department or Clinic)***
☐	**Other →** ***(Insert Department or Clinic)*:**

5: Is this regulated human research requiring IRB approval?

*If you answer **NO** to all questions below, then IRB review and approval is likely **NOT** required. Upon review, the Human Research Protection Program Office (HRPPO) will issue you with a determination letter that your research does not require IRB review and approval.*

*In an effort to avoid unnecessary work, if you are uncertain or if you answer **Yes** to any question below, **STOP** and call the HRPP office at 214-648-3060 for further guidance.*

	Yes	No
1. Is the intent of the project either to test a novel hypothesis or to replicate another researcher's original study?	☐	☑
2. Will patients or personnel be exposed to additional discernable risks or burdens beyond those of **usual care** at this institution?	☐	☑
3. Does the project involve withholding of any aspect of conventional care shown to be beneficial in prior well-conducted clinical trials?	☐	☑
4. Does the project seek to test interventions, practices or treatments that are not standard of care (neither consensus-based nor evidence-based)?	☐	☑
5. Will the UTSW or an affiliated institution receive a direct federal (DHHS) award to conduct human subjects' research, even where all activities involving human subjects are carried out by a non-UTSW entity (e.g., subcontractor or collaborator)? *Research Funding from the Department of Health and Human Services (DHHS) (e.g., Agency for Healthcare Research and Quality (AHRQ); Centers for Disease Control and Prevention (CDC); National Institutes of Health (NIH); etc.)*	☐	☑
6. Does the project involve a drug or device used outside of usual medical practice, including non-FDA-approved agents, or off-label uses of FDA-approved drugs or devices?	☐	☑
7. Will the safety and/ or effectiveness of a drug (FDA approved or non-FDA approved) or regulated device be evaluated or be compared to that of another?	☐	☑
8. Will data from the activity of an active group or a control group be submitted to, or held for inspection by the FDA in support of a marketing or research application for an FDA-regulated product (drug or device)?	☐	☑
9. Does the project have funding from an organization with a commercial interest in the use of the results?	☐	☑
10. Will data obtained from use of a device on tissue specimens be submitted to, or held for inspection by, the FDA in support of a marketing application or research application for an FDA regulated product?	☐	☑

6. Classify your activity (Check the best choice)	
	**Activities Not Considered Research**
☐	**Health surveillance**. Health surveillance is an ongoing part of the medical care and public health care functions closely integrated with timely **dissemination of these data to those responsible for preventing and controlling disease or injury** (may include emergent or urgently identified or suspected imminent health threats to the population to document the existence and magnitude).
☑	Routine **Quality Improvement** (QI) means systematic, data-guided activities designed to bring about immediate, positive changes in the delivery of health care in particular settings. QI involves deliberate actions to improve care, guided by data reflecting the effects (e.g., types of practical problem solving; an evidence-based management style; the application of science of how to bring about system change; review of aggregate data at the patient/provider/unit/ organizational level to identify a clinical or management change that can be expected to improve care).<br><br>For QI – answers to the following questions should be **YES**:<br>1. Are patients who receive the project intervention expected to benefit?<br>2. Will all groups in the project receive, at the minimum, usual care at this institution?<br>3. Is the purpose to measure the performance of or to determine the effect of a process change intended to improve health care delivery?<br>4. Will the results be used to inform and implement improvements in patient care at the institution the process is being implemented?
☐	**Medical quality assurance**. This refers to activities particular to an institution's QA program, such as those activities protected from disclosure as part of its confidential medical quality-assurance program or other equivalent programs. (e.g., see applicable university or institutional policy)
☑	**Program evaluation**. This refers to assessments of the success of established programs in achieving objectives when the assessments are for the use of program managers, for example, a survey to determine if program beneficiaries are aware of the availability of program services or benefits. [Note: Non-research evaluation is generally designed to assess or improve the program or service rather than to generate knowledge about a disease or condition.]
☐	**Customer satisfaction surveys**. This refers to surveys of program users to obtain feedback for use by program managers. This is similar to program evaluation.
☐	**Academic Projects**: academic projects or student assignments involving collection of data from human subjects when the data is used solely for the purpose of learning research methods and not intended to be used to develop or contribute to generalizable knowledge.
☐	**Case Reports**: use medical information collected from a clinical activity rather than a research activity and presented on no more than three (3) patients. Case reports are generally done by retrospective review of the medical record and highlights a unique treatment, case or outcome. The examination of the case is usually not systematic and there is usually no data analysis or testing of a hypothesis. Investigators must ensure that the HIPAA privacy rules are followed with respect to using or accessing PHI (a HIPAA authorization or waiver may be required)
☐	**Community Outreach**: The primary intent of research is to generate or contribute to generalizable knowledge. The primary intent of non-research community outreach activity is to prevent or control disease or injury and improve health, or to improve an ongoing community outreach program or service. Knowledge may be gained in any community outreach endeavor designed to prevent disease or injury or improve a program or service. In some cases, that knowledge may be generalizable, but the primary intention of the endeavor is to benefit patients participating in an outreach health program or a population by controlling a health problem in the population from which the information is gathered.
☐	**Other**: Describe here →

7. Summary of the Activity:
Provide a summary of the proposed activity. Provide sufficient detail for the reviewer to verify whether or not the activity is research and if research, whether or not it is "human research" requiring IRB approval as you have indicated above. If a separate activity description/written plan is available, attach it to this document.

Burnout and psychiatric disease are common among physicians. The intensive care unit is an emotionally demanding environment, and burnout scores are particularly high for critical care physicians. Psychological first aid is a well-established, consensus-based form of mental health assistance provided in immediate aftermath of disaster to address acute distress and re-establish coping and functioning. It was developed through expert consensus. It includes 10 elements: being there (physical support and engagement), safety and acute stabilization, supportive listening and information gathering, education and reassurance, coping and stress management, problem solving, connecting with support, symptom management, knowing when more health is needed, and caring for caregivers.

Our purpose is to implement a psychological first aid curriculum in the medical intensive care units at Clements University Hospital in order to improve the mental health of our residents, fellows, and faculty. We plan to evaluate the curriculum as well using pre- and post-intervention surveys. Psychological first aid is not a novel curriculum or program, and the need for mental health support in the ICU has been established in the literature.

At the beginning of their medical ICU rotation at Parkland and Clements, residents, fellows, and faculty who wish to participate will fill out a brief anonymous demographic information form including information about gender, age, race/ethnicity, marital status, number of children in the family, level of education, and years working in the field. They will also complete a visual analog scale of distress and an abbreviated Maslach burnout inventory within 72 hours of starting and within 72 hours of completing an ICU rotation. This baseline data will be collected for ICU rotations for 3 months prior to implementation of psychological first aid.

The psychological first aid activity will begin with a training of pulmonary fellows and palliative care attendings who are already embedded in ICU teams in psychological first aid. This training will be conducted by a prominent trauma psychiatrist with over 400 publications and a licensed clinical social worker and will be a hands-on half day session based on established best practices for psychological first aid. The training will be adapted from the CALM program for UTSW faculty participating in peer to peer psychological support. Feedback about the presentation will be collected via surveys and a brief discussion before and after the training.

During the course of their rotation, psychological first aid will be offered by pulmonary fellows to residents and by palliative care faculty to pulmonary faculty and fellows. Residents, fellows, and faculty who do not wish to participate do not have to engage in discussions. During the course of the rotation, one of the standard MICU lectures will cover mental health problems in ICU providers and an overview of psychological first aid. We will also provide documents with contact information for therapists, psychiatrists, and wellness resources available to UTSW physicians. Psychological first aid may include one on one discussions, group discussions, education, connection with formal counselors and psychiatrists, reassurance, destigmatizing mental health, and skilled listening. The individuals administering psychological first aid will not evaluate the recipients in medhub. We plan to implement psychological first aid on an ongoing basis in the intensive care units and integrate this into the fellowship curriculum, but we will collect data to determine the effectiveness of this intervention for 6 months.

Quantitative data will be entered into a password-protected Excel sheet for analysis.

Recruitment. All residents, fellows, and faculty working in the Parkland or Clements University Hospital intensive care unit will be asked to complete the survey at the beginning and end of their ICU rotations.

References

Psychological First Aid Field Operations Guide, National Child Traumatic Stress Network and National Center for PTSD, 2nd ed. https://www.ptsd.va.gov/professional/treat/type/PFA/PFA_2ndEditionwithappendices.pdf

Form N
Request to Enroll Students/Residents

eIRB tracking number:	STU# insert here

Information about recruiting/enrolling Students and Residents:

Residents and students at UT Southwestern are a vulnerable population. Specifically, the IRB considers:

- *Voluntariness.* The IRB considers possibility of coercion and undue influence
- *Equitable subject selection.* The IRB disapproves inclusion of this population simply due to convenience/availability.
- *Safeguards.* – What is the plan to minimize impact on coursework or academic requirements?

To request inclusion of this population, complete this form, and have it signed by the appropriate authorities listed below. Once you have received all required signatures, submit the form with your eIRB application.

1. Which of the following Student/Resident population(s) do you plan to target?

	**Request to enroll:** (*Select all that apply*)	**Submit this completed form *and* a copy of the protocol/recruitment plan by email to:**
☐	Medical Students	Angela Mihalic, MD: Angela.Mihalic@UTSouthwestern.edu<br>Dean of Med Students & Assoc Dean Student Affairs, UTSW Med
☐	Health Professions Students	Scott Smith, PhD: Scott.Smith@UTSouthwestern.edu<br>Office of Dean- UT Southwestern School of Health Professions
☐	Graduate Students or Postdoctoral Fellows	Andrew Zinn, MD, PhD: Andrew.Zinn@UTSouthwestern.edu<br>Dean, UT Southwestern Graduate School of Biomedical Sciences
☒	Residents/Clinical Fellows	Michael Green, MD: Michael.Green@UTSouthwestern.edu<br>Assistant Dean, Office of the Dean - Southwestern Medical School

2. Approval signature(s):

☐ I have reviewed the study protocol and I support the plan to recruit **Medical Students**.

Signed: ______________________________

Dr. Angela Mihalic

☐ I have reviewed the study protocol and I support the plan to recruit **Health Professions students**.

Signed: ______________________________

Dr. Scott Smith

☐ I have reviewed the study protocol and I support the plan to recruit **Graduate students**.

Signed: ______________________________

Dr. Andrew Zinn

☒ I have reviewed the study protocol and I support the plan to recruit **Residents/Fellows**.

Signed: Michael Green ______________________________

Dr. Michael Green

Project Proposal: For Research or Non-Research Determination

Submission to Parkland Office of Research Administration

**Date of Submission**	**12/5/23**	**Planned Start Date**	**1/2/24**	**Planned Completion Date**	**10/1/2024**
**Project Lead, Title, Institution**	**Traci Adams, MD<br>Assistant Professor of Medicine<br>UT Southwestern Medical Center**		**Faculty/Department Sponsor, Institution (if applicable)**		
**Project Title**	Implementation and evaluation of a quality improvement program to improve mental health of physicians in the medical intensive care unit				
**Study Sponsor (if applicable)**	**N/A**		**Is this project funded by an external entity? If yes, specify:**	**No**	
**Purpose and Objectives**	Our purpose is to implement a psychological first aid curriculum in the medical intensive care units at Clements University Hospital in order to improve the mental health of our residents, fellows, and faculty. We plan to evaluate the curriculum as well using pre- and post-intervention surveys. Psychological first aid is not a novel curriculum or program, and the need for mental health support in the ICU has been established in the literature.				
**Is this project based on established best practices?**	**_x__ Yes<br>___ No**	**If yes, describe the best practices and source:**<br><br>Psychological first aid is a well-established, consensus-based form of mental health assistance provided in immediate aftermath of disaster to address acute distress and re-establish coping and functioning. It was developed through expert consensus. It includes 10 elements: being there (physical support and engagement), safety and acute stabilization, supportive listening and information gathering, education and reassurance, coping and stress management, problem solving, connecting with support, symptom management, knowing when more health is needed, and caring for caregivers.<br><br>The CALM program from which our psychological first aid training is derived is an established course through the Center for Patient Safety and is currently administered to UT Southwestern faculty who wish to participate in the peer to peer support program.<br><br>*Psychological First Aid Field Operations Guide, National Child Traumatic Stress Network and National Center for PTSD, 2nd ed. https://www.ptsd.va.gov/professional/treat/type/PFA/PFA_2ndEditionwithappendices.pdf*			
**Is this project based on evidence-based literature?**	**__x_ Yes<br>___ No**	**If yes, provide literature source:**<br><br>1. West CP, Dyrbye LN, Erwin PJ, Shanafelt TD. Interventions to prevent and reduce physician burnout: a systematic review and meta-analysis. *Lancet* 2016; 388: 2272-2281.<br>2. Brower KJ. Professional Stigma of Mental Health Issues: Physicians Are Both the Cause and Solution. *Acad Med* 2021; 96: 635-640.			

Covid-19 pandemic. *Chest* 2023.
4. Pfefferbaum B, North CS. Mental Health and the Covid-19 Pandemic. *N Engl J Med* 2020; 383: 510-512.
5. Maslach C, Jackson SE, Leiter MP. Maslach burnout inventory. Scarecrow Education; 1997.
6. North C.S HBA, Pepe P.E. Staff burnout in the intensive care unity during major catastrophes: the disaster "cascade". *ICU Management* 2007; 7: 10-13.
7. Farrell CM, Hayward BJ. Ethical Dilemmas, Moral Distress, and the Risk of Moral Injury: Experiences of Residents and Fellows During the COVID-19 Pandemic in the United States. *Acad Med* 2022; 97: S55-S60.
8. Moss M, Good VS, Gozal D, Kleinpell R, Sessler CN. An Official Critical Care Societies Collaborative Statement: Burnout Syndrome in Critical Care Healthcare Professionals: A Call for Action. *Crit Care Med* 2016; 44: 1414-1421.
9. Coetzee SK, Klopper HC. Compassion fatigue within nursing practice: A concept analysis. *Nurs Health Sci* 2010; 12: 235-243.
10. Kerlin MP, McPeake J, Mikkelsen ME. Burnout and Joy in the Profession of Critical Care Medicine. *Crit Care* 2020; 24: 98.

**Is the intent of this project to learn new information?**	___ Yes<br>_x__ No	**If yes, describe:**<br>**The intent of the project is not to learn new information. It is to implement an established program of psychological first aid already in use at UT Southwestern in the ICU.**		
**Will this project directly benefit PHHS patients?**	___ Yes<br>__x_ No	**If yes, describe:**		
**If this proposed project is non-research, how will the results benefit Parkland?**	The results could benefit Parkland residents, fellows, and faculty by improving mental health and decreasing burnout rates. Burnout has been associated with higher worker turnover and decreased productivity. Reducing these by supporting mental health may benefit Parkland.			
**Project goals/aims**	1. Implement a psychological first aid program for providers in the Parkland and Clements Medical ICU<br>As measured by: We will not measure the implementation of the program<br>2. Evaluate the success of the training program As measured by: surveys among participants in the CALM training<br>3. Evaluate the effect of psychological first aid on residents, fellows, and faculty<br>As measured by: pre- and post- surveys of distress and burnout			
**What PHHS data will be needed?**	No data will need to be extracted from the medical record, as this is an intervention for providers, not for patients.			
**Are additional PHHS research resources needed?**	No additional resources are needed			
**What PHHS clinical or service department(s) will be impacted?**	**Medical ICU**	**Name of the PHHS department manager(s) who approved**	**Bethany Lussier (Parkland MICU director)**	
**Where will the results of this project be shared? (presentations, publications)**	Presentations, possibly publications	**Will your presentation or publication describe the project as research?**	__ Yes<br>_x_ No	

**Summary of the Activity:**<br>Attach a detailed summary of your project or describe below what proposed activity(ies) you plan to implement to accomplish your goals/aims.	At the beginning of their medical ICU rotation at Parkland and Clements, residents, fellows, and faculty who wish to participate will fill out a brief anonymous demographic information form including information about gender, age, race/ethnicity, marital status, number of children in the family, level of education, and years working in the field. They will also complete a visual analog scale of distress and an abbreviated Maslach burnout inventory within 72 hours of starting and within 72 hours of completing an ICU rotation. This baseline data will be collected for ICU rotations for 3 months prior to implementation of psychological first aid.<br><br>The psychological first aid activity will begin with a training of pulmonary fellows and palliative care attendings who are already embedded in ICU teams in psychological first aid. This training will be conducted by a prominent trauma psychiatrist with over 400 publications and a licensed clinical social worker and will be a hands-on half day session based on established best practices for psychological first aid. The training will be adapted from the CALM program for UTSW faculty participating in peer to peer psychological support. Feedback about the presentation will be collected via surveys and a brief discussion before and after the training.<br><br>During the course of their rotation, psychological first aid will be offered by pulmonary fellows to residents and by palliative care faculty to pulmonary faculty and fellows. Residents, fellows, and faculty who do not wish to participate do not have to engage in discussions. During the course of the rotation, one of the standard MICU lectures will cover mental health problems in ICU providers and an overview of psychological first aid. We will also provide documents with contact information for therapists, psychiatrists, and wellness resources available to UTSW physicians. Psychological first aid may include one on one discussions, group discussions, education, connection with formal counselors and psychiatrists, reassurance,		

	The individuals administering psychological first aid will not evaluate the recipients in medhub. We plan to implement psychological first aid on an ongoing basis in the intensive care units and integrate this into the fellowship curriculum, but we will collect data to determine the effectiveness of this intervention for 6 months.<br><br>Quantitative data will be entered into a password-protected Excel sheet for analysis.<br><br>Recruitment. All residents, fellows, and faculty working in the Parkland or Clements University Hospital intensive care unit will be asked to complete the survey at the beginning and end of their ICU rotations.		

Abbreviated Maslach Burnout Inventory

STEP 1: Complete the Abbreviated Maslach Inventory
For each statement, mark the box that most accurately reflects your response:

	Every day	*A few times a week*	*Once a week*	*A few times a month*	*Once a month or less*	*A few times a year*	*Never*
I deal very effectively with the problems of my patients							
I feel I treat some patients as if they were impersonal objects							
I feel emotionally drained from my work							
I feel fatigued when I get up in the morning and have to face another day on the job							
I've become more callous towards people since I took this job							
I feel I'm positively influencing other people's lives through my work							
Working with people all day is really a strain for me							
I don't really care what happens to some patients							
I feel exhilarated after working closely with my patients							

"Stress, burnout and doctors' attitudes to work are determined by personality and learning style: A twelve year longitudinal study of UK medical graduates." McManus IC, Keeling A, Paice E. *BMC Medicine* 2004, 2:29 doi:10.1186/1741-7015-2-29

"Early predictors of job burnout and engagement." Maslach C, Leiter MP. *J Appl Psychol.* 2008 May;93(3):498-512. doi: 10.1037/0021-9010.93.3.498

STEP 2: Determine your score for the Abbreviated Maslach Inventory
Use this sheet to determine your score for the statements filled out on the previous page. Then, add up your scores by color, and refer to the descriptions below. Categories are rated on a scale from 0-18.

	Every day	*A few times a week*	*Once a week*	*A few times a month*	*Once a month or less*	*A few times a year*	*Never*	TOTAL
I deal very effectively with the problems of my patients	6	5	4	3	2	1	0	
I feel I treat some patients as if they were impersonal objects	6	5	4	3	2	1	0	
I feel emotionally drained from my work	6	5	4	3	2	1	0	
I feel fatigued when I get up in the morning and have to face another day on the job	6	5	4	3	2	1	0	
I've become more callous towards people since I took this job	6	5	4	3	2	1	0	
I feel I'm positively influencing other people's lives through my work	6	5	4	3	2	1	0	
Working with people all day is really a strain for me	6	5	4	3	2	1	0	
I don't really care what happens to some patients	6	5	4	3	2	1	0	
I feel exhilarated after working closely with my patients	6	5	4	3	2	1	0	

Red = Emotional Exhaustion
Total: ______ (0-18)
Higher scores indicate greater emotional exhaustion, and greater burnout.

Blue = Depersonalization
Total: ______ (0-18)
Higher scores indicate greater depersonalization, and greater burnout.

Green = Personal Accomplishment
Total: ______ (0-18)
Higher scores indicate greater personal accomplishment, and *less* burnout.

Please circle one answer for each of the following questions Date ___________

Gender: Male Female

Age: ________

Race: White Black Other (fill in) ______________________

Hispanic Ethnicity: Yes No

Marital status: Married Not married

(If not married): Cohabitating with significant other: Yes No

Number of children living with you: ____________

Position: Attending Fellow Resident Intern

(if Attending) Faculty rank: Assistant professor Associate professor Full professor

(If resident): Which residency program are you in? UTSW IM residency Other(fill in) ________

(If IM resident): Do you plan to pursue a fellowship in pulmonary/critical care? Yes No

(If IM resident): Do you plan to pursue a fellowship in cardiology? Yes No

(If faculty) Primary practice site: CUH Parkland

Year started residency: ____________

Year started pulmonary and critical care fellowship: __________

Year started on faculty at UTSW: ____________

(If faculty) % Administrative Time (significant administrative role, not including the 15% academic time):

____

Administrative role title(s): ____________________________

Do you know how to access psychiatric assistance at UTSW? Yes No

Do you know any healthcare workers at UTSW who could not access psychiatry care in a timely manner?
Yes No

Time spent in MICU this academic year: < 1 month 1 month – 3 months > 3 months

Is this your first ever MICU rotation? Yes No
